# The ubiquitin codes in cellular stress responses

**DOI:** 10.1093/procel/pwad045

**Published:** 2023-07-20

**Authors:** Xiangpeng Sheng, Zhixiong Xia, Hanting Yang, Ronggui Hu

**Affiliations:** Key Laboratory of Systems Health Science of Zhejiang Province, School of Life Science, Hangzhou Institute for Advanced Study, University of Chinese Academy of Sciences, Hangzhou 310024, China; State Key Laboratory of Molecular Biology, Shanghai Institute of Biochemistry and Cell Biology, Center for Excellence in Molecular Cell Science, Chinese Academy of Sciences, Shanghai 200031, China; State Key Laboratory of Animal Disease Control, Harbin Veterinary Research Institute, Chinese Academy of Agricultural Sciences, Harbin 150069, China; Key Laboratory of Systems Health Science of Zhejiang Province, School of Life Science, Hangzhou Institute for Advanced Study, University of Chinese Academy of Sciences, Hangzhou 310024, China; State Key Laboratory of Molecular Biology, Shanghai Institute of Biochemistry and Cell Biology, Center for Excellence in Molecular Cell Science, Chinese Academy of Sciences, Shanghai 200031, China; Department of Neurology, State Key Laboratory of Medical Neurobiology, Institute for Translational Brain Research, MOE Frontiers Center for Brain Science, Zhongshan Hospital, Fudan University, Shanghai 200032, China; Key Laboratory of Systems Health Science of Zhejiang Province, School of Life Science, Hangzhou Institute for Advanced Study, University of Chinese Academy of Sciences, Hangzhou 310024, China; State Key Laboratory of Molecular Biology, Shanghai Institute of Biochemistry and Cell Biology, Center for Excellence in Molecular Cell Science, Chinese Academy of Sciences, Shanghai 200031, China

**Keywords:** ubiquitin, E3 ligase, environmental stresses, intercellular stresses, stress response, homeostasis

## Abstract

Ubiquitination/ubiquitylation, one of the most fundamental post-translational modifications, regulates almost every critical cellular process in eukaryotes. Emerging evidence has shown that essential components of numerous biological processes undergo ubiquitination in mammalian cells upon exposure to diverse stresses, from exogenous factors to cellular reactions, causing a dazzling variety of functional consequences. Various forms of ubiquitin signals generated by ubiquitylation events in specific milieus, known as ubiquitin codes, constitute an intrinsic part of myriad cellular stress responses. These ubiquitination events, leading to proteolytic turnover of the substrates or just switch in functionality, initiate, regulate, or supervise multiple cellular stress-associated responses, supporting adaptation, homeostasis recovery, and survival of the stressed cells. In this review, we attempted to summarize the crucial roles of ubiquitination in response to different environmental and intracellular stresses, while discussing how stresses modulate the ubiquitin system. This review also updates the most recent advances in understanding ubiquitination machinery as well as different stress responses and discusses some important questions that may warrant future investigation.

## Introduction

Living organisms are never free of the stresses induced by internal or exogenous factors, as constant changes are intrinsic to all live cells that have dynamic reactions ongoing within. The changes in various environmental effectors, such as temperature, oxygen availability, salinity, pH, toxic chemicals, and infectious reagents, or the physiological alterations, including DNA damage/lesions and accumulated biomolecules [oxidative molecules, ubiquitin (Ub), and misfolded proteins], can result in the disruption of the relatively balanced status of biological or chemical molecules or cellular networks, causing stresses at cellular, tissue, or organismal levels (Galluzzi et al. 2018). Maintaining homeostasis is fundamental for cells to survive under different stresses. Cells possess complicated and effective responses to defend against and recover from stresses. Once the noxious stress is prolonged and unresolved or the corresponding cellular response is disrupted, stressed cells could face severe damages even death. Regulation of gene transcription and translation is one of the most basic strategies that cells have evolved to maintain homeostasis (Spriggs et al. 2010). Moreover, post-translational modifications (PTMs) (including phosphorylation, ubiquitination, acetylation, etc.) are dynamic and reversible strategies that cells employ to alter the functionality of specific signaling pathways in response to different stresses, and direct cells to different fates. It is noteworthy that PTMs also play critical roles in controlling gene transcription and expression. Thus, understanding the roles of the PTMs in stress responses and developing proper interventions, if possible, would therefore provide an increased number of avenues to maintain cellular homeostasis, and may ultimately bring about beneficial clinical outcomes.

Ubiquitination is one of the most ubiquitous and crucial PTMs in normal homeostasis and diseases, targeting thousands of substrates and controlling the majority of physiological processes, such as gene transcription, cell growth and death, DNA replication, chromatin assembly, molecule trafficking, metabolism, immune response, and development (Pickart 2001; Weissman 2001). By covalently labeling a Ub molecule or Ub chains to substrates, ubiquitination shows the ability to control stability, activity, localization, or binding partners of targeted substrates. Disruption of ubiquitination could lead to mislocalization of proteins, accumulation of damaged or misfolded proteins, improper complex assembly, aberrant enzymatic activities, or inaccurate signal transductions, contributing to the development of human diseases, including cancers, autoimmune diseases, developmental disorders, metabolic syndromes, and neurodegeneration (Popovic et al. 2014; Rape 2018).

Although ubiquitination has displayed regulatory roles in different cellular stress responses, it is still not been systematically summarized. In the following sections, we will introduce the ubiquitination system first, and discuss the important roles of ubiquitination in regulating cellular responses to various environmental stressors (hypoxia, heat or cold shock, and osmotic stress) or intercellular stressors (DNA damage, Ub stress, ER stress, and oxidative stress). Notably, one environmental stressor may induce one or more intercellular stresses, and an intercellular stress can lead to other intercellular stresses. As one stress is usually associated with others, some cellular responses could regulate and resist multiple stresses.

## The ubiquitination system

### Ubiquitin

**Figure 1.**
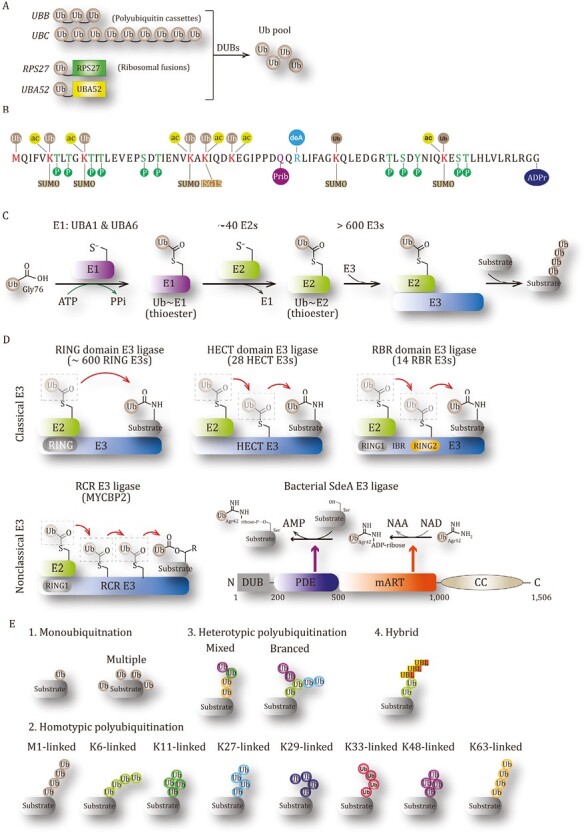
Ub and ubiquitination system. (A) Genes encoding Ub molecule. (B) PTMs on ubiquitin. (C) The ATP-dependent ubiquitination process. (D) E3 Ub ligases having different ubiquitin-transfer mechanisms. (E) Linkages of Ub chains.

Ub, a small and highly stable protein with 76 amino acids, is named for its extremely ubiquitous distributions in all eukaryotic cells. Ub was first isolated from the thymus in 1975 by Goldstein et al. and was found to form an isopeptide linkage with histone 2A in 1977 by Goldknopf and Busch (Goldknopf and Busch, 1977; Hershko and Ciechanover 1998). There are four genes in human encoding Ub molecules (Fig. 1A). Gene *UBA52* and *RPS27A* encode two N-terminal Ub moieties that are fused to ribosomal protein L40 and S27a. The monomeric Ub that is cleaved off from fusion proteins by deubiquitinating enzymes (DUBs) sustains the total Ub pool in normal conditions. Moreover, gene *UBB* and *UBC* encode PolyUb precursors with 3 and 9 repeats, respectively, rapidly increasing Ub concentration in cells under stress (Finley et al. 1987; Rape 2018). These two fusion precursors are also cleaved by specific DUBs to release free Ub molecules. Apart from being a modifier, Ub can be targeted by multiple PTMs (Fig. 1B) (Swatek and Komander 2016). In particular, conjugation of Ub to lysine residues (K6, K11, K27, K29, K33, K48, and K63) or N-terminal methionine of Ub itself generates different PolyUb chain linkages, which determines the destiny of substrates (Mansour 2018). SUMO, ISG15, and NEDD8 can also modify the lysine residues of Ub (Liao et al. 2022). Moreover, small chemical groups can covalently attach Ub to form phosphorylation, acetylation, deamidation, ADP ribosylation, and phospho-ribosylation of Ub (Mattiroli and Penengo 2021). While all the Ser, Thr, and Tyr residues on Ub can be phosphorylated, 6 out of 7 Lys residues of Ub undergo acetylation (Mattiroli and Penengo 2021). Recent reports established that a specific arginine of Ub can undergo phospho-ribosylation (Bhogaraju et al. 2016; Qiu et al. 2016). Modified Ub could act as different signaling molecules to regulate cellular activities. The new layers of Ub modifications exponentially increase the complexity and functionality of the Ub system, which is part of the so-called Ub codes.

The structure of Ub includes an β-grasp fold with a C-terminal flexible tail and several hydrophobic surfaces, which is highly conservative from yeast to human (Vijay-Kumar et al. 1987). A variety of ubiquitin-binding domains (UBDs) are presented in numerous cellular proteins (>150) and recognize the hydrophobic surfaces, especially Ile44 patch, of Ub, triggering functional events (Dikic et al. 2009). Noteworthy, UBB + 1, a frameshift Ub mutant, bearing a 19-amino-acid extension at the C-terminus, emerges in aging cells and is readily utilizable by the Ub machinery. The presence and accumulation of UBB + 1 can ultimately impact the activity of the Ub-proteasome system (UPS) (Van Leeuwen et al. 1998; Lam et al. 2000), and probably other cellular processes as well. UBB + 1 expression leads to long-term proteasome dysfunction and memory deficits in mice, suggesting a pathogenic role in the etiology of Alzheimer’s disease (AD) (Fischer et al. 2009; Tank and True 2009).

#### Ubiquitin-like proteins

Ubiquitin-like proteins (UBLs) belong to a protein family whose members shares a conserved globular β-grasp conformation similar to Ub. UBLs can modify substrates via a similar enzymatic cascade (Kerscher et al. 2006). As UBLs members, SUMO conjugates substrates for sumoylation, NEDD8 for neddylation, ISG15 for Isgylation, UFM1 for ufmylation, URM1 for urmylation, FAT10 for FATylation, and ATG8 or ATG12 for ATGylation. UBL conjugations regulate the stability, activity, molecular interactions, or localization of target substrates, which greatly increases the diversity and dynamics of proteome (Cappadocia and Lima 2018).

### Ubiquitination

Ubiquitination is a specific three-step enzymatic cascade that covalently attaches Ub to substrates. Typically, the C-terminal glycine residue of Ub is conjugated to the ε-amino group of lysine residue(s) of substrate protein by forming an isopeptide bond (Komander 2009). The process of labeling substrate with Ub is cooperatively performed by at least three types of enzymes: Ub-activating enzyme (E1), Ub-conjugating enzyme (E2), and Ub ligase (E3) (Weissman 2001) (Fig. 1C). First of all, E1 activates free Ub via catalyzing the formation of a high-energy thioester bond between its catalytic cysteine residue and the C-terminal carboxyl group of Ub, in an ATP-dependent manner. In the second step termed E1–E2 thioester transfer, E1 submits the activated Ub to the catalytic cysteine residue of an E2. Finally, E3 ligases act as adaptors that specifically recruit both the Ub-charged E2 and substrate, leading to Ub transfer and the formation of isopeptide bond between Ub and the lysine residue(s) of the substrate (Olsen and Lima 2013; Liao et al. 2022). Once the ubiquitination process is finished, the stability or biological function of the target will be altered. In humans, there are eight E1 enzymes, but only two E1s (UBA1 and UBA6) are known to initiate the conjugation of Ub (Schulman and Wade Harper 2009). To date, around 40 E2 proteins and more than 600 E3 ligases are encoded by the human genome (Liu et al. 2019a; Liao et al. 2022). Besides the Ub carriers that mainly exist as E2~Ub conjugates (Ye and Rape 2009), E2s are now considered to play critical roles in controlling Ub chain assembly in the ubiquitination process [for a special review of E2, see (Stewart et al. 2016)]. E3 ligases have the capacity to selectively recognize substrates, determining the specificity of ubiquitination; E3 contains a large number of members and catalyzes different Ub linkages on substrates, which increases the diversity of ubiquitination.

#### Nonclassical ubiquitination

The lysine residue is the canonical site for ubiquitination. Although lysine was previously thought to be the only ubiquitination site for decades, no-lysine ubiquitination on other amino acids has been firmly demonstrated (McClellan et al. 2019). Nonclassical ubiquitination on cysteine, serine, and threonine residues of special substrates has been reported to modulate a variety of physiological activities (Squair and Virdee 2022). Moreover, the free amino group of the translation-initiating methionine or the amino acid at the second position (in the case of methionine removal) of substrate can act as the site of N-terminal ubiquitination (Akimov et al. 2018).

Protein has been considered as the only substrate for ubiquitination all the time, but two recent studies extend ubiquitination substrates beyond protein. Otten et al. first reported the ubiquitination of a non-proteinaceous substrate (Otten et al. 2021). During *Salmonella* infection, host E3 ligase RNF213 catalyzes the ubiquitination of the lipid A moiety of bacterial lipopolysaccharide (LPS), which relies on the RZ-finger domain but not the RING domain of RNF213, thus triggering cell-autonomous immunity. During the preparation of this review, a new research was published to show that eukaryotic phospholipids, mainly phosphatidylethanolamine (PE) presented in endosomes and lysosomes, were ubiquitinated by an E3 ligase Tul1 and deubiquitinated by Doa4, which regulates recruitment of ESCRT components (Sakamaki et al. 2022). Therefore, lipid is emerging as a new class of substrates beyond protein for ubiquitination.

#### DUBs

As a reversible reaction, ubiquitination is counteracted by DUBs. DUBs cleave the isopeptide bond and catalyze Ub removal from substrates. Mammalian cells express nearly 100 putative DUBs that are classified into two classes, cysteine proteases and zinc metalloproteases, according to different catalytic mechanisms they possess (Trulsson et al. 2022). Most mammalian DUBs (~90) are the members of cysteine proteases class harboring Cys-His-Asp catalytic motif, and these cysteine protease DUBs can be further classified into six families based on their sequence similarity: UCHs, USPs, OTUs, Josephins (MJDs), and two nearly identified families MINDYs and ZUFSP/ZUP1 (Kwasna et al. 2018; Clague et al. 2019; Liu et al. 2022a). Only the 12-member JAMM family DUBs are zinc-dependent metalloproteinases (Clague et al. 2019). DUBs have the selectivity to recognize and cleave particular Ub linkages and maintain ubiquitome homeostasis in cells. DUBs are well known for their antagonistic roles in regulating ubiquitination under stressful conditions, but they will not be discussed in detail here because of space limitations.

### E3 Ub ligases

E3 ligases specifically recognize substrates and control the modification process, which makes it extraordinarily critical in the ubiquitination system. As the largest group in the ubiquitination process, E3 ligases have different E2-binding structures and catalytic mechanisms of Ub transfer, and they are generally divided into three major categories: RING (really interesting new gene) domain E3 (~600 members), HECT (homologous to E6AP carboxyl terminus) domain E3 (28 members), RBR (RING-between-RING) domain E3 (14 members) (Fig. 1D). While RING E3s catalyze the direct Ub transfer from E2 to substrate, HECT and RBR E3s deliver Ub to substrate via a two-step reaction.

#### RING domain E3 ligases

Freemont *et al*. first reported the conserved RING motif in 1991 (Freemont et al. 1991). RING domain was originally thought to mediate DNA binding, but numerous studies since 1999 unambiguously establish that most RING domain proteins possess E3 activity (Deshaies and Joazeiro 2009). It is worth noting that not every single RING domain can catalyze ubiquitination. Some RING proteins, such as MDMX, BARD1, and BMI1, do not display E3 activity alone, but they can form heterodimer with other RING E3 proteins to regulate E3 activity (Linares et al. 2003). The canonical RING domain is a Zn^2+^-binding “cross-brace” structure that contains several uniquely spaced Cys/His residues: C-X_2_-C-X_9–39_-C-X_1–3_-H-X_2–3_-C/H-X_2_-C-X_4–48_-C-X_2_-C (where X is any residue), responsible for E2~Ub recruitment (Deng et al. 2020). A conserved “U-box” structure is a modified version of RING domain that lacks Zn^2+^-chelating Cys/His residues, but retains the similar RING structure stabilized by hydrogen bonds and salt-bridges (Aravind and Koonin 2000). RING E3 does not form an intermediate thioester bond with Ub, but acts as a scaffold to simultaneously recruit E2 and the substrate by different domains, finally facilitates Ub transfer from E2 to substrate.

Nearly 600 E3s, which is ~95% of total human E3s, are RING domain E3s. RING E3s can be categorized into four types according to their oligomeric patterns for function: monomers, homodimers, heterodimers, and multi-subunit complexes (Rennie et al. 2020). Some RING E3s can recruit E2 and substrate in the monomeric form, e.g., CBL-B, EL5, RBX1, RNF168, and E4B (U-box) (Dou et al. 2013). Many RING E3s often form dimer via the RING domain or surrounding motifs, which generate homodimers, such as BIRC7, RNF4, cIAP (BIRC2), TRIM5α, TRAF2, and CHIP (U-box), or heterodimers in which only one subunit binds to E2, such as RING1b-BMI1, BRCA1-BARD1, and Mdm2-MdmX (Morreale and Walden 2016; Balaji and Hoppe 2020). Moreover, Some RING E3s function as a multiple-subunit complex, such as the cullin-RING ligases (CRLs) that share a similar architecture consisting of several specific subunits: a cullin protein as scaffold, a RING-box protein for E2 binding at scaffold N-terminus, an adaptor protein, and a receptor protein for substrate recognition at scaffold C-terminus (Petroski and Deshaies 2005). Anaphase-promoting complex/cyclosome (APC/C), the largest E3 Ub ligase (1.2 MDa) ever described, is a multi-subunit RING E3 comprised of total 20 subunits from 15 proteins, including a RING E3 Apc11 and a cullin-like adaptor Apc2 (Barford 2020). Some covalent modifications, Ub binding, adaptors, cofactors, or ligands can modulate the catalytic activity of RING E3s.

#### HECT domain E3 ligases

E6-associated protein (E6AP or UBE3A) has a ~350 aa C-terminal domain that catalyzes polyUb chain formation (Huibregtse et al. 1995). Homologous related to E6AP carboxyl terminus are referred to HECT E3 family, containing 28 E3 members in human (Wang et al. 2020b). All HECT E3 have a conservative C-terminal catalytic HECT domain and structurally distinct N-terminus. The HECT domain is composed of two lobes: a larger N-terminal lobe (N-lobe) responsible for E2 binding and a smaller C-terminal lobe (C-lobe) that contains the catalytic cysteine. A highly flexible hinge region links these two lobes, allowing the catalytic C-lobe to move around to transfer Ub to the substrate (Weber et al. 2019). In contrast, the N-terminal part of HECT E3s is structurally variable and primarily mediates substrate recognition. HECT E3 has three subfamilies based on the similarity of N-terminal domains. The most famous one is nine-member NEDD4 family that is characterized by the existence of a C2 domain and 2–4 WW domains at the N-terminus: NEDD4, NEDD4L, SMURF1, SMURF2, NEDL1, NEDL2, WWP1, WWP2, and ITCH (Wang et al. 2020b). The second subfamily is the HERC family that owns RCC1-like domain (RLD). This family has four small HECR E3s (HERC3, HERC4, HERC5, and HERC6) containing a single RLD domain, and two large HERCs (HERC1 and HERC2) that possess more RLD domains (Sánchez-Tena et al. 2016). Last, the remaining 13 HECT E3s are categorized as “other” HECTs because they lack WW or RLD domains and show no specific N-terminal domains, such as an AZUL domain for E6AP, an ANK domain for HACE1, and a DOC domain for HECTD3; and these different N-terminal domains are able to recognize numerous substrates (Li et al. 2020a; Singh et al. 2021). HECT E3s catalyze an intermediate thioester bond between its catalytic cysteine and the N-terminus of the transferred Ub, which is the most significant feature of HECTs (Rotin and Kumar 2009). Specifically, HECT E3s first receive Ub from E2 by its active cysteine to further form an E3~Ub covalent intermediate, and subsequently transfer Ub to a specific residue of substrate. HECT E3s have intrinsic capacity to generate linkage-specific polyUb chains, especially the heterotypic chains with mixed linkages (Sheng et al. 2017; French et al. 2021).

#### RBR domain E3 ligases

RBR E3 ligases are characterized by a RING-HECT hybrid pattern (Wang et al. 2020a). Two research groups in 1999 first identified a highly conserved triple-RING/zinc finger motif in RBR proteins (Morett and Bork 1999; Van Der Reijden et al. 1999). RBRs share a structurally similar catalytic triad, consisting of a RING1 domain, a RING2 (or Rcat) domain that does not show canonical RING E3 fold, and an IBR (In-Between-RING) domain in the middle of the RING1 and RING2 (Reiter and Klevit 2018). While RING1 is responsible for the recruitment of Ub-changed E2 and RING2 offers a catalytic cysteine, the function of the IBR domain is still under investigation. The RBR E3s-mediated ubiquitination is a sequential reaction: the E2-Ub conjugate is recruited to RING1, and Ub is subsequently transferred to the active cysteine of RING2 to form a covalent intermediate, and finally, RBRs catalyze Ub delivery from RING2 to the substrate (Wang et al. 2020a). To our knowledge, human genome encodes total 14 different RBR proteins. The best-known RBR member is Parkin whose ligase activity is associated with neurodegeneration (Pickrell and Youle 2015). Two RBR E3s (HOIL-1L and HOIP) and an adaptor SHARPIN form an E3 enzyme complex, the liner Ub chain assembly complex (LUBAC), which generates linear polyUb chains and regulates apoptosis, inflammation, angiogenesis, and immune diseases (Fu et al. 2021; Ning et al. 2022). The remaining 11 RBRs are Ariadne E3s (ARIH1, ARIH2, CUL9, and ANKIB1), RNF14, RNF144A, RNF144B, RNF19A, RNF18B, RNF216, and RNF217. All RBR E3s possess auto-inhibitory mechanisms. Structure studies reveal that the non-RBR domains occlude the catalytic cysteine on RING2, and isolates the RING2 far from the RING1-IBR, thus suppressing RBR activity (Duda et al. 2013; Trempe et al. 2013). The highly disordered linkers that connect RING1 to IBR and IBR to RING2 provide conformational flexibility, allowing structural rearrangements to expose the catalytic cysteine and fully exhibit RBR activity (Dove and Klevit 2017).

#### Nonclassical E3 ligases

Besides the classical E3 types discussed above, several atypical E3 Ub ligases have been established. UBC domain-containing BIRC6 (BRUCE/Apollon) and UBE2O have been identified as two special E2/E3 chimera, exhibiting dual E2 and E3 activities (Bartke et al. 2004; Nguyen et al. 2017; Yanagitani et al. 2017). Interestingly, several recent studies synchronously revealed a horseshoe-shaped anti-parallel dimeric architecture of BIRC6 that structurally facilitates SMAC engagement and antagonizes caspase-binding of BIRC6 (Liu et al. 2022c; Dietz et al. 2023; Hunkeler et al. 2023). Based on this, BIRC6/SMAC complex can efficiently modulate apoptosis and autophagy as a stress-induced hub (Ehrmann et al. 2022).

Notably, no human E3 enzymes targeting non-lysine residues had ever been identified until 2018. RING-Cys-relay (RCR) ligase MYCBP2/PHR1, a neuron-associated large protein that regulates axon maintenance, is a new class of E3 ligase that possesses esterification activity and intrinsic selectivity for threonine residues (Pao et al. 2018). Furthermore, the esterification activity of MYCBP2 modulates neurodevelopment and axon integrity (Mabbitt et al. 2020). Inhibition of the RCR may be a promising therapeutic strategy for mitigating neurologic diseases linked to axonal degeneration. However, whether MYCBP2 could target non-protein substrate by its high esterification activity needs further investigation.

Finally, several members of a bacterial SdiE effector family from *Legionella pneumophila* function as an E3 ligase and catalyze NAD-dependent ubiquitination on serine of multiple host GTPases, which is independent of ATP, E1, and E2 enzymes (Qiu et al. 2016). Unlike other E3s, SdiE proteins have multiple enzyme activities to complete a unique ubiquitination process. SdiE cleaves NAD, and then delivers NAD-derived ADP-ribose onto arginine 42 of Ub to generate ADP-ribosylated Ub via its mono-ADP-ribosyl transferase (mART) domain; the phosphodiesterase (PDE) domain of SdiE then converts ADP-ribosylated Ub into phosphoribosyl Ub by the cleavage of phosphodiester bond; the PDE simultaneously catalyzes a covalent ligation of phosphoribosyl Ub to substrate serine through a two-step transfer reaction (Bhogaraju et al. 2016; Akturk et al. 2018). SdiE-modified Ub can prevent the activation of host E1 and E2 and thus disturb numerous host cellular events. The DUB domain of SdiE does not disrupt its E3 activity but can cleave host Ub chains. Recently, two bacterial deubiquitinases (DupA and DupB) that harbor a catalytic PDE domain were reported to cleave phosphoribosyl-Ub conjugates catalyzed by SidE (Wan et al. 2019; Shin et al. 2020). Effector protein SidJ was also proposed to be a deubiquitinase for targeting phosphoribosyl-linked ubiquitination (Qiu et al. 2017). However, SidJ was further demonstrated to be a calmodulin-dependent enzyme that suppresses SidE E3 activity via a glutamylation reaction (Bhogaraju et al. 2019; Black et al. 2019; Gan et al. 2019). It will be extremely interesting to conduct investigations to explore SdiE-like mammalian E3 ligase in mammalian cells. However, so far, no such kind of human E3 has been identified. Given that mammalian cells can express abundant E1 and E2 proteins, a potential E1/E2-independent E3 could be easily ignored. It is almost certain that, with the continual development of research perceptions and experimental methods, more nonclassical E3 ligases should be found and reported in the near future.

### Ubiquitination linkages

A single Ub is conjugated to one or multiple residues of the substrate, resulting in monoubiquitination or multi-monoubiquitination, respectively; multiple Ub molecules can be polymerized to generate various Ub chains by a single linkage type or different linkage types, which refer to the homotypic or heterotypic polyubiquitination (Fig. 1E). Seven lysine residues (K6, K11, K27, K29, K33, K48, and K63) and the first methionine (M1) of Ub can serve as acceptor sites for next Ub, which leads to eight kinds of homotypic linkages if all the Ub molecules in a chain offer the same residue to link another one (Komander 2009; Liao et al. 2022). Mixed linkages can be formed when Ub chains having one type of linkage is extended by a different type; ubiquitination targeting multiple residues of a Ub molecule in chains will generate branched linkages (Liu et al. 2022b). Different linkages usually lead to distinct conformations, different protein recognitions, and changed functions of polyUb chains, extremely increasing the complexity and diversity of ubiquitination.

K48 and K63 linkages are probably the two best studied linkage types, while the other linkages were previously considered to be unconventional types with yet incomplete understanding but significant roles. A quantitative proteomics had revealed the relative abundances of seven individual polyUb linkages in eukaryotic cells (Xu et al. 2009). K48 and K11 linkages, which are 29% and 28% of all Ub linkages, represent two most abundant types, whereas two minimal linkage factions are K29 (3%) and K33 (3.5%); the remaining linkages K63, K6, and K27 have 16%, 11%, and 9% abundances, respectively. K48-linked Ub chains that show a compact fold structure generally target substrates for proteasomal degradation (Eddins et al. 2007). Different from K48 chains, K63-linked chains adopt an open linear conformation, controlling protein recruitment, trafficking, and activity, which plays roles in signal transduction, DNA damage repair, immune response, and other processes (Liao et al. 2022). Linear M1-linked chains have a similar structure to K63 chains and regulate NF-κB signaling (Spit et al. 2019). Interestingly, other atypical Ub linkages show distinct structural status between “open” K63 linkages and “close” K48 linkages (Liao et al. 2022). K6 linkage is proposed to have non-degradative roles, which may be involved in DNA damage response associated with E3 complex BRCA1/BARD1 or in Parkin-mediated mitochondrial homeostasis (Wu-Baer et al. 2003; Martinez et al. 2017). K11-linked chains were originally considered to be another proteolytic signal independent of K48 linkages; however, a recent study found that homotypic K11-linkages disrupts proteasomal degradation, while the heterotypic K11-linked chains are readily recognized by the proteasome in cell cycle regulation (Grice et al. 2015). As one of the noncanonical types, K27-linked ubiquitination exhibits diverse effects on DNA damage response (DDR), gene transcription, innate immune response, and T cell signaling, which has drawn more attention (Gatti et al. 2015; Li et al. 2020b, 2021; Zhou and Zhang 2022). K29-linked chains have been found to be enriched after proteasome inhibition in mammalian cells, proposing a role in regulating protein degradation; K29 linkages also modulate neuronal protection or pathogen infection (Nucifora et al. 2016; Karim et al. 2020; Sheng et al. 2020). Finally, K33-linked typical ubiquitination controls intracellular trafficking and autophagy (Heath et al. 2016; Feng et al. 2019). Although emerging insights of nonclassical Ub linkages have been established, more follow-up investigations are still needed to further elucidate the biological functions of these linkages. Several recent literature had reviewed atypical ubiquitination in detail (French et al. 2021; Squair and Virdee 2022; Zhou and Zhang 2022).

## Ubiquitination controls cellular responses to environmental stresses

Hypoxia, temperature stresses, and osmotic stress are three most common environmental stressors for cells. They could disrupt regular signaling pathways and trigger cellular responses. Ub-associated events were frequently observed in these stresses-induced responses, which will be fully discussed as follows.

### Hypoxia stress

Oxygen is fundamental for most metazoan organisms on Earth because cells utilize O_2_ to fuel aerobic respiration and maintain ATP production. In particular, vertebrates develop multiple complex systems to efficiently capture and distribute oxygen to support living cells. Cells will experience hypoxia, a state of insufficient oxygen levels, and cannot perform cellular respiration to execute normal functional events if oxygen concentrations drop below a certain extent (Schödel and Ratcliffe 2019). Once hypoxia occurs, the hypoxia signaling pathway is activated to sense O_2_ levels, initiating anaerobic glycolysis and maintaining cellular homeostasis for adaptation to oxygen starvation (Lee et al. 2020). Notably, the hypoxia response is predominantly controlled by the ubiquitination and degradation of hypoxia-inducible factor (HIF), a critical transcription factor (Muz et al. 2015).

#### HIF ubiquitination predominantly regulates hypoxia response

**Figure 2.**
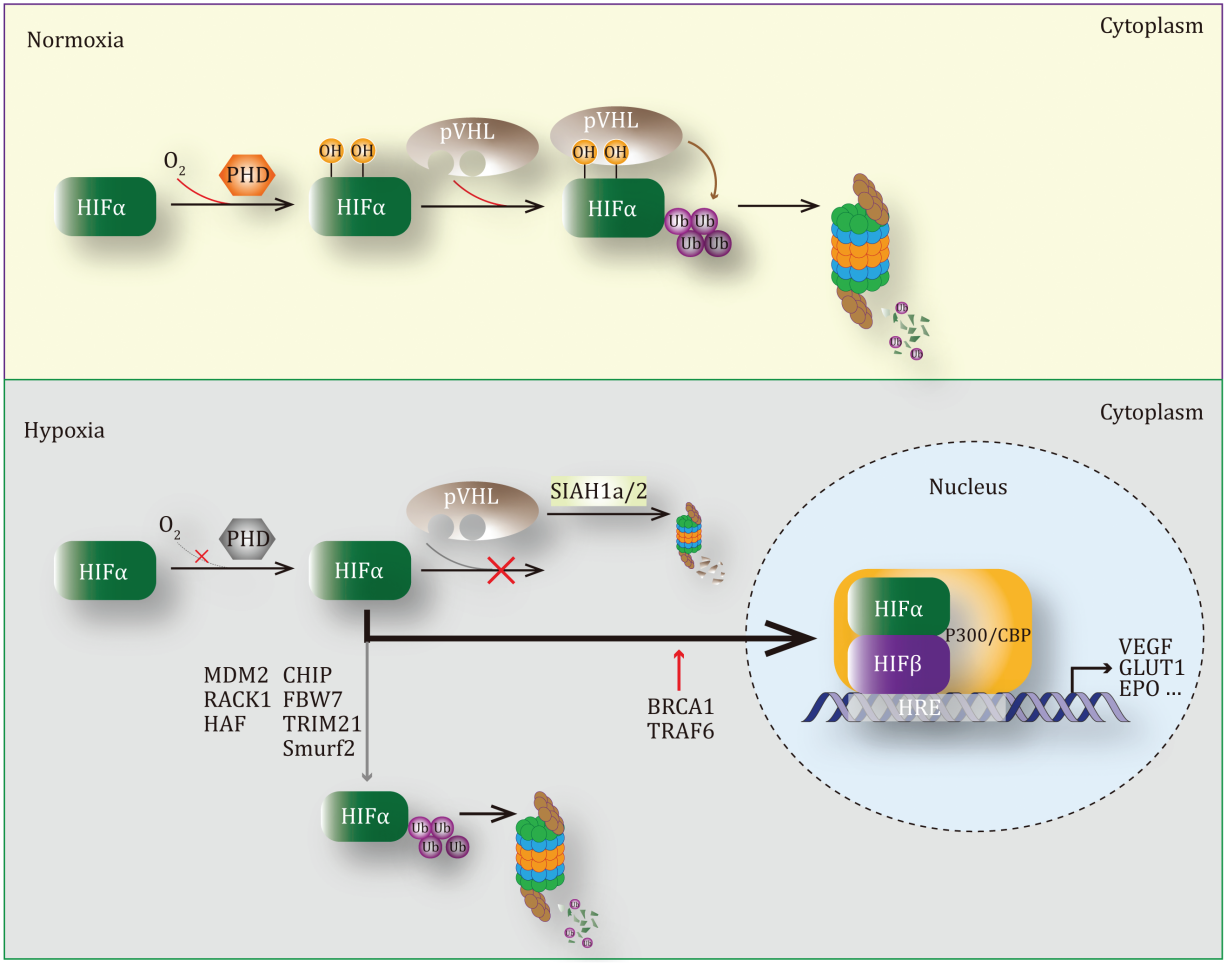
Regulation of HIF by ubiquitination. While VHL complex destroys HIFα in normoxia, some other E3 ligases can modulate HIFα accumulation under hypoxia.

HIF accumulates under hypoxia and its stability governs hypoxia response. HIF is a heterodimer that consists of a HIF-α subunit and a HIF-β subunit (Wang et al. 1995). Three HIF-α proteins (HIF-1α, HIF-2α, and HIF-3α) are Class 1 bHLH-PAS proteins, which can heterodimerize with Class 2 bHLH-PAS protein HIF-1β (ARNT) or tissue-specific expressed HIF-2β (ARNT2), via their basic bHLH-PAS domains (Wu and Rastinejad 2017; Albanese et al. 2020). In hypoxia, HIF-1α, HIF-2α, or HIF-3α interacts with HIF-1β to form HIF-1, HIF-2, or HIF-3 complex, respectively, which can bind to hypoxia response elements (HREs) and activate the transcription of various target genes, modulating cell proliferation, metabolism, migration, apoptosis, and DNA repair (Ke and Costa 2006). While β subunits are constitutively expressed in nucleus and not affected by the oxygen levels, α subunits are highly sensitive to cellular O_2_ tension and thus determine the oxygen sensitivity of HIF complexes (Lee et al. 2004). As an oxygen sensor and a critical regulator, HIF-α stability is precisely manipulated by a PTM cascade, including hydroxylation and ubiquitination (Fig. 2).

In normoxia, three prolyl hydroxylase domain (PHD) enzymes (PHD1, PHD2, and PHD3) sense and utilize O_2_ as a substrate to catalyze prolyl hydroxylation of conserved proline residues in HIF-α proteins (Pro402/564 of HIF-1α; Pro405/531 of HIF-2α; Pro492 of HIF-3α), which is the first step to destroy HIF (Bruick and McKnight 2001; Epstein et al. 2001; Ke and Costa 2006). Once hydroxylated, HIF-α proteins have more than a 1,000-fold increase in binding affinity of the von-Hippel-Lindau protein (pVHL), which functions as the recognition component of a multi-subunit RING E3 ligase (VHL complex) containing Cullin-2, Elongin-1, Elongin-2, and Ring-Box 1 (RBX1) (Hon et al. 2002; Min et al. 2002). Subsequently, the VHL complex ubiquitinates several specific lysine residues on HIF-α (K532/K538/K547 on HIF-1α, K497/503/K512 on HIF-2α) and triggers HIF-α proteasomal degradation (Maxwell et al. 1999; Ohh et al. 2000; Paltoglou and Roberts 2007). Apart from PHDs, factor inhibiting HIF (FIH), another hydroxylase, binds HIF-1α and uses molecular oxygen to catalyze asparagine hydroxylation of HIF-1α, which controls HIF-1α transcriptional activity under normoxia (Lando et al. 2002). These studies provided two different mechanisms to completely block HIF-α function in normoxia: PHD-mediated hydroxylation together with ubiquitination dramatically destabilizes HIF-α, and FIH-catalyzed hydroxylation further inhibits its activity. However, under hypoxia, PHDs show a decreased activity to catalyze HIF-α hydroxylation as available molecular oxygen is not enough, which leads to reduced interaction between HIF-α and VHL complex, therefore inhibiting HIF-α ubiquitination and promoting HIF accumulation in nucleus (Schödel and Ratcliffe 2019). Thus, controlling HIF-α abundance by oxygen levels is primarily mediated by ubiquitination and proteasomal degradation.

Although the activity of the VHL E3 complex is attenuated and HIF-α is accumulated in hypoxia, many other E3 ligases could regulate HIF-α abundance under hypoxia, which prevents the excessive accumulation of HIF-α. For example, E3 ligases MDM2, Parkin, and hypoxia-associated factor (HAF) had been shown to ubiquitinate and degrade HIF-1α irrespective of oxygen levels (Chen et al. 2003; Koh et al. 2008; Joshi et al. 2014; Liu et al. 2017). Recently, it is demonstrated that TRIM21 also physically interacts with HIF-1α and promotes its K48-linked ubiquitination and degradation (Chen et al. 2021; Zheng et al. 2021). Moreover, there are several E3 ligases that have been involved in HIF-1α ubiquitination in some diseases. Hyperglycemia had previously been proved to impair hypoxia-induced stabilization of HIF-1α protein without known mechanisms (Catrina et al. 2004). A subsequent study showed that a glycolysis byproduct methylglyoxal (MGO) can be accumulated in high-glucose conditions and promote CHIP-mediated HIF-1α ubiquitination and degradation under hypoxia (Bento et al. 2010). Furthermore, glycogen synthase kinase 3β (GSK3β) catalyzes HIF-1α phosphorylation in hypoxia, facilitating the interaction of HIF-1α and an E3 ligase FBW7 (Cassavaugh et al. 2011). FBW7 deletion promotes HIF-1α accumulation, while FBW7 overexpression ubiquitinates and degrades phosphorylated HIF-1α, thus regulating angiogenesis in cancer. Because hyperglycemia can result in GSK3β activation (Mathur et al. 2018), these studies suggested that CHIP and FBW7 may cooperate to mediate the degradation of HIF-1α in hypoxia. Additionally, a proteomics screen identified Smurf2 (SMAD-specific E3 Ub protein ligase 2) as a HIF-1α interactor that can lead to the degradation of HIF-1α in hypoxic colorectal cancer cells (Zhao and El-Deiry 2021). Therefore, the stability of accumulated HIF-1α in hypoxia can be manipulated by multiple E3 ligases, which suggests that these E3 ligases have comparable but limited capacities to degrade HIF protein.

On the other hand, some E3 ligases can stabilize HIF-1α in hypoxia. Ub ligase BRCA1 was found to interact with HIF-1α and increase HIF-1α half-life, in which the RING domain of BCRA1 is required (Kang et al. 2006). TRAF6 also associates with HIF-1α and mediates K63-linked ubiquitination of HIF-1α, which stabilizes HIF-1α independent of oxygen; but TRAF6 does not target HIF-2α (Sun et al. 2013). BRCA1 or TRAF6-induced HIF-1α stabilization is probably resulted from non-proteolytic Ub linkages conjugated on HIF-1α. Taken together, while the VHL E3 ligase complex primarily mediates HIF-1α degradation in normoxia, the regulation of HIF-1α stability in hypoxia needs ubiquitination induced by different E3 ligases, which probably maintains an appropriate accumulation of HIF-1α in hypoxia.

#### Ubiquitination of other proteins supports hypoxia response

Besides HIF-α proteins, other proteins could undergo ubiquitination in response to oxygen changes. RING E3 SIAH proteins are important regulatory proteins in the hypoxic response (Nakayama et al. 2009). When hypoxia occurs, SIAH1a/2 can target HIF-1α hydroxylases, PHD1, PHD3, and FIH, for ubiquitination and proteasomal degradation (Nakayama et al. 2004; Fukuba et al. 2008). Moreover, hypoxia increases the interaction between SIAH2 and a kinase HIPK2, a negative regulator of gene expression, leading to HIPK2 polyubiquitination and degradation (Calzado et al. 2009). Therefore, although SIAH proteins do not target HIF-1α directly, they can regulate the hypoxic response by ubiquitinating some negative regulators of hypoxia. Additionally, hypoxia deactivates Hippo signaling in a SIAH2-dependent manner. In response to low cellular O_2_ level, SIAH2 interacts with LATS2, a critical Hippo pathway component, and mediates LATS2 ubiquitination and degradation, which causes YAP nuclear translocation and tumorigenesis (Ma et al. 2015). In addition, E3 ligase Pellino-3 mediates TRAF6 ubiquitination and suppresses TRAF6 ability to ubiquitinate and stabilize HIF-1α (Siednienko et al. 2012; Yang et al. 2014). Interestingly, hypoxia can upregulate an Ub E2 UBE2M that cooperates with DJ-1/Parkin ligases to ubiquitinate and degrade another E2, UBE2F, and this event could be a negative regulatory mechanism that inhibits the growth of lung cancer cells under hypoxia (Zhou et al. 2018). In summary, ubiquitination of other proteins in hypoxia could further modulate cellular hypoxia response, via indirect ways to control HIF stability and functions.

### Temperature stresses

Sudden temperature changes can alter biochemical features of biological molecules and interfere with cellular functions. Heat shock or cold shock response can be activated when cells undergo different temperature stresses. Ubiquitination events happen in the heat/cold shock response.

#### Heat shock

**Figure 3.**
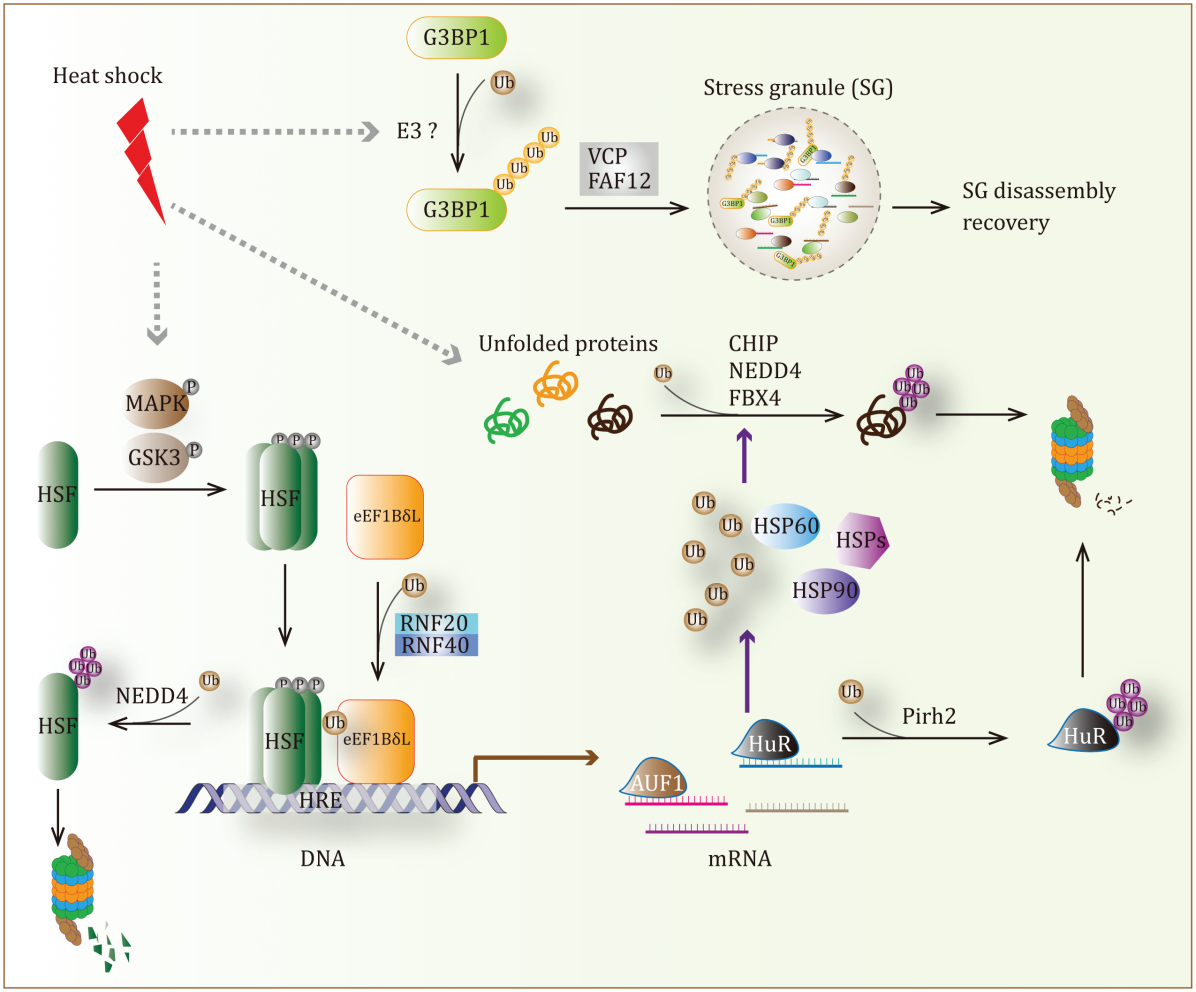
Role of ubiquitination in the heat shock response. Ubiquitination regulates the stability of HSF, the expression of heat shock-responsive genes, the degradation of unfolded proteins, and cellular recovery from heat shock.

Heat is the major temperature stressor, which usually causes the aggregation of damaged proteins. A temperature increase of just a few degrees can trigger the heat shock response, inducing the expression of a group of highly conserved heat-shock proteins (HSPs) that promote protein folding, trafficking, and complex assembly (Sonna et al. 2002; Richter et al. 2010). These HSPs are molecular chaperones and can alleviate protein aggregation. The expression of HSPs is generally determined by the activation of heat shock transcription factor 1 (HSF1). HSF1 is usually inactivated in the non-stressful cells but is activated upon heat stress. Heat shock leads to the activation of many protein kinases, including MAPK and GSK, which catalyze HSF1 phosphorylation and trimerization (Guettouche et al. 2005; Kmiecik et al. 2021). Activated trimeric HSF1 can bind to heat shock elements (HSE) DNA sequence of gene promoters and recruits transcriptional machinery to initiate genes transcription and expression, including HSPs and various other proteins. Ubiquitination has shown critical roles in regulating the heat shock response (Fig. 3).

First, Ub itself is a heat shock protein. In the *UBC* gene promoter, there are at least three HSEs with different configurations (Bond and Schlesinger 1985, 1986; Crinelli et al. 2015), which dramatically increase Ub expression and accumulation in the heat-shock cells, leading to secondary Ub stress, an intercellular stress that will be discussed later. Moreover, an increase of high molecular weight Ub conjugates is accompanied by a dramatic fall of free Ub molecules in the heat-shock cells, suggesting an elevated ubiquitination activity (Carlson et al. 1987; Parag et al. 1987). The increase of Ub and ubiquitination has been proposed to play roles in eliminating misfolded proteins and preventing excessive synthesis in heat shock. Indeed, lots of proteins are synthesized and readily accumulated upon heat shock, but newly synthesized proteins could be sensitive to Ub-dependent degradation (Medicherla and Goldberg 2008). Heat shock also triggers ubiquitination-mediated proteolysis of some RNA-binding proteins, including AUF1 and HuR, inhibiting the expression of target genes (Laroia et al. 1999; Abdelmohsen et al. 2009). Moreover, ubiquitinated nascent proteins can facilitate the recruitment of negative transcription elongation factors to gene promoters, leading to transcriptional downregulation of relevant genes (Aprile-Garcia et al. 2019). According to these studies, it is reasonable to speculate that the Ub system is a powerful strategy cells utilized to control heat-induced protein synthesis and alleviate the burden of misfolded proteins, by either direct degradation of synthesized proteins or inhibition of gene expression.

Second, several E3 ligases have shown important effects on the removal of misfolded proteins. The E3 Ub ligase CHIP is a co-chaperone of heat shock protein Hsp70/Hsp90 (Ballinger et al. 1999; Connell et al. 2001). The TPR domain of CHIP interacts with the C-terminal GPTIEEVD motif and the α-helical lid subdomain of Hsp70 (Graf et al. 2010; Zhang et al. 2015). This bipartite interaction is necessary for CHIP to ubiquitinate and degrade chaperone-bound substrates, eliminate misfolded proteins, and maintain homeostasis (Petrucelli et al. 2004; Qian et al. 2006; Soss et al. 2015). FBX4, an F-box containing E3 ligase, specifically interacts with a small HSP αB-crystallin to mediate the ubiquitination of unknown proteins (den Engelsman et al. 2003). *Drosophila* Linear Ubiquitin E3 ligase (LUBEL), an orthologue of mammalian HOIP, controls linear ubiquitination upon heat shock and promotes survival of flies (Asaoka et al. 2016). Moreover, NEDD4 was reported to be an E3 ligase that promotes ubiquitination and degradation of cytosolic misfolded proteins in the heat-shock cells (Fang et al. 2014; Kim et al. 2016). In addition, E3 ligase Pirh2 ubiquitinates HuR protein and facilitates its degradation in response to heat stress, controlling cell survival under elevated temperatures (Daks et al. 2021). These E3 ligases had shown negative roles in protein synthesis, probably providing quality control to reduce the hazard of damaged proteins in heat shock. However, some other E3 ligases have the ability to increase specific responsive proteins under heat stress. For example, RNF20/40 E3 complex monoubiquitylates Lys382 of a heat shock transcription factor eEF1BδL and promotes its accumulation, increasing the transcription of heat shock-responsive genes (In et al. 2019). Notably, CHIP could induce trimerization and transcriptional activation of HSF1 to protect against heat shock-induced cell death (Dai et al. 2003). Although ubiquitination generally limits excessive protein synthesis in heat shock, some E3 ligases may facilitate protein expression of specific response factors to execute critical functions, which needs to be explored by further research.

Finally, two recent studies from a research group have addressed a new function of ubiquitination in recovery from heat stress (Gwon et al. 2021; Maxwell et al. 2021). The total ubiquitination is dramatically increased at the beginning of heat stress and then remained at an elevated level when the stress was prolonged; but the accumulation after heat shock was temporary, rapidly returning to baseline. Specifically, heat shock leads to an increase in the ubiquitination of stress granule proteins. Surprisingly, heat stress-induced ubiquitination is not required for the assembly of stress granules, but is essential for their rapid disassembly and subsequent recovery of cellular activities following heat shock (Maxwell et al. 2021). Furthermore, the ubiquitination of the G3BP1 protein, the central protein of the stress granule induced by heat shock, was fully addressed (Gwon et al. 2021). G3BP1 undergoes K63-linked ubiquitination under heat stress within its NTF2L domain. Ubiquitinated G3BP1 next forms a complex with VCP and FAF2, which is required for stress granules disassembly during recovery from heat shock. These studies demonstrated an important role of ubiquitination of stress granule proteins in the recovery of cellular activities after heat shock; however, the E3 Ub ligases responsible for this ubiquitination remain unknown. Although a proteome analysis performed in this study did not reveal any stress-induced E3 ligases, potential E3 ligases may exhibit altered binding affinity to G3BP1 upon heat stress. Notably, TRIM25 could be a critical candidate. Yang *et al*. recently found that TRIM25 can interact with G3BP1/2 and modify them with K63-linked Ub chains (Yang et al. 2022). Whether TRIM25 is responsible for stress-induced G3BP1 ubiquitination and stress granules disassembly deserves future investigations. Knockout of *TRIM25* gene in stressed cells and exploring the changes of G3BP1 ubiquitination and granules disassembly could be a useful strategy to validate the functions of TRIM25.

#### Cold shock

Compared with that in heat shock, cellular response to cold stress in animals has attracted far less attention except in some research areas such as cell or organ preservation, cold tolerance, adaptive thermogenesis, and protein production (Fujita 1999). Generally, cold easily reduces physiological activities and leads to the alterations in supramolecular organizations. Temperature up-shift to 37°C from cold stress, as a form of heat shock, leads to dramatic cellular changes, and even cell death. However, only a limited number of genes could be upregulated during moderate hypothermia (25–33°C), and few gene expression or protein modifications has been reported to be induced by severe cold stress (below 5°C) without recovery at 37°C (Danno et al. 1997; Nishiyama et al. 1997; Sonna et al. 2002).

Adaptive thermogenesis has usually been investigated after exposing humans or rodents to cold temperature, in which several E3 Ub ligases have been found to be involved. RNF34 is a cold-regulated E3 ligase responsible for the ubiquitination of PGC-1α, a master regulator of thermogenesis, and negatively controls brown fat cell metabolism (Wei et al. 2012). Another E3 ligase Parkin is also a key protein in mitochondrial homeostasis and facilitates brown adipose tissue plasticity in response to thermogenic challenges (Cairó et al. 2019). However, it should be noted that exposing intact non-hibernating animals to cold may not lower the body temperature as expected. As shown in one study, incubating single mice at 2–3°C for 8 h only decreased the body temperatures from 36.5°C to 34.0°C, giving an average decrease of 2.5°C (Cullen and Sarge 1997). Moreover, for human, core body temperature lower than 29°C is actually life-threatening. Thus, non-hibernating animals may be not good models to study cellular responses to severe cold stress.

However, similar to bacteria or plants, ectothermic animals (fish, amphibians, reptiles, and insects), heterotherms (some species of birds, small rodents, marsupials, and bats), and some endothermic mammals (bears, hamsters, dwarf lemurs, and ground squirrels) show robust ability to tolerate cold or freezing even if the core body temperature decreases to an extremely low level (close to 0°C) in hibernation (Mohr et al. 2020). For example, the arctic ground squirrel can drop its body temperature to below 0°C and enter a hibernating status (Barnes 1989). In plants, under cold conditions (4°C), RING E3 ligase HOS1 remains highly active and mediates the cold-induced ubiquitination and degradation of a transcription factor ICE1, which controls freezing tolerance and flowering time (Dong et al. 2006; MacGregor and Penfield 2015). Mechanically, cold stress can initiate membrane rigidification, which results in a Ca^2+^ influx and MAPK cascade activations, probably causing ICE1 phosphorylation and relative conformation changes. Especially, Ser185 phosphorylation of ICE1 may promote its binding to HOS1 and subsequent degradation, because the S158A mutant of ICE1 was reported to lose the capacity to interact with HOS1 in a yeast two-hybrid assay (Cheng et al. 2020). However, no protein ubiquitination in response to such severe cold stress has been reported in animals and humans. It should be very interesting to explore whether protein PTMs happens or functions in mammalian cells upon extremely low temperature.

### Osmotic stress

**Figure 4.**
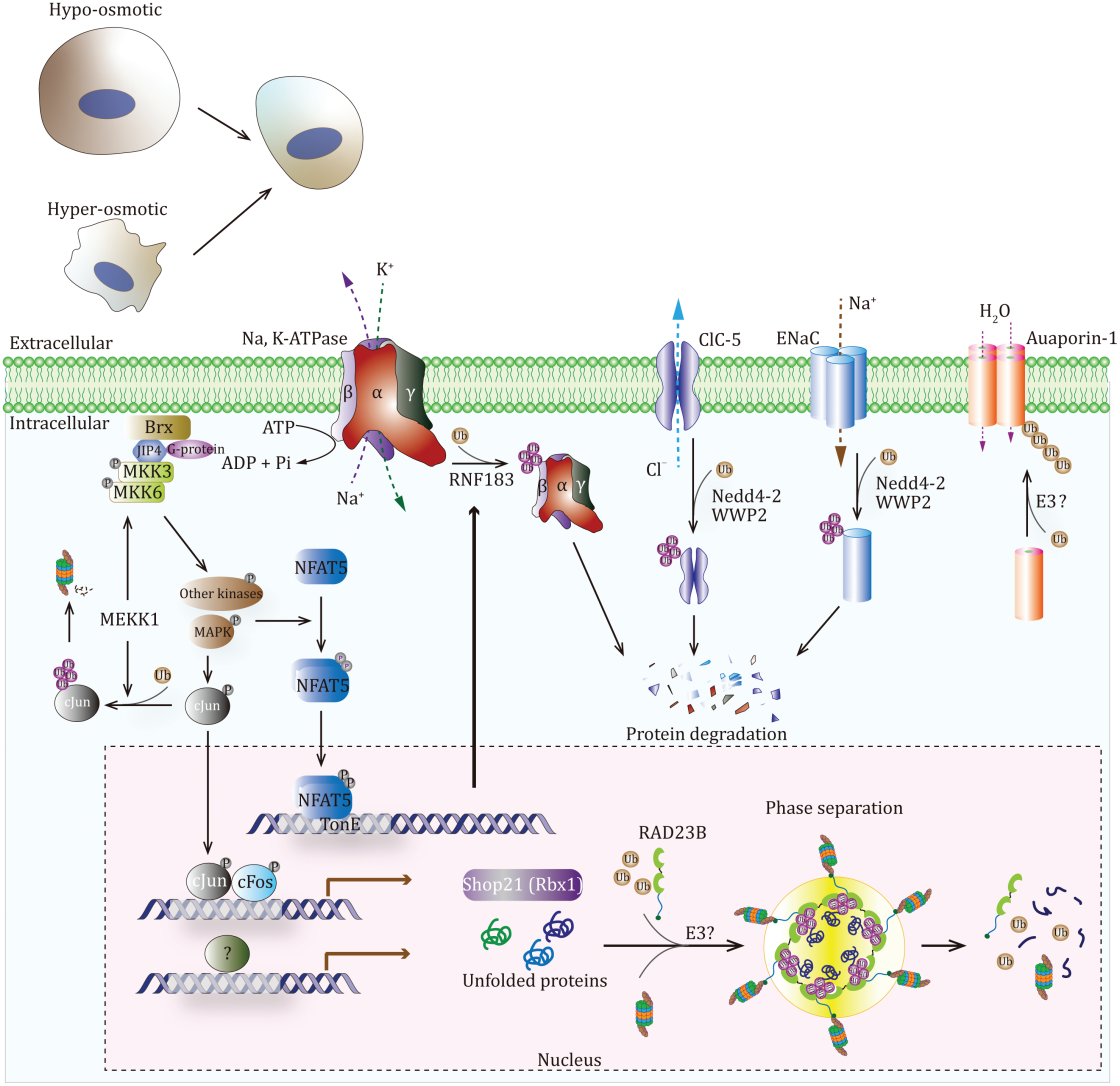
Ubiquitination regulates cellular osmolality by targeting multiple channel proteins. RNF183, Nedd4-2, WWP2, and other E3 ligases are involved in the regulation of osmotic stress.

The equilibrium osmolality is highly crucial for maintaining normal cellular functions and is tightly controlled by a balance of hydration and solute concentrations. While most cells are usually maintained in constant osmotic environment, some body cells are exposed to a dynamic osmotic environment under physiologic or pathologic conditions (Finan et al. 2011). Increasing or decreasing in the external osmolarity beyond normal range, termed hypertonic or hypotonic stress, respectively, triggers water fluxes across semipermeable cytoplasmic membrane. Some adaptive mechanisms have been developed in cells to compensate changes in extracellular osmolarity (Brocker et al. 2012). Osmotic changes trigger the alterations of cell volume, which simultaneously activates cell volume recovery mechanisms and membrane channel proteins (Sadowska et al. 2018). Channel proteins that transport water or ions across plasma membrane regulate cellular osmolarity under osmotic stress. Many Ub E3 ligases have been reported to be involved in plant osmotic stress response, but the roles of ubiquitination under osmotic stress in animal cells are still poorly studied. The roles of ubiquitination in osmotic regulation are summarized as follows (Fig. 4).

Channel proteins could be functionally modulated by ubiquitination. First, the ubiquitination and stability of aquaporin-1 (AQP-1), a water channel protein, are altered in response to hypertonic stress (Leitch et al. 2001). The hypertonic stress decreases AQP-1 ubiquitination and promotes AQP-1 accumulation, contributing to the regulation of water transport and osmolarity. Second, Ub E3 ligase Nedd4-2 binds to the proline-rich PY motifs of ENaC, an amiloride-sensitive epithelial sodium channel protein, via its WW domains, and triggers ENaC ubiquitination and degradation and regulates salt and water balance (Staub et al. 1996, 1997; Kamynina et al. 2001). WWP2, another member of the Nedd4 family, also targets ENaC (McDonald et al. 2002). Moreover, Nedd4-2 and WWP2 may have a role in regulating a chloride channel ClC-5 via the interaction between WW domains and PY motifs (Schwake et al. 2001; Hryciw et al. 2004). Thus, the Ub system regulates osmosis via ubiquitinating some channel proteins and modulating their capacities to transport water or ions.

RNF183 may be the most important E3 ligase in osmosis regulation. The mRNA of RNF183 was reported to be specifically expressed in human and mouse kidney (Kaneko et al. 2016). In particular, by using *RNF183-GFP* knock-in mice, a recent study shows that RNF183 predominantly locates in the renal medullary collecting ducts and colocalizes with water channel aquaporin-2 (Maeoka et al. 2019a). Interestingly, the renal medulla of kidney is the only tissue that is continuously under a hypertonic environment (Bankir et al. 1989). These studies suggest that RNF183 may be involved in cellular hypertonic response. Indeed, the promoter of gene *RNF183* is bound directly by nuclear factor of activated T cells 5 (NFAT5), a transcription factor that drives gene expression for adaption to the hypertonic stress (Neuhofer 2010; Maeoka et al. 2019b). Moreover, hypertonic stress specifically promotes RNF183 expression, which is consistent with the activation of NFAT5; on the other hand, NFAT5 knockdown decreases the expression level of RNF183. Consistently, furosemide, a loop diuretic that has been proved to efficiently suppress NFAT5 level (Sheen et al. 2009), can dramatically decrease RNF183 expression (Maeoka et al. 2019a). Thus, NFAT5 mediate the expression and increase of RNF183 in response to hypertonic stress. Furthermore, RNF183 has been shown to be crucial to inhibit caspase-3 activation and maintain cell viability under hypertonic stress (Maeoka et al. 2019b). Together, RNF183 is involved in cellular response to osmotic stress.

Moreover, Na, K-ATPase, an ion transporter responsible for active transport of Na^+^ and K^+^ across cell membrane, has been recently identified as a specific target of RNF183 (Okamoto et al. 2020). As a ubiquitously expressed transmembrane complex, Na, K-ATPase contains a large catalytic α1 subunit and a small β1 subunit, maintains ionic homeostasis in the cytoplasm, and thus contributes to cell volume regulation and osmotic adaption (Kaplan 2002; Matchkov and Krivoi 2016). Although RNF183 binds both α1 and β1 subunits, RNF183 only ubiquitinates the β1 subunit, leading to translocation and degradation of both α1 and β1 subunits in lysosomes (Okamoto et al. 2020). Taken together, hypertonicity-responsive E3 ligase RNF183 functions for hypotonic environment adaptation through modulating Na, K-ATPase activity, and maintaining ionic homeostasis.

Additionally, some specific E3 ligases may also have roles in regulating osmotic stress. under hypertonic stress, a major transcription factor c-Jun undergoes ubiquitination-dependent degradation by the PHD/RING finger domain of MEKK1, which exhibits E3 ligase activity toward c-Jun and kinase activity toward several MKKs (McCabe and Burrell 2001; Xia et al. 2007). Shop21, a homolog of Ub ligase Rbx1, had been reported to be increased upon hyperosmotic stress in salmon (Pan et al. 2002). Although transferring salmon to seawater leads to Shop21 accumulation, the role of Shop21 under hyperosmotic stress remains unclear. Rapid correction of chronic hyponatremia can result in intense osmotic stress in brain cells, which induces ubiquitination and insoluble aggregation of unfolded proteins and contributes to demyelination syndrome development, suggesting osmotic stress may be a potent protein aggregation stimuli in mammalian brains (Gankam-Kengne et al. 2017). However, the functions of this ubiquitination in protein aggregation were still unexplored. Interestingly, a recent study demonstrated that hyperosmotic stress can trigger ubiquitination-dependent liquid–liquid phase separation (LLPS) of proteasomes in the nucleus (Yasuda et al. 2020). Following hyperosmotic stress, some ribosomal proteins that failed to properly fold can undergo ubiquitination, and RAD23B, a shuttle factor that has ubiquitin-associated domains, further drives LLPS of ubiquitinated ribosomal proteins and nuclear proteasomes, leading to proteasomal removal of misfolded ribosomal proteins. Notably, the mechanism by which the proteasome-containing structures induced by hyperosmotic stress were prominently formed in the nucleoplasm, but not the cytoplasm, remain unclear. Potential E3 ligases responsible for LLPS of ubiquitinated proteins and proteasomes also need to be elucidated in the future. In summary, these available, although limited, studies have highlighted the critical roles of ubiquitination in cellular responses to osmotic stress.

## Ubiquitination regulates responses to intercellular stresses

Besides triggering adaptive responses to recover from disruption, these exogenous stresses we have discussed above could result in multiple intercellular stresses if the initial stressful situation is prolonged. Hypoxia or heat shock induces several intercellular stresses, including DNA strand breaks, generation of reactive oxygen species (ROS), and endoplasmic reticulum (ER) stress (Møller et al. 2001; Bettaieb and Averill-Bates 2015; Wang et al. 2019; Akman et al. 2021). Heat shock also leads to Ub accumulation, also named Ub stress. Moreover, many studies had demonstrated that hyperosmolarity elevates ROS and causes ER stress (Burgos et al. 2019; Dai et al. 2019). In addition, there are many cross-talks between different intercellular stressors. Similar to environmental stresses, intercellular stresses, such as DNA damage stress, Ub stress, ROS stress, and ER stress, can initiate further cellular responses that are modulated by the ubiquitination system.

### DNA damage stress

**Figure 5.**
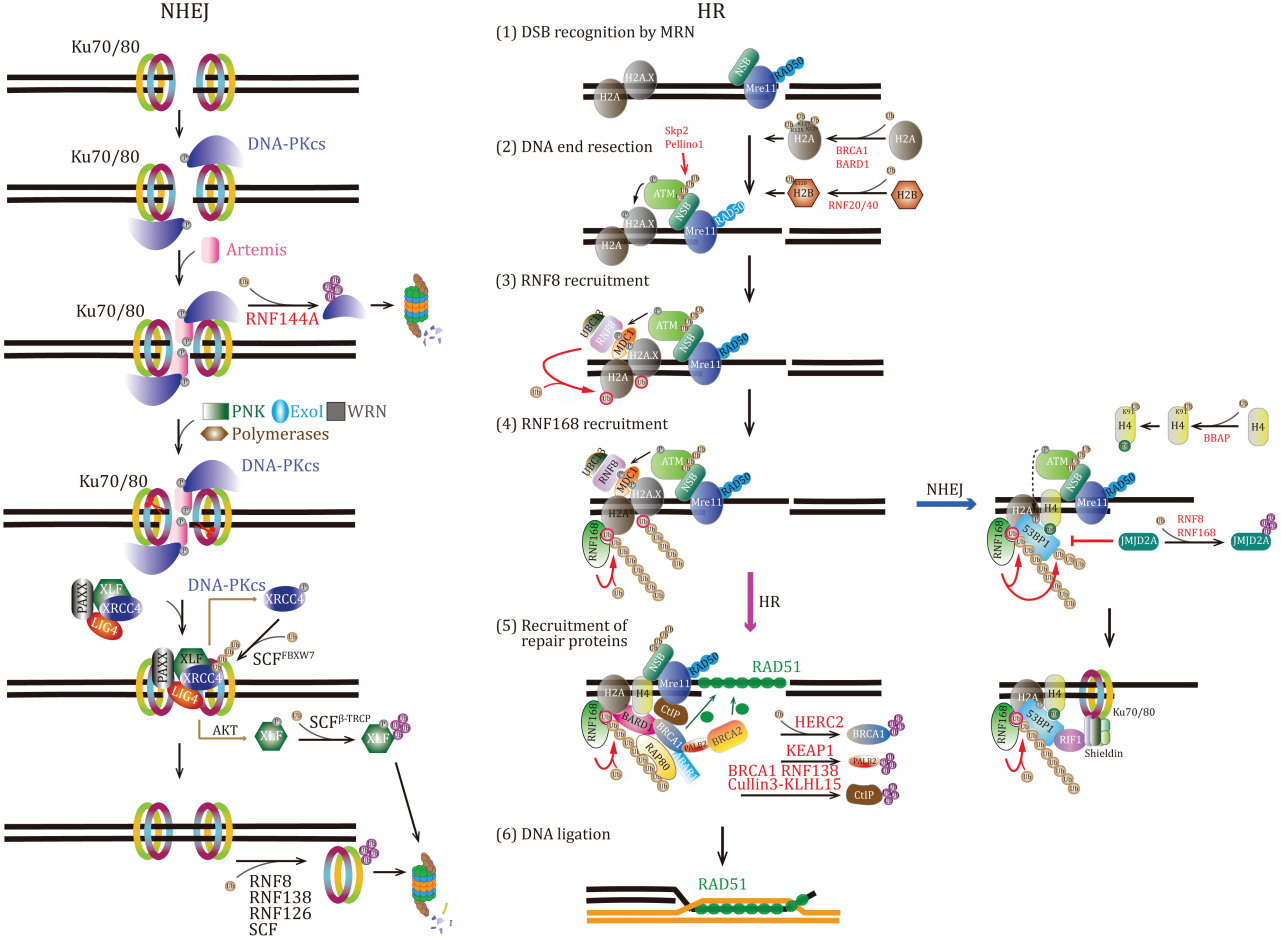
Ubiquitination controls DNA damage repair responses. Some E3 ligases have shown important functions in NHEJ pathway (left) or HR repair (right).

Large amounts of environmental or physical stressors can generate thousands of DNA lesions per cell per day, which causes many types of damages, such as DNA-protein crosslinks, base damage (BD), single-strand breaks (SSB), and double-strand breaks (DSB) (Table 1) (Jackson and Bartek 2009; Barghouth et al. 2019; Hindi et al. 2021). Genomic instability causes devastating effects that could result in various diseases and threaten the viability of cells or organism (Lord and Ashworth 2012). To maintain genomic integrity, cell have evolved complex and accurate repair mechanisms that counteract DNA damages, termed DNA damage response (DDR). DDR is tightly regulated by multiple PTMs, among which ubiquitination plays a central role and coordinates other PTMs (Mattiroli and Penengo 2021). Given the fact that DSB is the major and most dangerous DNA lesion (Podhorecka et al. 2010), this review mainly focused on ubiquitination-associated signaling cascades in DSB response. Once DSBs occur, these broken DNA sites can be recognized quickly by either MRN protein complex or Ku heterodimer, which subsequently guides repair signals via homologous recombination (HR) or non-homologous end-joining (NHEJ), respectively (Fig. 5).

**Table 1.** Reasons and repair mechanisms of different DNA lesions.

Damaging agents	Replication errors	Ultraviolet light Chemicals	Ultraviolet light Alkylating agents	Oxygen radicals (ROS) Chemotherapeutics X-rays	Ionizing radiation Chemotherapeutics X-rays	
**DNA lesions**	Base mismatch Deletions Insertions	Bulky adducts Intrastrand Crosslinks	Small adducts Reverse/Release	Single-strand breaks Abasic sites Base deamination 8-Oxoguanine lesions	Double-strand breaks Single-strand breaks Inter/intra-strand crosslinks	
**Repair pathways**	Mismatch mediated repair (MMR)	Nucleotide excision repair (NER)	Direct damage reversal	Base excision repair (BER)	Non-homologous end-joining (NHEJ)	Homologous recombination (HR)

#### NHEJ repair response

Ku-mediated NHEJ is a faster but more error-prone repair pathway than HR. Although NHEJ may create base deletions/insertions at damage sites, it plays important roles in safeguarding genomic integrity upon acute repair at any stages of the cell cycle. NHEJ is usually initiated by K70/80 protein heterodimer and glues two broken DNA ends at a DSB site, regardless of the original DNA sequence. While Ku70/80 heterodimer has less affinity to single-strand DNA ends and circular DNA, it shows a very high affinity to double-strand DNA ends and thus recognizes DSB sites within 5 s after DNA damage (Fell and Schild-Poulter 2015). Once being recruited to DSB site, Ku70/80 encircles and stabilizes the two ends of the DSB site. Next, Ku70/80 dimer works as a scaffold to recruit a transducer kinase DNA-PK_CS_, inducing its autophosphorylation and activation. Active DNA-PK_CS_ complex further attracts multiple factors, including Artemis, polymerases, kinases, and nucleases, to remove damaged DNA bases and finally form compatible sites for the ligation complex (Ligase IV, XRCC4, XLF, and PAXX) that directly heals the DNA break (Hammel et al. 2011; Ochi et al. 2015).

While the NHEJ process maybe not require ubiquitination as described above, it is regulated by ubiquitination. First, ubiquitination of Ku protein facilitates its release from the ligated sites. RNF8, RNF138, and a Fbxl12-containing SCF complex mediate K48-linked ubiquitination and degradation of Ku80 protein, which promotes Ku removal and NHEJ repair (Feng and Chen 2012; Postow and Funabiki 2013; Ismail et al. 2015). Another E3 ligase RNF126 was also found to promote Ku80 ubiquitination and dissociation from DSB sites (Ishida et al. 2017). Moreover, RING E3 RNF144A had been identified to physically interact with DNA-PK_CS_ and trigger DNA-PK_CS_ ubiquitination and degradation, which promotes apoptosis upon DNA damage (Ho et al. 2014). Additionally, components of the ligation complex could be targeted by ubiquitination. XLF undergoes Akt-mediated phosphorylation at Thr181, which leads to its dissociation from the ligation complex and cytoplasmic retention; in the cytoplasm, an E3 ligase complex SCF^β-TRCP^ ubiquitinates and degrades phosphorylated XLF, impairing NHEJ and promoting tumorigenesis (Liu et al. 2015). Another component XRCC4 was previously demonstrated to undergo monoubiquitination, which is increased in etoposide-induced DNA damage and may play roles in stabilizing DNA ligase IV (Foster et al. 2006). XRCC4 phosphorylation at S325/326 by DNA-PK_CS_ can promote its interaction with FBXW7, which leads to its K63-linked ubiquitination at Lys296 catalyzed by SCF^FBXW7^ E3 complex (Zhang et al. 2016). XRCC4 polyubiquitination may work as an extended scaffold to recruit other NHEJ factors to facilitate NHEJ complex formation and promote NHEJ repair ability. Therefore, ubiquitination could be an efficient tool to modulate the NHEJ process.

#### HR repair response

HR primarily works in the S and G^2^ phases of the cell cycle, and has high accuracy to repair DSBs by using a sister chromatid as the homologous template. HR repair owns a ubiquitination-dependent process that is more complicated than NHEJ repair.

##### DSB recognition by MRN complex

The MRN complex, comprising MRE11, RAD50, and NBS1 proteins, is an important DSB sensor, and has intrinsic DNA-binding activity and nucleolytic activities (Paull and Gellert 1998; de Jager et al. 2001). MRN searches and binds to broken DNA DSB ends, leading to Ku70/80 removal and DNA end resection that generates a long single-strand DNA (ssDNA) for searching homologous sequences. Next, an E3 ligase Skp2 is recruited by MRN and catalyzes NBS1 ubiquitination in a K63-linkage-dependent manner to attract kinase ATM to the DNA damage site, where ATM induces the phosphorylation on serine 139 of histone H2A.X and promotes γ-H2A.X foci formation (Falck et al. 2005; Lee and Paull 2005; Wu et al. 2012). In addition to Spk2, another E3 ligase Pellino-1 also ubiquitinate NBS1, controlling ATM recruitment to DSBs (Ha et al. 2019). Thus, K63-linked ubiquitination contributes to the initiation of HR repair.

##### RNF8 recruitment

MDC1 directly binds to and protects γ-H2A.X via the BRCT domain to amplify DNA damage signals, which leads to MDC1 phosphorylation that is required for the recruitment of RNF8, a very critical E3 Ub ligase (Stucki et al. 2005; Lou et al. 2006; Kolas et al. 2007). Upon binding phosphorylated MDC1 rapidly through the FHA domain, RNF8 can ubiquitylate histone H2A and H2A.X via cooperating with E2 enzyme UBC13 at damage sites, which is very critical for DDR foci formations of various proteins and promotes repair (Huen et al. 2007; Kolas et al. 2007; Mailand et al. 2007; Wang and Elledge 2007). Once the initial monoubiquitination is catalyzed by RNF8, another E3 ligase RNF168 will subsequently participate to transmit the repair signal (Doil et al. 2009).

##### The recruitment of RNF168

Mutations of *RNF168* lead to RIDDLE syndrome, an immunodeficiency and radiosensitivity disorder (Stewart et al. 2009; Bohgaki et al. 2011; Pietrucha et al. 2017). The RING domain of RNF168 is required for the accumulation of downstream 53BP1 and BRCA1, but not for RNF168 loading at DSBs, indicating RNF168-mediated ubiquitination is dispensable for RNF168 recruitment at DSBs. Instead, its two Ub-binding motifs MIU1 and MIU2 are responsible for RNF168 itself accumulation at damage sites through physical interaction with RNF8-mediated ubiquitylated H2A (Doil et al. 2009; Stewart et al. 2009). Interestingly, this widely accepted model is not supported by an *in vitro* assay showing that RNF8 is inactive toward nucleosomal H2A, but shows a high activity to ubiquitinate free H2A (Mattiroli et al. 2012). The study thus proposes a different model by which RNF8 mediates the ubiquitylation of other non-nucleosomal proteins, providing the docking sites for RNF168 recruitment. Recently, L3MBTL2 and KMT5A had been proposed to be such two non-nucleosomal proteins that are ubiquitinated by RNF8 and facilitate RNF168 recruitment to the DSB sites (Nowsheen et al. 2018; Lu et al. 2021). However, given that the biggest difference between nucleosomal H2A and isolated H2A is the presence or absence of the intact DNA, we here propose a novel hypothesis that RNF8 may directly recognize H2A molecules exposed at DNA damage sites, but cannot catalyze the H2A covered with intact DNA strands in the nucleosomes. Thus, our analysis supports the first model that RNF8-dependent H2A monoubiquitination is the first step and represents the docking sites for the following recruitment of RNF168 to the DNA damage sites. Further research is needed to verify the proposed selectivity of RNF8 toward free H2A molecule without DNA coverage. Altogether, RNF168 recruitment to DSBs is primarily mediated by RNF8-dependent ubiquitination of H2A.

Additionally, RNF8 and HUWE1 may also catalyze K63-linked polyubiquitination of histone H1 to recruit RNF168 to DSBs (Thorslund et al. 2015; Mandemaker et al. 2017). However, the removal of histone H1 from the entire γH2A.X domain has been observed in a genome-wide analysis of multiple chromatin features at DSBs using ChIP-seq (Clouaire et al. 2018). So, the hypothesized role for RNF168 docking of H1 ubiquitination remains controversial and needs further mechanistic analysis. Upon recruitment to the initial ubiquitinated H2A, RNF168 can ubiquitylate Lys13 or Lys15 of H2A/H2A.X to generate K63-linked polyubiquitin conjugates to amplify Ub signal, which serves as a binding platform that allows accumulation of proper repair factors at DNA damage sites (Mattiroli et al. 2012; Horn et al. 2019). Moreover, RNF168 also catalyzes noncanonical K27-linked H2As ubiquitination to signal DNA damage by assembling DDR foci at DSBs (Gatti et al. 2015). Although RNF168-dependent both K27- and K63-linked Ub chains on histone H2As are required to recruit proper repair factors to drive DDR, the interplay between them remains unclear.

##### DNA ligation

The effector proteins that are recruited to DDR foci determine the type of DNA repair pathway. The Ub-interacting motifs (UIM) of RAD80 protein binds RNF8/RNF168-induced Ub conjugates and facilitates RAD80/ABRA1/BRCA1/BARD1 complex assembly at the sites of DNA damage, activating HR repair (Kim et al. 2007; Sobhian et al. 2007; Wang et al. 2007). BARD1 (the partner protein of BRCA1) binds damage-containing nucleosomes bivalently by recognizing ubiquitinated Lys15 of H2A (H2AK15ub) via its BUDR motif, and unmethylated Lys20 of histone 4 (H4K20) by its ankyrin repeat domains (Becker et al. 2021). Moreover, BRCA1 binds to BRCA2 through PALB2 to assemble a complex that enhances recombinase RAD51 loading at DSBs and thus promotes HR repair (Sy et al. 2009; Zhang et al. 2009). Recently, BRCA1/BARD1 complex is also demonstrated to directly interact with RAD51 and increase the recombinase activity of RAD51 (Zhao et al. 2017). On the other side, by simultaneous recognition of ubiquitylated H2AK15 and methylated Lys20 of histone 4 (H4K20me) via its ubiquitylation-dependent recruitment (UDR) motif and the tandem Tudor domain, respectively, 53BP1 can be recruited to the damage sites, which will attract Ku70/80 complex and initiate NHEJ-mediated repair (Fradet-Turcotte et al. 2013; Wilson et al. 2016). These studies thus suggested that ubiquitination triggers the recruitment of repair proteins, and methylation determines which proteins could be recruited to initiate the corresponding repair pathway.

#### Ubiquitination modulates the switch between HR and NHEJ

The balance between HR and NHEJ can be elegantly maintained by several Ub-associated mechanisms. First, as mentioned above, RNF168-induced H2AK15ub is the common base for recruiting BRCA1/BARD1 or 53BP1 to damage sites, but methylated H4K20 prefers 53BP1 oligomers while unmethylated H4K20 chooses BRCA1/BARD1, which switches DNA repair pathways (Fradet-Turcotte et al. 2013; Becker et al. 2021). Second, a novel phosphorylation of Ub molecule at Thr12 (pUbT12) regulates the function of H2AK15ub (Walser et al. 2020). The pUbT12 of RNF168-induced H2AK15ub accumulates in DDR foci upon DNA damage and specifically suppresses 53BP1 recruitment by impeding the recognition of 53BP1 UDR domain, but is still permissive to HR mediators BRCA1/BARD1, RAD51, and RNF169, inhibiting NHEJ but promoting HR repair. Although the pUbT12-positive foci are induced in a DDR kinases-dependent manner, the kinase that directly phosphorylates Ub is still a subject of investigation.

Moreover, many E3 ligases show critical impacts on the switch between HR and NHEJ. On one hand, some E3s inhibit HR repair. A HECT-type E3 HERC2 ubiquitinates BRCA1, which promotes the degradation of BARD1-uncoupled BRCA1 and prevents the HR pathway (Wu et al. 2010). Keap1 ubiquitinates BRCA1-interacted site on PALB2 and thus suppresses BRCA1–PALB2 interaction and HR (Orthwein et al. 2015). Moreover, BRCA1, RNF138, and Cullin3-KLHL15 can mediate the ubiquitination of CtIP, the binding partner of BRCA1, to regulate HR repair (Yu et al. 2006; Schmidt et al. 2015; Ferretti et al. 2016). On the other hand, some E3s target 53BP1 to control NHEJ repair. TIRR (Tudor interacting repair regulator) directly interacts with 53BP1 and impedes 53BP1 recruitment to H4K20me, restricting NHEJ reaction (Drané et al. 2017). Interestingly, RNF168 is reported to ubiquitylate 53BP1 and the ubiquitination of 53BP1 contributes its recruitment to DSBs (Bohgaki et al. 2013). Additionally, Tudor domain-containing JMJD2A can antagonize 53BP1 recruitment to DSB sites by binding to demethylated H4K20, but RNF8 and RNF168 trigger JMJD2A ubiquitination and degradation to facilitate 53BP1 foci formation (Mallette et al. 2012). BRCA1 prevents the accumulation of 53BP1/RIF1 complex and inhibits NHEJ by recruiting CtIP protein at DSB sites (Escribano-Díaz et al. 2013). Therefore, E3 ligases can work as effective switch regulators in DNA repair pathways.

Besides the ubiquitination discussed above, Ub conjugated to other sites of histone H2A or to other histone proteins beyond H2A further increases the complexity of DDR regulation. While E3 complex PRC1-catalyzed H2AK118/119ub is involved in transcriptional repression near the break site (Ui et al. 2015; Uckelmann and Sixma 2017), BRCA1/BARD1 complex can trigger ubiquitination at K125/127/129 of H2A, which facilitates DNA end resection in DNA repair (Kalb et al. 2014; Densham et al. 2016). E3 ligases RNF20/40 can promote monoubiquitination at Lys120 of histone H2B (H2BK120ub) to promote chromatin opening and accumulate DNA repair proteins at DSBs (Fierz et al. 2011; Moyal et al. 2011; Nakamura et al. 2011; So et al. 2019). Interestingly, H2BK120 could undergo a switch from ubiquitination to acetylation, and H2BK120ub displays a progressive loss, upon DSB induction (Clouaire et al. 2018). The different PTMs on H2BK120 may exhibit distinct functions at the different stages of DDR. Similar to H2BK120ub, H2B Lys34 ubiquitination (H2BK34ub) can also induce nucleosome distortion for Dot1L binding and activation, stimulating Dot1L-mediated H3K79 methylation (Ai et al. 2022). Additionally, histone H3 and H4 ubiquitination is mediated by E3 ligase complex CUL4-DDB-ROC1 and participates in cellular response to DNA damage (Wang et al. 2006). And, E3 ligase BBAP can monoubiquitylate H4K91, which promotes H4K20me generation and 53BP1 foci formation at the DNA damage sites (Yan et al. 2009). Based on these studies, it is reasonable to speculate that ubiquitination happening on histone proteins usually have impacts on DDR. Although these ubiquitination events cannot cause protein degradation, they effectively modify DDR.

Notably, some E3 ligases can modulate different steps of DDR via catalyzing proteolytic ubiquitination. Two SUMO E3 PIAS1 and PIAS4 mediate MDC1 SUMOylation, which further recruits a SUMO-targeted E3 ligase RNF4 to ubiquitinate and degrade SUMOylated MDC1 in a K48-linkage-dependent manner, promoting MDC foci turnover at damage sites and thus maintaining a proper DDR level (Shi et al. 2008; Galanty et al. 2012; Luo et al. 2012; Yin et al. 2012). TRIP12 and UBR5, two HECT E3s, can induce RNF168 polyubiquitination, preventing the massive accumulation of RNF168 and Ub conjugates at DSBs (Gudjonsson et al. 2012). Moreover, E3 ligase PRP19, RFWD3, or RNF4 had been found to regulate DNA damage-induced ubiquitination and the stability of the RPA complex, an essential regulator in genome maintenance (Galanty et al. 2012; Maréchal et al. 2014; Elia et al. 2015). Protein CLASPIN also plays an important role in DDR activation and undergoes the ubiquitination-dependent degradation mediated by APC/Cdh1 or βTrCP-SCF (Mailand et al. 2006; Peschiaroli et al. 2006; Bassermann et al. 2008). Recently, TRIM21 was also demonstrated to target CLASPIN for K63-linked ubiquitination (Zhu et al. 2022). Although it is non-proteolytic, it counteracts K6-linked ubiquitination and the chromatin loading of CLASPIN, proposing a regulatory role of TRIM21-mediated ubiquitination on DNA repair.

In summary, ubiquitination regulates the initiation, progression, and termination of DDR, and controls the switch between different types of cellular responses, which makes DDR an elegant and accurate machinery.

### Ub stress

As one of the most abundant proteins in eukaryotic cells, Ub concentration has been estimated to be ~500 pmol/mg total cell protein or ~85 μmol/L per cell (Kaiser et al. 2011). In cells, Ub molecule undergoes a dynamic equilibrium between “conjugated” Ub covalently attached to substrates or “free” Ub available for ubiquitination reactions (Park and Ryu 2014). In various cell lines, ~23% of Ub is presented as free form, ~76% as conjugated status (65% for monoubiquitinated substrates and 11% for polyUb chains); In contrast, in mammalian brains, 60%–80% of Ub exists as free Ub monomer, which may facilitate the rapid response of neurons upon stresses (Kaiser et al. 2011). Maintenance of free Ub levels can balance a wide range of cellular processes, and cells develop different regulatory mechanisms to control cellular Ub homeostasis (Kimura and Tanaka 2010; Chen et al. 2011). Many environmental, genetic, or pathological factors could lead to the alteration of Ub homeostasis, which can be termed as Ub stress (Hanna et al. 2007; Peng et al. 2017). According to the changes in cellular Ub level, Ub stress can be classified as Ub^+^ stress or Ub^−^ stress.

Heat shock, DNA damage reagents, Ub overexpression, or prolonged proteasomal inhibition could increase Ub concentrations and lead to Ub^+^ stress (Bond and Schlesinger 1985; Fornace et al. 1989; Kaiser et al. 2011). Our group previously had demonstrated that Ub^+^ stress can trigger autophagy in a p62-dependent manner (Peng et al. 2017). Specifically, Ub^+^ stress promotes UBE2D2/3-mediated ubiquitylation on multiple sites (including K420 in the UBA domain) of p62, which disrupts UBA domain-mediated p62 dimerization and thus allows p62 oligomerization. This conformational change of p62 favors polyubiquitinated cargoes for selective autophagy (Liu et al. 2014; Peng et al. 2017; Pohl and Dikic 2019). Interestingly, UBE2D2/3-catalyzed p62 ubiquitylation under Ub^+^ stress does not require a special E3 ligase. In another study, the K420 residue of p62 was also reported to be ubiquitinated, which was mediated by an E3 complex Keap1/Cullin3 (Lee et al. 2017). Keap1/Cullin3 enhances autophagic activity by targeting dimeric p62, which thus increases p62 oligomerization and its subsequent degradation. Although these studies consistently support the conclusion that ubiquitination of a conserved lysine K420 can lead to p62 oligomerization, they have different opinions on the requirement of E3 ligase for K420 ubiquitination. This discrepancy may be explained by different Ub stress states. Ub^+^ stress may preferentially induce E3-independent K420 ubiquitination of p62. Recently, USP8 suppresses autophagy by directly deubiquitinating p62 principally at K420 (Peng et al. 2020). In addition, E3 ligase TRIM72 was found to ubiquitinate and degrade an alternative short form of p62, thereby modulating selective autophagy (Wang et al. 2022a). Further investigation is needed to explore whether Keap1/Cullin3 or TRIM72 may play roles in Ub stress response. Together, Ub^+^ stress leads to autophagic events.

On the other side, Ub depletion by cycloheximide treatment or deletion of polyubiquitin genes (*UBI4* in yeast, *UBB*/*UBC* in mammals) could cause Ub^-^ stress, which leads to decreased cellular functions, reduced viability or impaired cellular resistance to stress conditions (Park and Ryu 2014). Moreover, DUBs show important roles in regulating Ub homeostasis. Deletion of some DUB genes in mice also leads to a decreased level of free Ub and triggers neurological abnormalities and severe symptoms (Walters et al. 2008). Therefore, Ub depletion can inhibit the overall function of the Ub system, which may endanger various biological processes and cell survival. For such kind of lethal stress, mammalian cells may have few adaptive responses. However, as mentioned above, Ub is encoded by four different genes in the human genome, which may be an evolutionarily acquired safe strategy for cells to ensure adequate Ub supply and avoid the situations of Ub deficiency in the best possible way.

### ER stress

**Figure 6.**
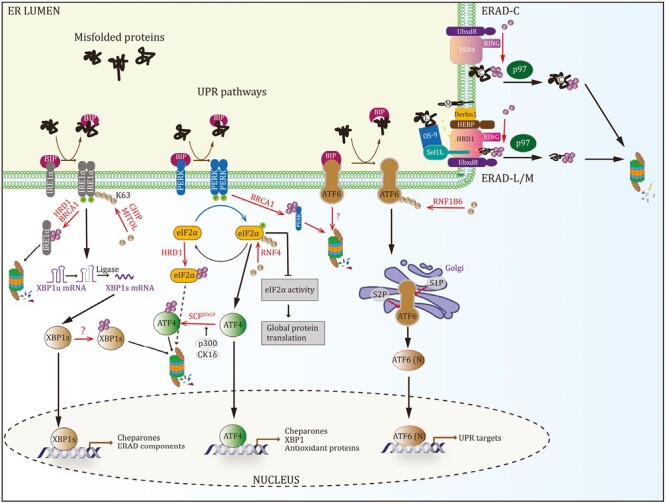
Ubiquitination regulates ER stress by modulating ERAD and UPR pathways. On one hand, misfolded proteins accumulated in the ER trigger UPR pathway. Ubiquitination can regulate protein-folding capacity of ER by targeting UPR sensors or related transcription factors. On the other hand, ERAD promotes the degradation of unfolded proteins to alleviate ER stress.

As an important intracellular organelle, ER predominantly mediates folding, translocation, and posttranslational processing of secretory and membrane proteins. Many genetic and environmental insults, such as hypoxia, heat shock, or glucose starvation, could perturb ER function, which results in accumulation of misfolded or unfolded proteins in the ER, called ER stress (Sano and Reed 2013). Once ER stress happens, ER-associated protein degradation (ERAD) could be activated to eliminate unfolded proteins, and the unfolded protein response (UPR), a highly conserved corrective mechanism, is also triggered to rapidly improve protein-folding capacity to restore ER homeostasis (Hwang and Qi 2018). However, if the ER stress cannot be alleviated, prolonged ER stress often causes cell death. Ubiquitination has shown critical roles in regulating both ERAD and UPR (Fig. 6), which facilitates ER homeostasis and cell survival.

ERAD has three different pathways, containing ERAD-L, ERAD-M, and ERAD-C, which degrades misfolded substrates in the ER lumen, within the ER membrane, and on the cytoplasm, respectively (Wu and Rapoport 2018). E3 Ub ligases are required for these ERAD pathways. In yeast, a RING E3 ligase Hrd1-containing protein complexes mainly mediate ERAD-L and ERAD-M pathways to remove misfolded proteins; another E3 ligase Doa10 is responsible for ERAD-C. In addition, a Ub ligase complex, consisting of Asi1, 2, and 3, can degrade misfolded proteins at the inner nuclear membrane (Foresti et al. 2014; Khmelinskii et al. 2014). Mammalian cells possess a highly consistent ERAD machinery, which is mediated by homologous E3 ligases SEL1L-HRD1, TEB4, and gp78/CHIP/RMA1 complex (Lopata et al. 2020). After being polyubiquitinated, ERAD-L and ERAD-M substrates can be pulled out from the membrane by p97 (Cdc48 in yeast) and undergo proteasome-dependent degradation in the cytoplasm. Thus, ubiquitination-mediated ERAD functions to rapidly clear misfolded proteins and alleviate ER stress.

At the same time, under ER stress, UPR increases protein-folding capacity of ER and decreases the generation of misfolded proteins. UPR is generally initiated by the activation of three ER transmembrane sensors: IRE1, PERK, and ATF6 (Oakes and Papa 2015). These sensors usually bind to an ER molecular chaperone BIP (GRP78 or HSPA5), and exist as inactive forms under the non-stressful conditions. When ER stress occurs, accumulated unfolded proteins recruit GRP78 and drive its dissociation from IRE1, PERK, and ATF6. Free IRE1, PERK, and ATF6 are activated, and trigger the activation of transcription factors XBP1s, ATF4, and ATF6(N) to selectively upregulate targets that can expand ER capacity to fold proteins, and simultaneously inhibits the activity of translation initiation factor eIF2α to slow down global protein synthesis and decrease the protein load on the ER (Clarke et al. 2014; Iurlaro and Muñoz-Pinedo 2016). Recently, increasing numbers of studies showed that ubiquitination regulates UPR by targeting ER sensors.

First, several E3 ligases regulate IRE1. An E3 ligase CHIP was found to catalyze K63-linked ubiquitination on K545 and K828 of IRE1, which promotes IRE1 phosphorylation and IRE1–TRAF2 interaction, and resists to ER-stressed-mediated senescence (Zhu et al. 2014). Another E3 ligase MITOL can also promote K63-linked IRE1 ubiquitination, albeit at a different lysine residue (K481), and protect cells from stress-induced apoptosis (Takeda et al. 2019). Notably, E3 HRD1-contained ERAD complex can target IRE1 for ubiquitination and degradation, which proposes a self-regulatory mechanism of the ER quality control system (Sun et al. 2015). Second, BRCA1 controls ER homeostasis by targeting PERK and IRE1 for ubiquitination and proteasomal degradation (Hromas et al. 2022). BRCA1 recruits both PERK and IRE1, which suggests that BRCA1, PERK, and IRE1 could form a complex under ER stress; BRCA1 deficiency leads to the accumulation of PERK and IRE1, which constitutively activates UPR and promotes survival of BRCA1-deficient cancer cells. Interestingly, under ER stress, PERK phosphorylates several E3 Ub ligases (MARCH5, MULAN, and Parkin), which increases the Ub ligases activities of these E3s (Toyofuku et al. 2020). LRRK2 can regulate PERK-mediated phosphorylation of these E3 ligases and determine ER-mitochondrial tethering. Third, ATF6 had been shown to be a direct target of the UPS (Hong et al. 2004). However, the E3 ligase that is responsible for ATF6 degradation remains unknown. A recent study demonstrated that an ER-localized E3 ligase RNF186 can catalyze non-proteolytic ubiquitination of K152 on ATF6 and improve UPR (Ranjan et al. 2021). Thus, three critical UPR sensors can be modified by ubiquitination under ER stress.

Ubiquitination also manipulates UPR by targeting transcription factors downstream of UPR pathways. SCF^βTrCP^ complex can bind to phosphorylated ATF4, which results in ATF4 ubiquitination and degradation (Lassot et al. 2001). Histone acetyltransferase p300 and casein kinase 1 delta (CK1δ) show roles on SCF^βTrCP^-mediated ATF4 ubiquitination (Lassot et al. 2005; Feng et al. 2021). Also, Ub conjugated on K60 and K77 of XBP1s had been reported to be required for its degradation, with unknown E3 ligases (Sun et al. 2020). Finally, translation initiation factor eIF2α is also modulated by some E3 ligases. HRD1 interacts with, ubiquitinates, and degrades eIF2α; however, another E3 RNF4 ubiquitinates and stabilizes phosphorylated eIF2α (Huang et al. 2017; Avitan-Hersh et al. 2020). Via targeting transcription factors of UPR, E3 ligases could directly guide UPR and control ER stress response. How these E3s cooperate with each other deserves further investigation.

In summary, to alleviate ER stress and maintain cell survival, ubiquitination-dependent ERAD and ubiquitin-regulated UPR are rapidly activated to degrade accumulated unfolded substrates or suppress the generation of unfold proteins. Moreover, a self-regulatory mechanism for ER recovery can be executed by ERAD-mediated ubiquitination of some components of UPR pathways.

### Oxidative stress

As one kind of extremely harmful products in cells, reactive oxidants can cause damages to various biological macromolecules, such as proteins, carbohydrates, nucleic acids, and lipids (Sies et al. 2017). A relative balance between oxidants and antioxidants is required for normal cellular functions. Reactive oxidants usually contain ROS, including superoxide anion radical (O_2_^•–^), hydrogen peroxide (H_2_O_2_), hydroxyl radical (HO^•^), and reactive nitrogen species (RNS), i.e., nitric oxide (NO^•^) and peroxynitrite anion (ONOO^–^) (Hayes et al. 2020). A relative excess of ROS or RNS when compared with antioxidants is defined as oxidative stress, which could be triggered by most environmental or intercellular stressors, including hypoxia, heat stress, DNA damage stress, ER stress, and other stresses that are not reviewed here. Cells possess multiple mechanisms to overcome oxidative stress and provide effective prevention of damages, which can enable cell survival. However, sustained oxidative stress may result in cell death (ferroptosis) and human diseases.

ROS can be produced in cytosol, but predominantly from the mitochondria, which requires multiple enzymes. NADPH oxidases (NOX enzymes), mitochondrial complexes I/II/III, superoxide dismutases (SOD1/2/3), cytochromes P-450, NADH oxidases, and many other oxidases are usually responsible for ROS generation; however, catalase, thioredoxin, glutathione peroxidase (GPX1-8), Peroxiredoxins (Prx1-6), glutathione (GSH), and other antioxidant factors can counteract ROS or ROS-induced damages (Sies et al. 2017). Ubiquitination or other PTMs of these enzymes that generate ROS or antioxidants definitely can modulate cellular responses to oxidative stress.

Many E3 Ub ligases have shown regulatory roles on ROS production by targeting these critical enzymes. First, several E3s can modulate the functions of NADPH oxidases. A HECT ligase HACE1 controls ROS generation by targeting Rac1-dependent NADPH oxidase complexes (Daugaard et al. 2013). Loss of HACE1 in human cell lines led to NADPH-oxidase-dependent ROS elevation, which further causes DNA damage. Notably, the study showed that Hace2 directly targeted Rac1, but not NADPH oxidase. Another E3 ligase Fbxw7 can directly bind to and degrade NADPH oxidase 1 (Nox1) (Wang et al. 2021). RNF34 ablation was also reported to enhance the generation of NADPH oxidase-dependent ROS (Fang et al. 2021). Although their target substrates are different, Hac1, Fbxw7, and RNF34 have similar inhibitory roles on NADPH oxidase-mediated ROS production. Besides NADPH oxidases, superoxide dismutase SOD1 can also be targeted by ubiquitination. Mitochondrial E3 ligase MITOL can ubiquitinate and degrade mutant SOD1, but not wild-type SOD1, which suppresses mutant SOD1-induced ROS generation (Yonashiro et al. 2009). Moreover, antioxidant proteins could be regulated by some E3 ligases. For example, antioxidant peroxidases 1 (Prx1) had been found to undergo E6AP-mediated ubiquitination and degradation. E6AP deficiency leads to the elevation of Prx1 and an enhanced cell capacity to tolerate an oxidative stress situation (Nasu et al. 2010; Wolyniec et al. 2013). In addition, other E3 ligases, including TRIM32 and TRAF6, have shown regulatory roles on mitochondrial ROS production (Prajapati et al. 2020; Shen et al. 2020; Wang et al. 2022b). There, targeting ROS-associated enzymes by ubiquitination could be a powerful strategy cells use to manipulate oxidative stress responses.

Furthermore, ROS sensor Keap1 is involved in controlling ubiquitination and stability of Nrf2, a transcription factor that plays a key role in oxidative stress resistance. Under oxidative stress, Nrf2 can trigger the expression of diverse cytoprotective proteins, including antioxidant, detoxification, and anti-inflammatory proteins (Kaspar et al. 2009). Keap1 possesses a BTB domain, a C-terminal Kelch domain, and an intervening region (IVR), and functions as the substrate recognition module of a Cullin3-based E3 ligase complex (Keap1/Cullin3/Rbx1) (Dinkova-Kostova et al. 2017). Under non-stressful conditions, dimeric Keap1 recruits an Nrf2 molecule, which facilitates Nrf2 polyubiquitination and subsequent proteasome-mediated degradation (Furukawa and Xiong 2005). Interestingly, human Keap1 is a cysteine-rich protein containing 27 highly conserved cysteines. These reactive cysteine residues can act as electrophilic sensors for cellular ROS. Once oxidative stress occurs, these cysteines of Keap1, especially Cys151 in the BTB domain and Cys273 and Cys288 in the IVR domain, can undergo oxidative modification, leading to Keap1 conformational change and Nrf2 dissociation from Keap1 CRL complex (Kobayashi et al. 2006; Rachakonda et al. 2008). The Nrf2 dissociation from Keap1 blocks the E3 activity of the Keap1 complex, stabilizes Nrf2, and promotes its nuclear accumulation, which initiates anti-ROS gene expression and contributes to cell survival in the stress. Collectively, ubiquitination is a critical executor in the Keap1-Nrf2 system to regulate Nrf2 stability and anti-ROS cellular responses.

Additionally, the N-end rule pathway acts as a nitric oxide sensor to control E3 ligases-mediated degradation of regulatory proteins bearing N-terminal cysteine (Hu et al. 2005, 2008). The N-end rule pathway is a proteolytic pathway that can destabilize N-terminal residues of target proteins (Varshavsky 2011). N-terminal cysteine of target substrates, such as RGS proteins, can be oxidated by nitric oxide, which is required for substrate arginylation by R-transferase ATE1. Arginylated cysteine of target proteins is further recognized, ubiquitinated, and degraded by E3 Ub ligases (including UBR1 and UBR2) of the N-end rule pathway. Therefore, by triggering ubiquitination-dependent substrate degradation, the N-end rule pathway regulates cellular response after sensing RNS/ROS levels.

Interestingly, ER stress response can also regulate oxidative stress response. On one hand, protein folding events on the ER produce a large volume of ROS as a byproduct, which directly has impacts on cellular oxidative stress. On the other hand, ROS accumulation within ER can cause excess oxidation of ER-located proteins or disrupt disulfide bond formation, which results in protein conformational changes or misfolded or unfold protein generation on the ER, thereby leading to ER stress and UPR (Cao and Kaufman 2014). UPR has shown critical roles in oxidative stress response via multiple molecular mechanisms, which has been summarized in a recent review (Ong and Logue 2023). Thus, it could be concluded that ubiquitination could regulate oxidative stress by controlling UPR.

#### Mitophagy

Mitophagy is an evolutionarily conserved mechanism that eliminates ROS-producing mitochondria via autophagy, which has a complex relationship with oxidative stress (Schofield and Schafer 2021). As the major source of intracellular ROS, mitochondria are constantly challenged and damaged by ROS accumulation. Mitophagy plays a fundamental role in the mitochondrial quality and quantity control via selective degradation of damaged mitochondria. There are two types of mitophagy in mammals: receptor-mediated mitophagy and Ub-mediated mitophagy (Onishi et al. 2021).

Ubiquitination can modulate receptor-mediated mitophagy. Mitophagy receptors on the OMM, such as BNIP3, BNIP3L (NIX), and FUNDC1, have conserved LC3-interacting regions (LIRs) and can recruit the autophagic machinery to degrade mitochondria (Youle and Narendra 2011). Interestingly, RBR E3 ligase parkin mediates ubiquitination of NIX, which in turn recruits autophagy adaptor NBR1 and promotes autophagosome formation surrounding mitochondria (Gao et al. 2015). Moreover, E3 ligase MARCH5 also regulates receptor-mediated mitophagy by controlling FUNDC1 protein levels (Chen et al. 2017). MARCH5 directly binds to FUNDC1 and catalyze proteolytic ubiquitination at Lys119 of FUNDC1, which impairs hypoxia-induced mitophagy. Thus, ubiquitination shows a regulatory role in receptor-mediated mitophagy.

On the other hand, the mitochondrially localized serine-threonine kinase PINK1 and the E3 ligase Parkin exhibit central roles in ubiquitin-mediated mitophagy. Once mitochondria are damaged and mitochondrial membrane potential is changed, PINK1 forms a dimer and its Ser228 is phosphorylated via autophosphorylation (Okatsu et al. 2012). This autophosphorylation recruits, phosphorylates, and activates Parkin on Ser65 in its Ubl domain. Moreover, PINK1 autophosphorylation can also stabilize its Insert 3 regions to an appropriate position, which recruits Ub and promotes Ser65 phosphorylation of Ub; Importantly, Ub phosphorylation by PINK1 accelerates Parkin E3 ligase activity (Kane et al. 2014; Kazlauskaite et al. 2014; Koyano et al. 2014). This PINK1-Parkin axis results in poly-p-Ub accumulation on damaged mitochondria, which recruits a series of autophagy adaptors (p62/SQSTM1, NDP52/CALCOCO2, NBR1, OPTN, TAX1BP1) and LC3, finally facilitating autophagosome closure and autophagosome-lysosome fusion (Ordureau et al. 2014; Onishi et al. 2021). Collectively, mitochondria-generated excessive ROS harms mitochondria, which triggers ubiquitin-regulated or mediated mitophagy to remove damaged mitochondria and alleviate oxidative stress to protect cells.

#### Ferroptosis

Ferroptosis, one type of cell death that can be triggered by excessive ROS stress, is modulated by the ubiquitination system. Ferroptosis is an iron-dependent and non-apoptotic form of regulated cell death, which is characterized by the accumulation of ROS and lipid peroxidation products (Li et al. 2020c). By regulating lipid peroxidation and iron metabolism, some proteins show very important effects on the ferroptosis process, including p53, GPX4, SLC7A11, FSP1, HSPB1, VDAC2/3, and NRF2 (Xie et al. 2016). Ub covalently conjugated to these molecules usually have important impacts on ferroptosis.

SLC7A11, a crucial subunit of the cystine/glutamate antiporter to block ferroptosis, had been found to be ubiquitinated by E3 ligase TRIM26 and deubiquitinated by OTUB1 (Liu et al. 2019b; Zhu et al. 2021b). While OTUB1 stabilizes SLC7A11 to prevent ferroptosis, TRIM26-mediated ubiquitination degrades SLC7A11 and enhances ferroptosis. Two recent studies had also reported that a SOCS2-containing E3 complex or HRD1 can interact with SLC7A11 and catalyze K48-linked proteolytic ubiquitination of SLC7A11, thus promoting ferroptosis (Chen et al. 2023; Wang et al. 2023). Moreover, another key ferroptosis regulator GPX4 is modulated by ubiquitination. A novel small molecule inhibitor (Bufotalin) of GPX4 was recently reported to induce GPX4 ubiquitination and degradation, lipid peroxidation, and ferroptosis, and thus could serve as potential anti-tumor agents (Zhang et al. 2022). Although the E3 ligase that mediates Bufotalin-induced GPX4 degradation remains unknown, several E3 ligases target GPX4. Recent studies have demonstrated that TRIM21, TRIM59, or NEDD4L promotes ferroptosis by inducing K48-linked ubiquitination and degradation of GPX4 (Sun et al. 2023; Tang et al. 2023; Zhang et al. 2023). However, LUBAC E3 complex recruits GPX4 and catalyzes M1-linked linear ubiquitination of GPX4, which can stabilize GPX4 and improve cellular defenses against ferroptosis (Dong et al. 2022). Interestingly, preexisting K48- or K63-linked Ub chains on GPX4 could enable LUBAC recruitment to GPX4. Based on these studies, a complicated Ub-associated mechanism for ferroptosis regulation could be proposed. K48-linked ubiquitination can lead to GPX4 degradation, which either promotes ferroptosis or recruits LUBAC to product linear Ub chains on GPX4 and thus block the ferroptosis process. Additionally, voltage-dependent anion channel proteins VDAC2/3 bind to the ferroptosis activator (erastin) and undergo ubiquitination and degradation, which is mediated by a special E3 ligase Nedd4 (Yang et al. 2020). Nedd4 expression is induced by erastin, which exacerbates VDAC2/3 degradation and thus enhances cell tolerance to erastin treatment. FBXW7 also targets VDAC3 for ubiquitination and degradation (Zhu et al. 2021a). Finally, some other E3 ligases can modulate other regulatory proteins of ferroptosis. For example, TRIM69 E3 ligase ubiquitinates and degrades FSP1 (ferroptosis suppressor protein 1) to promote ferroptosis (Yuan et al. 2022). However, two E3 ligases MARCHF6 and HUWE1 can target p53, ACSL4, and transferrin receptor for ubiquitination and proteosome-mediated degradation, which suppresses ferroptosis (Nguyen et al. 2022; Wu et al. 2022). Given that ferroptosis is currently a research focus that attracts much attention, roles of more E3 ligases in regulating ferroptosis will be explored in the recent futures.

Collectively, via targeting some crucial ROS enzymes, the Keap1-Nrf2 axis, the UPR process, mitophagy, and ferroptosis, the ubiquitination system exhibits critical functions to regulate cellular adaptive responses to oxidative stress.

## Conclusions and future perspectives

In summary, as one fundamental and omnipresent PTM in mammals, ubiquitination shows important regulatory functions in controlling distinct cellular responses to different stressors. Multiple elements of cellular response pathways, such as receptors, adaptors, signal transducers, or transcription factors, are modified and controlled by the ubiquitination system, which thus manages stresses, repairs damages, and restores homeostasis. Although ubiquitination targets different substrates in different cellular stress responses, it renders cells the capacity to resist stresses, showing housekeeping roles in sustaining cellular homeostasis.

As described above, under some stresses, roles of ubiquitination in cellular responses remain poorly investigated, such as extreme cold stress. Some mammals can tolerate extreme cold stress (close to 0°C) during hibernation (Mohr et al. 2020), however, whether ubiquitination or other PTMs, modulates the tolerance to extreme cold is unclear. Moreover, human skin (in frostbite) and tissues (in organ preservation) also undergo extreme cold stress (Petrone et al. 2014; Jing et al. 2018). Further investigations are still needed to explore whether ubiquitination is involved in cellular protection against cold-induced damage.

In some cellular stress response pathways, one critical component can be targeted by multiple E3s-mediated ubiquitination. Some E3s promote the activity of the substrate, while other E3s inactivate or destroy the substrate. How cell coordinates these E3 ligases to act, and how they regulate each other in stress responses deserve more integrated investigations.

Significant advances have been addressed in understanding classical ubiquitination system in the last decades, but emerging evidence highlights the importance of atypical ubiquitination. On one hand, more atypical E3 ligases may exist. The discovery of an E3 ligase MYCB1 having esterification activity and targeting threonine residues suggests that a group of human E3 ligases may be selectively responsible for non-lysine ubiquitination. Studies of bacterial E1/E2-independent E3 ligases also lead to a possibility that mammalian cells may express similar E3 Ub ligases. On the other hand, non-proteinaceous substrates are needed to be further explored. To date, only bacterial LPS and eukaryotic phospholipids PE were reported to be ubiquitinated by E3 ligases RNF213 and Tul1, respectively, proposing a novel class of ubiquitination substrates beyond protein (Otten et al. 2021; Sakamaki et al. 2022). However, it is unknown if other non-proteinaceous materials, carbohydrates, or nucleic acids, could undergo ubiquitination. It is expected that exploring atypical E3 ligases and identifying non-proteinaceous targets that regulate cellular stress responses will greatly extend our knowledge of the ubiquitination system.

Currently, there is high interest in academia and industry in exploring tissue- and cell-type-specific expression profiles of human E3 ligases under normal or diseased/stressed conditions, especially for the development of targeted protein degradation (TPD) (Békés et al. 2022). TPD is an attractive therapeutic modality to degrade disease-causing proteins via designed strategies, of which proteolysis-targeting chimeras (PROTACs) is the best-known technology showing great therapeutic potentials in oncology, inflammation, autoimmune diseases, virus infection, and neurodegeneration. A PROTAC molecule is a small heterobifunctional compound that contains two linked ligands, which can bridge a choice E3 ligase and a protein of interest (POI), leading to POI ubiquitination and proteasomal degradation (Sakamoto et al. 2001; Toure and Crews 2016). In the last two decades, the PROTAC field grows very exponentially and several PROTAC degraders have been entered in preclinical and early clinical development. Although a systematic assessment of protein targets that are suitable to be degraded by PROTAC molecules had been performed (Schneider et al. 2021), only a few validated E3 ligases are utilized in PROTAC, such as CRBN, MDM2, VHL, DCAFs, IAPs, KLHLs, RNF4, RNF114, KEAP1, TRIM9, TRIM71, and ZNRF3 (Békés et al. 2022; Marei et al. 2022). Although PROTAC degraders that use these validated E3 ligases work very well, there is still a need to explore now E3 ligases for PROTAC. Based on some recent studies, it could be proposed that, for a specified target, one E3 ligase in particular may work better than others (Bondeson et al. 2018; Bond et al. 2020; Zeng et al. 2020). Moreover, in oncology, resistance mechanisms to PROTAC agents could be developed rapidly by cancer cells to inhibit the abilities of PROTAC E3 ligases and therefore evade degraders, which may require alternative ligases for developing next-generation degraders. Therefore, for development of PROTAC, it is very important to identify new E3 ligases to pair with any target of choice. Several factors can determine the degradation profiles of E3 ligase: shape complementarity of the ligase and the target, degradation-competent ternary complex formation, subcellular localization of ligase and target, and cell-type- or tissue-specific expression profiles of E3 ligases (Békés et al. 2022). Using PROTAC molecules based on cell-type- or tissue-specific E3 ligases can degrade target proteins in the special cancer cells or tissues/organs, which could avoid systemic off-targets and side effects. Several studies had analyzed expression profile of E3 ligases in healthy and diseased states according to the publicly available datasets (He et al. 2020; Khan et al. 2020; Shirasaki et al. 2021). However, analysis of expression levels of a single subunit of multi-subunit E3 complexes in these studies cannot represent the accurate expression profiles of the E3 complexes. Collectively, to systematically investigate tissue- and cell-type-specific expression profiles of E3 Ub ligases, under normal or diseased/stressed states, will be an attracting topic in the next decade either to comprehensively understand the biology of E3 ligases in different diseases/stresses or to extend PROTAC applications.

## References

[CIT0001] Abdelmohsen K , Srikantan S, Yang X et al. Ubiquitin-mediated proteolysis of HuR by heat shock. EMBO J 2009;28:1271–82. 19322201 10.1038/emboj.2009.67 PMC2683047

[CIT0002] Ai H , Sun M, Liu A et al. H2B Lys34 Ubiquitination induces nucleosome distortion to stimulate Dot1L activity. Nat Chem Biol 2022;18:972–80. 35739357 10.1038/s41589-022-01067-7

[CIT0003] Akimov V , Barrio-Hernandez I, Hansen SV et al. UbiSite approach for comprehensive mapping of lysine and N-terminal ubiquitination sites. Nat Struct Mol Biol 2018;25:631–40. 29967540 10.1038/s41594-018-0084-y

[CIT0004] Akman M , Belisario DC, Salaroglio IC et al. Hypoxia, endoplasmic reticulum stress and chemoresistance: dangerous liaisons. J Exp Clin Cancer Res 2021;40:1–17. 33423689 10.1186/s13046-020-01824-3 PMC7798239

[CIT0005] Akturk A , Wasilko DJ, Wu X et al. Mechanism of phosphoribosyl-ubiquitination mediated by a single Legionella effector. Nature 2018;557:729–33. 29795346 10.1038/s41586-018-0147-6 PMC5980775

[CIT0006] Albanese A , Daly LA, Mennerich D et al. The role of hypoxia-inducible factor post-translational modifications in regulating its localisation, stability, and activity. Int J Mol Sci 2020;22:268. 33383924 10.3390/ijms22010268 PMC7796330

[CIT0007] Aprile-Garcia F , Tomar P, Hummel B et al. Nascent-protein ubiquitination is required for heat shock–induced gene downregulation in human cells. Nat Struct Mol Biol 2019;26:137–46. 30723328 10.1038/s41594-018-0182-x

[CIT0008] Aravind L , Koonin EV. The U box is a modified RING finger—a common domain in ubiquitination. Curr Biol 2000;10:R132–4. 10704423 10.1016/s0960-9822(00)00398-5

[CIT0009] Asaoka T , Almagro J, Ehrhardt C et al. Linear ubiquitination by LUBEL has a role in Drosophila heat stress response. EMBO Rep 2016;17:1624–40. 27702987 10.15252/embr.201642378 PMC5090701

[CIT0010] Avitan-Hersh E , Feng Y, Vaisman AO et al. Regulation of eIF2α by RNF4 promotes melanoma tumorigenesis and therapy resistance. J Investig Dermatol 2020;140:2466–77. 32360601 10.1016/j.jid.2020.04.008 PMC8081033

[CIT0011] Balaji V , Hoppe T. Regulation of E3 ubiquitin ligases by homotypic and heterotypic assembly. F1000Res 2020;9:1–8. 10.12688/f1000research.21253.1 PMC7005916 32076548

[CIT0012] Ballinger CA , Connell P, Wu Y et al. Identification of CHIP, a novel tetratricopeptide repeat-containing protein that interacts with heat shock proteins and negatively regulates chaperone functions. Mol Cell Biol 1999;19:4535–45. 10330192 10.1128/mcb.19.6.4535 PMC104411

[CIT0013] Bankir L , Bouby N, Trinh-Trang-Tan M-M. The role of the kidney in the maintenance of water balance. Baillieres Clin Endocrinol Metab 1989;3:249–311. 2698139 10.1016/s0950-351x(89)80005-9

[CIT0014] Barford D. Structural interconversions of the anaphase-promoting complex/cyclosome (APC/C) regulate cell cycle transitions. Curr Opin Struct Biol 2020;61:86–97. 31864160 10.1016/j.sbi.2019.11.010

[CIT0015] Barghouth PG , Thiruvalluvan M, LeGro M et al. DNA damage and tissue repair: what we can learn from planaria. Semin Cell Dev Biol 2019;87:145–59. 29727725 10.1016/j.semcdb.2018.04.013 PMC7039696

[CIT0016] Barnes BM. Freeze avoidance in a mammal: body temperatures below 0°C in an arctic hibernator. Science 1989;244:1593–5. 2740905 10.1126/science.2740905

[CIT0017] Bartke T , Pohl C, Pyrowolakis G et al. Dual role of BRUCE as an antiapoptotic IAP and a chimeric E2/E3 ubiquitin ligase. Mol Cell 2004;14:801–11. 15200957 10.1016/j.molcel.2004.05.018

[CIT0018] Bassermann F , Frescas D, Guardavaccaro D et al. The Cdc14B-Cdh1-Plk1 axis controls the G2 DNA-damage-response checkpoint. Cell 2008;134:256–67. 18662541 10.1016/j.cell.2008.05.043 PMC2591934

[CIT0019] Becker JR , Clifford G, Bonnet C et al. BARD1 reads H2A lysine 15 ubiquitination to direct homologous recombination. Nature 2021;596:433–7. 34321663 10.1038/s41586-021-03776-w PMC7619424

[CIT0020] Békés M , Langley DR, Crews CM. PROTAC targeted protein degraders: the past is prologue. Nat Rev Drug Discovery 2022;21:181–200. 35042991 10.1038/s41573-021-00371-6 PMC8765495

[CIT0021] Bento CF , Fernandes R, Ramalho J et al. The chaperone-dependent ubiquitin ligase CHIP targets HIF-1α for degradation in the presence of methylglyoxal. PLoS One 2010;5:e15062. 21124777 10.1371/journal.pone.0015062 PMC2993942

[CIT0022] Bettaieb A , Averill-Bates DA. Thermotolerance induced at a mild temperature of 40 C alleviates heat shock-induced ER stress and apoptosis in HeLa cells. Biochim Biophys Acta, Mol Cell Res 2015;1853:52–62. 10.1016/j.bbamcr.2014.09.016 25260982

[CIT0023] Bhogaraju S , Kalayil S, Liu Y et al. Phosphoribosylation of ubiquitin promotes serine ubiquitination and impairs conventional ubiquitination. Cell 2016;167:1636–1649.e13. 27912065 10.1016/j.cell.2016.11.019

[CIT0024] Bhogaraju S , Bonn F, Mukherjee R et al. Inhibition of bacterial ubiquitin ligases by SidJ–calmodulin catalysed glutamylation. Nature 2019;572:382–6. 31330532 10.1038/s41586-019-1440-8 PMC6715450

[CIT0025] Black MH , Osinski A, Gradowski M et al. Bacterial pseudokinase catalyzes protein polyglutamylation to inhibit the SidE-family ubiquitin ligases. Science 2019;364:787–92. 31123136 10.1126/science.aaw7446 PMC6767918

[CIT0026] Bohgaki T , Bohgaki M, Cardoso R et al. Genomic instability, defective spermatogenesis, immunodeficiency, and cancer in a mouse model of the RIDDLE syndrome. PLoS Genet 2011;7:e1001381. 21552324 10.1371/journal.pgen.1001381 PMC3084200

[CIT0027] Bohgaki M , Bohgaki T, El Ghamrasni S et al. RNF168 ubiquitylates 53BP1 and controls its response to DNA double-strand breaks. Proc Natl Acad Sci USA 2013;110:20982–7. 24324146 10.1073/pnas.1320302111 PMC3876264

[CIT0028] Bond U , Schlesinger MJ. Ubiquitin is a heat shock protein in chicken embryo fibroblasts. Mol Cell Biol 1985;5:949–56. 2987683 10.1128/mcb.5.5.949-956.1985 PMC366809

[CIT0029] Bond U , Schlesinger MJ. The chicken ubiquitin gene contains a heat shock promoter and expresses an unstable mRNA in heat-shocked cells. Mol Cell Biol 1986;6:4602–10. 3025663 10.1128/mcb.6.12.4602-4610.1986 PMC367245

[CIT0030] Bond MJ , Chu L, Nalawansha DA et al. Targeted degradation of oncogenic KRASG12C by VHL-recruiting PROTACs. ACS Cent Sci 2020;6:1367–75. 32875077 10.1021/acscentsci.0c00411 PMC7453568

[CIT0031] Bondeson DP , Smith BE, Burslem GM et al. Lessons in PROTAC design from selective degradation with a promiscuous warhead. Cell Chem Biol 2018;25:78–87. 29129718 10.1016/j.chembiol.2017.09.010 PMC5777153

[CIT0032] Brocker C , Thompson DC, Vasiliou V. The role of hyperosmotic stress in inflammation and disease. Biomol Concepts 2012;3:345–64. 22977648 10.1515/bmc-2012-0001 PMC3438915

[CIT0033] Bruick RK , McKnight SL. A conserved family of prolyl-4-hydroxylases that modify HIF. Science 2001;294:1337–40. 11598268 10.1126/science.1066373

[CIT0034] Burgos JI , Morell M, Mariángelo JIE et al. Hyperosmotic stress promotes endoplasmic reticulum stress-dependent apoptosis in adult rat cardiac myocytes. Apoptosis 2019;24:785–97. 31309362 10.1007/s10495-019-01558-4

[CIT0035] Cairó M , Campderrós L, Gavaldà-Navarro A et al. Parkin controls brown adipose tissue plasticity in response to adaptive thermogenesis. EMBO Rep 2019;20:e46832. 30867164 10.15252/embr.201846832 PMC6501052

[CIT0036] Calzado MA , De La Vega L, Möller A et al. An inducible autoregulatory loop between HIPK2 and Siah2 at the apex of the hypoxic response. Nat Cell Biol 2009;11:85–91. 19043406 10.1038/ncb1816

[CIT0037] Cao SS , Kaufman RJ. Endoplasmic reticulum stress and oxidative stress in cell fate decision and human disease. Antioxid Redox Signal 2014;21:396–413. 24702237 10.1089/ars.2014.5851 PMC4076992

[CIT0038] Cappadocia L , Lima CD. Ubiquitin-like protein conjugation: structures, chemistry, and mechanism. Chem Rev 2018;118:889–918. 28234446 10.1021/acs.chemrev.6b00737 PMC5815371

[CIT0039] Carlson N , Rogers S, Rechsteiner M. Microinjection of ubiquitin: changes in protein degradation in HeLa cells subjected to heat-shock. J Cell Biol 1987;104:547–55. 3029142 10.1083/jcb.104.3.547 PMC2114564

[CIT0040] Cassavaugh JM , Hale SA, Wellman TL et al. Negative regulation of HIF-1α by an FBW7-mediated degradation pathway during hypoxia. J Cell Biochem 2011;112:3882–90. 21964756 10.1002/jcb.23321 PMC3202039

[CIT0041] Catrina S-B , Okamoto K, Pereira T et al. Hyperglycemia regulates hypoxia-inducible factor-1α protein stability and function. Diabetes 2004;53:3226–32. 15561954 10.2337/diabetes.53.12.3226

[CIT0042] Chen D , Li M, Luo J et al. Direct interactions between HIF-1α and Mdm2 modulate p53 function. J Biol Chem 2003;278:13595–8. 12606552 10.1074/jbc.C200694200

[CIT0043] Chen P-C , Bhattacharyya BJ, Hanna J et al. Ubiquitin homeostasis is critical for synaptic development and function. J Neurosci 2011;31:17505–13. 22131412 10.1523/JNEUROSCI.2922-11.2011 PMC3253363

[CIT0044] Chen Z , Liu L, Cheng Q et al. Mitochondrial E3 ligase MARCH 5 regulates FUNDC 1 to fine-tune hypoxic mitophagy. EMBO Rep 2017;18:495–509. 28104734 10.15252/embr.201643309 PMC5331199

[CIT0045] Chen X , Li Z, Yong H et al. Trim21-mediated HIF-1α degradation attenuates aerobic glycolysis to inhibit renal cancer tumorigenesis and metastasis. Cancer Lett 2021;508:115–26. 33794309 10.1016/j.canlet.2021.03.023

[CIT0046] Chen Q , Zheng W, Guan J et al. SOCS2-enhanced ubiquitination of SLC7A11 promotes ferroptosis and radiosensitization in hepatocellular carcinoma. Cell Death Differ 2023;30:137–51. 35995846 10.1038/s41418-022-01051-7 PMC9883449

[CIT0047] Cheng L , Zhang W, Hu J et al. Characterization of the key region and key phosphorylation sites of EcaICE1 for its molecular interaction with EcaHOS1 protein in *Eucalyptus camaldulensis*. Res Square 2020. doi:10.21203/rs.2.21485/v1 33107181

[CIT0048] Clague MJ , Urbé S, Komander D. Breaking the chains: deubiquitylating enzyme specificity begets function. Nat Rev Mol Cell Biol 2019;20:338–52. 30733604 10.1038/s41580-019-0099-1

[CIT0049] Clarke HJ , Chambers JE, Liniker E et al. Endoplasmic reticulum stress in malignancy. Cancer Cell 2014;25:563–73. 24823636 10.1016/j.ccr.2014.03.015

[CIT0050] Clouaire T , Rocher V, Lashgari A et al. Comprehensive mapping of histone modifications at DNA double-strand breaks deciphers repair pathway chromatin signatures. Mol Cell 2018;72:250–262.e6. 30270107 10.1016/j.molcel.2018.08.020 PMC6202423

[CIT0051] Connell P , Ballinger CA, Jiang J et al. The co-chaperone CHIP regulates protein triage decisions mediated by heat-shock proteins. Nat Cell Biol 2001;3:93–6. 11146632 10.1038/35050618

[CIT0052] Crinelli R , Bianchi M, Radici L et al. Molecular dissection of the human ubiquitin C promoter reveals heat shock element architectures with activating and repressive functions. PLoS One 2015;10:e0136882. 26317694 10.1371/journal.pone.0136882 PMC4552642

[CIT0053] Cullen KE , Sarge KD. Characterization of hypothermia-induced cellular stress response in mouse tissues. J Biol Chem 1997;272:1742–6. 8999855 10.1074/jbc.272.3.1742

[CIT0054] Dai Q , Zhang C, Wu Y et al. CHIP activates HSF1 and confers protection against apoptosis and cellular stress. EMBO J 2003;22:5446–58. 14532117 10.1093/emboj/cdg529 PMC213783

[CIT0055] Dai Y , Zhang J, Xiang J et al. Calcitriol inhibits ROS-NLRP3-IL-1β signaling axis via activation of Nrf2-antioxidant signaling in hyperosmotic stress stimulated human corneal epithelial cells. Redox Biol 2019;21:101093. 30611121 10.1016/j.redox.2018.101093 PMC6313824

[CIT0056] Daks A , Petukhov A, Fedorova O et al. The RNA-binding protein HuR is a novel target of Pirh2 E3 ubiquitin ligase. Cell Death Dis 2021;12:1–14. 34091597 10.1038/s41419-021-03871-w PMC8179929

[CIT0057] Danno S , Nishiyama H, Higashitsuji H et al. Increased transcript level of RBM3, a member of the glycine-rich RNA-binding protein family, in human cells in response to cold stress. Biochem Biophys Res Commun 1997;236:804–7. 9245737 10.1006/bbrc.1997.7059

[CIT0058] Daugaard M , Nitsch R, Razaghi B et al. Hace1 controls ROS generation of vertebrate Rac1-dependent NADPH oxidase complexes. Nat Commun 2013;4:2180. 23864022 10.1038/ncomms3180 PMC3759041

[CIT0059] de Jager M , Dronkert ML, Modesti M et al. DNA-binding and strand-annealing activities of human Mre11: implications for its roles in DNA double-strand break repair pathways. Nucleic Acids Res 2001;29:1317–25. 11238998 10.1093/nar/29.6.1317 PMC29748

[CIT0060] den Engelsman J , Keijsers V, de Jong WW et al. The small heat-shock protein αB-crystallin promotes FBX4-dependent ubiquitination. J Biol Chem 2003;278:4699–704. 12468532 10.1074/jbc.M211403200

[CIT0061] Deng L , Meng T, Chen L et al. The role of ubiquitination in tumorigenesis and targeted drug discovery. Signal Transduct Target Ther 2020;5:1–28. 32296023 10.1038/s41392-020-0107-0 PMC7048745

[CIT0062] Densham RM , Garvin AJ, Stone HR et al. Human BRCA1–BARD1 ubiquitin ligase activity counteracts chromatin barriers to DNA resection. Nat Struct Mol Biol 2016;23:647–55. 27239795 10.1038/nsmb.3236 PMC6522385

[CIT0063] Deshaies RJ , Joazeiro CA. RING domain E3 ubiquitin ligases. Annu Rev Biochem 2009;78:399–434. 19489725 10.1146/annurev.biochem.78.101807.093809

[CIT0064] Dietz L , Ellison CJ, Riechmann C et al. Structural basis for SMAC-mediated antagonism of caspase inhibition by the giant ubiquitin ligase BIRC6. Science 2023;379:1112–7. 36758106 10.1126/science.ade8840

[CIT0065] Dikic I , Wakatsuki S, Walters KJ. Ubiquitin-binding domains—from structures to functions. Nat Rev Mol Cell Biol 2009;10:659–71. 19773779 10.1038/nrm2767 PMC7359374

[CIT0066] Dinkova-Kostova AT , Kostov RV, Canning P. Keap1, the cysteine-based mammalian intracellular sensor for electrophiles and oxidants. Arch Biochem Biophys 2017;617:84–93. 27497696 10.1016/j.abb.2016.08.005 PMC5339396

[CIT0067] Doil C , Mailand N, Bekker-Jensen S et al. RNF168 binds and amplifies ubiquitin conjugates on damaged chromosomes to allow accumulation of repair proteins. Cell 2009;136:435–46. 19203579 10.1016/j.cell.2008.12.041

[CIT0068] Dong C-H , Agarwal M, Zhang Y et al. The negative regulator of plant cold responses, HOS1, is a RING E3 ligase that mediates the ubiquitination and degradation of ICE1. Proc Natl Acad Sci USA 2006;103:8281–6. 16702557 10.1073/pnas.0602874103 PMC1472463

[CIT0069] Dong K , Wei R, Jin T et al. HOIP modulates the stability of GPx4 by linear ubiquitination. Proc Natl Acad Sci USA 2022;119:e2214227119. 36279464 10.1073/pnas.2214227119 PMC9636971

[CIT0070] Dou H , Buetow L, Sibbet GJ et al. Essentiality of a non-RING element in priming donor ubiquitin for catalysis by a monomeric E3. Nat Struct Mol Biol 2013;20:982–6. 23851457 10.1038/nsmb.2621 PMC4471106

[CIT0071] Dove KK , Klevit RE. RING-between-RING E3 ligases: emerging themes amid the variations. J Mol Biol 2017;429:3363–75. 28827147 10.1016/j.jmb.2017.08.008 PMC5675740

[CIT0072] Drané P , Brault M-E, Cui G et al. TIRR regulates 53BP1 by masking its histone methyl-lysine binding function. Nature 2017;543:211–6. 28241136 10.1038/nature21358 PMC5441565

[CIT0073] Duda DM , Olszewski JL, Schuermann JP et al. Structure of HHARI, a RING-IBR-RING ubiquitin ligase: autoinhibition of an Ariadne-family E3 and insights into ligation mechanism. Structure 2013;21:1030–41. 23707686 10.1016/j.str.2013.04.019 PMC3747818

[CIT0074] Eddins MJ , Varadan R, Fushman D et al. Crystal structure and solution NMR studies of Lys48-linked tetraubiquitin at neutral pH. J Mol Biol 2007;367:204–11. 17240395 10.1016/j.jmb.2006.12.065

[CIT0075] Ehrmann JF , Grabarczyk DB, Heinke M et al. Structural basis of how the BIRC6/SMAC complex regulates apoptosis and autophagy. bioRxiv 2022. doi:10.1101/2022.08.30.505823 36758105

[CIT0076] Elia AE , Wang DC, Willis NA et al. RFWD3-dependent ubiquitination of RPA regulates repair at stalled replication forks. Mol Cell 2015;60:280–93. 26474068 10.1016/j.molcel.2015.09.011 PMC4609029

[CIT0077] Epstein AC , Gleadle JM, McNeill LA et al. *C. elegans* EGL-9 and mammalian homologs define a family of dioxygenases that regulate HIF by prolyl hydroxylation. Cell 2001;107:43–54. 11595184 10.1016/s0092-8674(01)00507-4

[CIT0078] Escribano-Díaz C , Orthwein A, Fradet-Turcotte A et al. A cell cycle-dependent regulatory circuit composed of 53BP1-RIF1 and BRCA1-CtIP controls DNA repair pathway choice. Mol Cell 2013;49:872–83. 23333306 10.1016/j.molcel.2013.01.001

[CIT0079] Falck J , Coates J, Jackson SP. Conserved modes of recruitment of ATM, ATR and DNA-PKcs to sites of DNA damage. Nature 2005;434:605–11. 15758953 10.1038/nature03442

[CIT0080] Fang NN , Chan GT, Zhu M et al. Rsp5/Nedd4 is the main ubiquitin ligase that targets cytosolic misfolded proteins following heat stress. Nat Cell Biol 2014;16:1227–37. 25344756 10.1038/ncb3054 PMC5224936

[CIT0081] Fang S , Cheng Y, Deng F et al. RNF34 ablation promotes cerebrovascular remodeling and hypertension by increasing NADPH-derived ROS generation. Neurobiol Dis 2021;156:105396. 34015492 10.1016/j.nbd.2021.105396

[CIT0082] Fell VL , Schild-Poulter C. The Ku heterodimer: function in DNA repair and beyond. Mutat Res/Rev Mutat Res 2015;763:15–29. 25795113 10.1016/j.mrrev.2014.06.002

[CIT0083] Feng L , Chen J. The E3 ligase RNF8 regulates KU80 removal and NHEJ repair. Nat Struct Mol Biol 2012;19:201–6. 22266820 10.1038/nsmb.2211 PMC3888515

[CIT0084] Feng X , Jia Y, Zhang Y et al. Ubiquitination of UVRAG by SMURF1 promotes autophagosome maturation and inhibits hepatocellular carcinoma growth. Autophagy 2019;15:1130–49. 30686098 10.1080/15548627.2019.1570063 PMC6613838

[CIT0085] Feng L , Li M, Hu X et al. CK1δ stimulates ubiquitination-dependent proteasomal degradation of ATF4 to promote chemoresistance in gastric Cancer. Clin Trans Med 2021;11:e587. 10.1002/ctm2.587 PMC8516343 34709767

[CIT0086] Ferretti LP , Himmels S-F, Trenner A et al. Cullin3-KLHL15 ubiquitin ligase mediates CtIP protein turnover to fine-tune DNA-end resection. Nat Commun 2016;7:1–16. 10.1038/ncomms12628 PMC5007465 27561354

[CIT0087] Fierz B , Chatterjee C, McGinty RK et al. Histone H2B ubiquitylation disrupts local and higher-order chromatin compaction. Nat Chem Biol 2011;7:113–9. 21196936 10.1038/nchembio.501 PMC3078768

[CIT0088] Finan JD , Leddy HA, Guilak F. Osmotic stress alters chromatin condensation and nucleocytoplasmic transport. Biochem Biophys Res Commun 2011;408:230–5. 21463604 10.1016/j.bbrc.2011.03.131 PMC3104296

[CIT0089] Finley D , Özkaynak E, Varshavsky A. The yeast polyubiquitin gene is essential for resistance to high temperatures, starvation, and other stresses. Cell 1987;48:1035–46. 3030556 10.1016/0092-8674(87)90711-2

[CIT0090] Fischer DF , van Dijk R, van Tijn P et al. Long-term proteasome dysfunction in the mouse brain by expression of aberrant ubiquitin. Neurobiol Aging 2009;30:847–63. 18760506 10.1016/j.neurobiolaging.2008.06.009

[CIT0091] Foresti O , Rodriguez-Vaello V, Funaya C et al. Quality control of inner nuclear membrane proteins by the Asi complex. Science 2014;346:751–5. 25236469 10.1126/science.1255638

[CIT0092] Fornace AJ , Alamo I, Hollander MC et al. Ubiquftin mRNA is a major stress-induced transcript in mammalian cells. Nucleic Acids Res 1989;17:1215–30. 2537950 10.1093/nar/17.3.1215 PMC331738

[CIT0093] Foster RE , Nnakwe C, Woo L et al. Monoubiquitination of the nonhomologous end joining protein XRCC4. Biochem Biophys Res Commun 2006;341:175–83. 16412978 10.1016/j.bbrc.2005.12.166

[CIT0094] Fradet-Turcotte A , Canny MD, Escribano-Díaz C et al. 53BP1 is a reader of the DNA-damage-induced H2A Lys 15 ubiquitin mark. Nature 2013;499:50–4. 23760478 10.1038/nature12318 PMC3955401

[CIT0095] Freemont PS , Hanson IM, Trowsdale J. A novel cysteine-rich sequence motif. Cell 1991;64:483–4. 1991318 10.1016/0092-8674(91)90229-r

[CIT0096] French ME , Koehler CF, Hunter T. Emerging functions of branched ubiquitin chains. Cell Discov 2021;7:1–10. 33495455 10.1038/s41421-020-00237-y PMC7835216

[CIT0097] Fu Y , Wang H, Dai H et al. OTULIN allies with LUBAC to govern angiogenesis by editing ALK1 linear polyubiquitin. Mol Cell 2021;81:3187–3204.e7. 34157307 10.1016/j.molcel.2021.05.031

[CIT0098] Fujita J. Cold shock response in mammalian cells. J Mol Microbiol Biotechnol 1999;1:243–55. 10943555

[CIT0099] Fukuba H , Takahashi T, Jin HG et al. Abundance of aspargynyl-hydroxylase FIH is regulated by Siah-1 under normoxic conditions. Neurosci Lett 2008;433:209–14. 18280659 10.1016/j.neulet.2007.12.069

[CIT0100] Furukawa M , Xiong Y. BTB protein Keap1 targets antioxidant transcription factor Nrf2 for ubiquitination by the Cullin 3-Roc1 ligase. Mol Cell Biol 2005;25:162–71. 15601839 10.1128/MCB.25.1.162-171.2005 PMC538799

[CIT0101] Galanty Y , Belotserkovskaya R, Coates J et al. RNF4, a SUMO-targeted ubiquitin E3 ligase, promotes DNA double-strand break repair. Genes Dev 2012;26:1179–95. 22661229 10.1101/gad.188284.112 PMC3371407

[CIT0102] Galluzzi L , Yamazaki T, Kroemer G. Linking cellular stress responses to systemic homeostasis. Nat Rev Mol Cell Biol 2018;19:731–45. 30305710 10.1038/s41580-018-0068-0

[CIT0103] Gan N , Zhen X, Liu Y et al. Regulation of phosphoribosyl ubiquitination by a calmodulin-dependent glutamylase. Nature 2019;572:387–91. 31330531 10.1038/s41586-019-1439-1 PMC6855250

[CIT0104] Gankam-Kengne F , Couturier BS, Soupart A et al. Osmotic stress–induced defective glial proteostasis contributes to brain demyelination after hyponatremia treatment. J Am Soc Nephrol 2017;28:1802–13. 28122966 10.1681/ASN.2016050509 PMC5461785

[CIT0105] Gao F , Chen D, Si J et al. The mitochondrial protein BNIP3L is the substrate of PARK2 and mediates mitophagy in PINK1/PARK2 pathway. Hum Mol Genet 2015;24:2528–38. 25612572 10.1093/hmg/ddv017

[CIT0106] Gatti M , Pinato S, Maiolica A et al. RNF168 promotes noncanonical K27 ubiquitination to signal DNA damage. Cell Rep 2015;10:226–38. 25578731 10.1016/j.celrep.2014.12.021

[CIT0107] Goldknopf IL , Busch H. Isopeptide linkage between nonhistone and histone 2A polypeptides of chromosomal conjugate-protein A24. Proc Natl Acad Sci USA 1977;74:864–8. 265581 10.1073/pnas.74.3.864 PMC430507

[CIT0108] Graf C , Stankiewicz M, Nikolay R et al. Insights into the conformational dynamics of the E3 ubiquitin ligase CHIP in complex with chaperones and E2 enzymes. Biochemistry 2010;49:2121–9. 20146531 10.1021/bi901829f

[CIT0109] Grice GL , Lobb IT, Weekes MP et al. The proteasome distinguishes between heterotypic and homotypic lysine-11-linked polyubiquitin chains. Cell Rep 2015;12:545–53. 26190103 10.1016/j.celrep.2015.06.061 PMC4533228

[CIT0110] Gudjonsson T , Altmeyer M, Savic V et al. TRIP12 and UBR5 suppress spreading of chromatin ubiquitylation at damaged chromosomes. Cell 2012;150:697–709. 22884692 10.1016/j.cell.2012.06.039

[CIT0111] Guettouche T , Boellmann F, Lane WS et al. Analysis of phosphorylation of human heat shock factor 1 in cells experiencing a stress. BMC Biochem 2005;6:1–14. 15760475 10.1186/1471-2091-6-4 PMC1079796

[CIT0112] Gwon Y , Maxwell BA, Kolaitis R-M et al. Ubiquitination of G3BP1 mediates stress granule disassembly in a context-specific manner. Science 2021;372:eabf6548. 34739333 10.1126/science.abf6548 PMC8574224

[CIT0113] Ha G-H , Ji J-H, Chae S et al. Pellino1 regulates reversible ATM activation via NBS1 ubiquitination at DNA double-strand breaks. Nat Commun 2019;10:1–18. 30952868 10.1038/s41467-019-09641-9 PMC6450972

[CIT0114] Hammel M , Rey M, Yu Y et al. XRCC4 protein interactions with XRCC4-like factor (XLF) create an extended grooved scaffold for DNA ligation and double strand break repair. J Biol Chem 2011;286:32638–50. 21775435 10.1074/jbc.M111.272641 PMC3173232

[CIT0115] Hanna J , Meides A, Zhang DP et al. A ubiquitin stress response induces altered proteasome composition. Cell 2007;129:747–59. 17512408 10.1016/j.cell.2007.03.042

[CIT0116] Hayes JD , Dinkova-Kostova AT, Tew KD. Oxidative stress in cancer. Cancer Cell 2020;38:167–97. 32649885 10.1016/j.ccell.2020.06.001 PMC7439808

[CIT0117] He Y , Khan S, Huo Z et al. Proteolysis targeting chimeras (PROTACs) are emerging therapeutics for hematologic malignancies. J Hematol Oncol 2020;13:1–24. 32718354 10.1186/s13045-020-00924-z PMC7384229

[CIT0118] Heath RJ , Goel G, Baxt LA et al. RNF166 determines recruitment of adaptor proteins during antibacterial autophagy. Cell Rep 2016;17:2183–94. 27880896 10.1016/j.celrep.2016.11.005 PMC5192565

[CIT0119] Hershko A , Ciechanover A. The ubiquitin system. Annu Rev Biochem 1998;67:425–79. 9759494 10.1146/annurev.biochem.67.1.425

[CIT0120] Hindi NN , Elsakrmy N, Ramotar D. The base excision repair process: comparison between higher and lower eukaryotes. Cell Mol Life Sci 2021;78:7943–65. 34734296 10.1007/s00018-021-03990-9 PMC11071731

[CIT0121] Ho S-R , Mahanic CS, Lee Y-J et al. RNF144A, an E3 ubiquitin ligase for DNA-PKcs, promotes apoptosis during DNA damage. Proc Natl Acad Sci USA 2014;111:E2646–55. 24979766 10.1073/pnas.1323107111 PMC4084471

[CIT0122] Hon W-C , Wilson MI, Harlos K et al. Structural basis for the recognition of hydroxyproline in HIF-1α by pVHL. Nature 2002;417:975–8. 12050673 10.1038/nature00767

[CIT0123] Hong M , Li M, Mao C et al. Endoplasmic reticulum stress triggers an acute proteasome-dependent degradation of ATF6. J Cell Biochem 2004;92:723–32. 15211570 10.1002/jcb.20118

[CIT0124] Horn V , Uckelmann M, Zhang H et al. Structural basis of specific H2A K13/K15 ubiquitination by RNF168. Nat Commun 2019;10:1–12. 30988309 10.1038/s41467-019-09756-z PMC6465349

[CIT0125] Hromas R , Srinivasan G, Yang M et al. BRCA1 mediates protein homeostasis through the ubiquitination of PERK and IRE1. Iscience 2022;25:105626. 36471805 10.1016/j.isci.2022.105626 PMC9719099

[CIT0126] Hryciw DH , Ekberg J, Lee A et al. Nedd4-2 functionally interacts with ClC-5: involvement in constitutive albumin endocytosis in proximal tubule cells. J Biol Chem 2004;279:P54996–55007. 10.1074/jbc.M411491200 15489223

[CIT0127] Hu R-G , Sheng J, Qi X et al. The N-end rule pathway as a nitric oxide sensor controlling the levels of multiple regulators. Nature 2005;437:981–6. 16222293 10.1038/nature04027

[CIT0128] Hu R-G , Wang H, Xia Z et al. The N-end rule pathway is a sensor of heme. Proc Natl Acad Sci USA 2008;105:76–81. 18162538 10.1073/pnas.0710568105 PMC2224235

[CIT0129] Huang Y , Sun Y, Cao Y et al. HRD1 prevents apoptosis in renal tubular epithelial cells by mediating eIF2α ubiquitylation and degradation. Cell Death Dis 2017;8:3202. 29233968 10.1038/s41419-017-0002-y PMC5870601

[CIT0130] Huen MS , Grant R, Manke I et al. RNF8 transduces the DNA-damage signal via histone ubiquitylation and checkpoint protein assembly. Cell 2007;131:901–14. 18001825 10.1016/j.cell.2007.09.041 PMC2149842

[CIT0131] Huibregtse JM , Scheffner M, Beaudenon S et al. A family of proteins structurally and functionally related to the E6-AP ubiquitin-protein ligase. Proc Natl Acad Sci USA 1995;92:2563–7. 7708685 10.1073/pnas.92.7.2563 PMC42258

[CIT0132] Hunkeler M , Jin CY, Fischer ES. Structures of BIRC6-client complexes provide a mechanism of SMAC-mediated release of caspases. Science 2023;379:1105–11. 36758104 10.1126/science.ade5750

[CIT0133] Hwang J , Qi L. Quality control in the endoplasmic reticulum: crosstalk between ERAD and UPR pathways. Trends Biochem Sci 2018;43:593–605. 30056836 10.1016/j.tibs.2018.06.005 PMC6327314

[CIT0134] In S , Kim Y-I, Lee JE et al. RNF20/40-mediated eEF1BδL monoubiquitylation stimulates transcription of heat shock-responsive genes. Nucleic Acids Res 2019;47:2840–55. 30649429 10.1093/nar/gkz006 PMC6451099

[CIT0135] Ishida N , Nakagawa T, Iemura S-I et al. Ubiquitylation of Ku80 by RNF126 promotes completion of nonhomologous end joining-mediated DNA repair. Mol Cell Biol 2017;37:e00347–16. 27895153 10.1128/MCB.00347-16 PMC5288581

[CIT0136] Ismail IH , Gagné J-P, Genois M-M et al. The RNF138 E3 ligase displaces Ku to promote DNA end resection and regulate DNA repair pathway choice. Nat Cell Biol 2015;17:1446–57. 26502055 10.1038/ncb3259

[CIT0137] Iurlaro R , Muñoz-Pinedo C. Cell death induced by endoplasmic reticulum stress. FEBS J 2016;283:2640–52. 26587781 10.1111/febs.13598

[CIT0138] Jackson SP , Bartek J. The DNA-damage response in human biology and disease. Nature 2009;461:1071–8. 19847258 10.1038/nature08467 PMC2906700

[CIT0139] Jing L , Yao L, Zhao M et al. Organ preservation: from the past to the future. Acta Pharmacol Sin 2018;39:845–57. 29565040 10.1038/aps.2017.182 PMC5943901

[CIT0140] Joshi S , Singh AR, Durden DL. MDM2 regulates hypoxic hypoxia-inducible factor 1α stability in an E3 ligase, proteasome, and PTEN-phosphatidylinositol 3-kinase-AKT-dependent manner. J Biol Chem 2014;289:22785–97. 24982421 10.1074/jbc.M114.587493 PMC4132784

[CIT0141] Kaiser SE , Riley BE, Shaler TA et al. Protein standard absolute quantification (PSAQ) method for the measurement of cellular ubiquitin pools. Nat Methods 2011;8:691–6. 21743460 10.1038/nmeth.1649 PMC3196335

[CIT0142] Kalb R , Mallery DL, Larkin C et al. BRCA1 is a histone-H2A-specific ubiquitin ligase. Cell Rep 2014;8:999–1005. 25131202 10.1016/j.celrep.2014.07.025 PMC4382519

[CIT0143] Kamynina E , Debonneville C, Bens M et al. A novel mouse Nedd4 protein suppresses the activity of the epithelial Na+ channel. FASEB J 2001;15:204–14. 11149908 10.1096/fj.00-0191com

[CIT0144] Kane LA , Lazarou M, Fogel AI et al. PINK1 phosphorylates ubiquitin to activate Parkin E3 ubiquitin ligase activity. J Cell Biol 2014;205:143–53. 24751536 10.1083/jcb.201402104 PMC4003245

[CIT0145] Kaneko M , Iwase I, Yamasaki Y et al. Genome-wide identification and gene expression profiling of ubiquitin ligases for endoplasmic reticulum protein degradation. Sci Rep 2016;6:1–10. 27485036 10.1038/srep30955 PMC4971459

[CIT0146] Kang HJ , Kim HJ, Rih J-K et al. BRCA1 plays a role in the hypoxic response by regulating HIF-1α stability and by modulating vascular endothelial growth factor expression. J Biol Chem 2006;281:13047–56. 16543242 10.1074/jbc.M513033200

[CIT0147] Kaplan JH. Biochemistry of na, K-ATPase. Annu Rev Biochem 2002;71:511–35. 12045105 10.1146/annurev.biochem.71.102201.141218

[CIT0148] Karim M , Biquand E, Declercq M et al. Nonproteolytic K29-linked ubiquitination of the PB2 replication protein of influenza A viruses by proviral cullin 4-based E3 ligases. Mbio 2020;11:e00305–20. 32265326 10.1128/mBio.00305-20 PMC7157767

[CIT0149] Kaspar JW , Niture SK, Jaiswal AK. Nrf2: INrf2 (Keap1) signaling in oxidative stress. Free Radic Biol Med 2009;47:1304–9. 19666107 10.1016/j.freeradbiomed.2009.07.035 PMC2763938

[CIT0150] Kazlauskaite A , Kondapalli C, Gourlay R et al. Parkin is activated by PINK1-dependent phosphorylation of ubiquitin at Ser65. Biochem J 2014;460:127–39. 24660806 10.1042/BJ20140334 PMC4000136

[CIT0151] Ke Q , Costa M. Hypoxia-inducible factor-1 (HIF-1). Mol Pharmacol 2006;70:1469–80. 16887934 10.1124/mol.106.027029

[CIT0152] Kerscher O , Felberbaum R, Hochstrasser M. Modification of proteins by ubiquitin and ubiquitin-like proteins. Annu Rev Cell Dev Biol 2006;22:159–80. 16753028 10.1146/annurev.cellbio.22.010605.093503

[CIT0153] Khan S , He Y, Zhang X et al. PROteolysis TArgeting Chimeras (PROTACs) as emerging anticancer therapeutics. Oncogene 2020;39:4909–24. 32475992 10.1038/s41388-020-1336-y PMC7319888

[CIT0154] Khmelinskii A , Blaszczak E, Pantazopoulou M et al. Protein quality control at the inner nuclear membrane. Nature 2014;516:410–3. 25519137 10.1038/nature14096 PMC4493439

[CIT0155] Kim H , Chen J, Yu X. Ubiquitin-binding protein RAP80 mediates BRCA1-dependent DNA damage response. Science 2007;316:1202–5. 17525342 10.1126/science.1139621

[CIT0156] Kim E , Wang B, Sastry N et al. NEDD4-mediated HSF1 degradation underlies α-synucleinopathy. Hum Mol Genet 2016;25:211–22. 26503960 10.1093/hmg/ddv445 PMC4706110

[CIT0157] Kimura Y , Tanaka K. Regulatory mechanisms involved in the control of ubiquitin homeostasis. J Biochem 2010;147:793–8. 20418328 10.1093/jb/mvq044

[CIT0158] Kmiecik SW , Drzewicka K, Melchior F et al. Heat shock transcription factor 1 is SUMOylated in the activated trimeric state. J Biol Chem 2021;296:100324–14. 33493517 10.1016/j.jbc.2021.100324 PMC7949154

[CIT0159] Kobayashi A , Kang M-I, Watai Y et al. Oxidative and electrophilic stresses activate Nrf2 through inhibition of ubiquitination activity of Keap1. Mol Cell Biol 2006;26:221–9. 16354693 10.1128/MCB.26.1.221-229.2006 PMC1317630

[CIT0160] Koh MY , Darnay BG, Powis G. Hypoxia-associated factor, a novel E3-ubiquitin ligase, binds and ubiquitinates hypoxia-inducible factor 1α, leading to its oxygen-independent degradation. Mol Cell Biol 2008;28:7081–95. 18838541 10.1128/MCB.00773-08 PMC2593390

[CIT0161] Kolas NK , Chapman JR, Nakada S et al. Orchestration of the DNA-damage response by the RNF8 ubiquitin ligase. Science 2007;318:1637–40. 18006705 10.1126/science.1150034 PMC2430610

[CIT0162] Komander D. The emerging complexity of protein ubiquitination. Biochem Soc Trans 2009;37:937–53. 19754430 10.1042/BST0370937

[CIT0163] Koyano F , Okatsu K, Kosako H et al. Ubiquitin is phosphorylated by PINK1 to activate parkin. Nature 2014;510:162–6. 24784582 10.1038/nature13392

[CIT0164] Kwasna D , Rehman SAA, Natarajan J et al. Discovery and characterization of ZUFSP/ZUP1, a distinct deubiquitinase class important for genome stability. Mol Cell 2018;70:150–64. e156. 29576527 10.1016/j.molcel.2018.02.023 PMC5896202

[CIT0165] Lam YA , Pickart CM, Alban A et al. Inhibition of the ubiquitin-proteasome system in Alzheimer’s disease. Proc Natl Acad Sci USA 2000;97:9902–6. 10944193 10.1073/pnas.170173897 PMC27620

[CIT0166] Lando D , Peet DJ, Gorman JJ et al. FIH-1 is an asparaginyl hydroxylase enzyme that regulates the transcriptional activity of hypoxia-inducible factor. Genes Dev 2002;16:1466–71. 12080085 10.1101/gad.991402 PMC186346

[CIT0167] Laroia G , Cuesta R, Brewer G et al. Control of mRNA decay by heat shock-ubiquitin-proteasome pathway. Science 1999;284:499–502. 10205060 10.1126/science.284.5413.499

[CIT0168] Lassot I , Ségéral E, Berlioz-Torrent C et al. ATF4 degradation relies on a phosphorylation-dependent interaction with the SCFβTrCPUbiquitin ligase. Mol Cell Biol 2001;21:2192–202. 11238952 10.1128/MCB.21.6.2192-2202.2001 PMC86853

[CIT0169] Lassot I , Estrabaud E, Emiliani S et al. p300 modulates ATF4 stability and transcriptional activity independently of its acetyltransferase domain. J Biol Chem 2005;280:41537–45. 16219772 10.1074/jbc.M505294200

[CIT0170] Lee J-H , Paull TT. ATM activation by DNA double-strand breaks through the Mre11-Rad50-Nbs1 complex. Science 2005;308:551–4. 15790808 10.1126/science.1108297

[CIT0171] Lee J-W , Bae S-H, Jeong J-W et al. Hypoxia-inducible factor (HIF-1) α: its protein stability and biological functions. Exp Mol Med 2004;36:1–12. 15031665 10.1038/emm.2004.1

[CIT0172] Lee Y , Chou T-F, Pittman SK et al. Keap1/Cullin3 modulates p62/SQSTM1 activity via UBA domain ubiquitination. Cell Rep 2017;19:188–202. 28380357 10.1016/j.celrep.2017.03.030 PMC5395095

[CIT0173] Lee P , Chandel NS, Simon MC. Cellular adaptation to hypoxia through hypoxia inducible factors and beyond. Nat Rev Mol Cell Biol 2020;21:268–83. 32144406 10.1038/s41580-020-0227-y PMC7222024

[CIT0174] Leitch V , Agre P, King LS. Altered ubiquitination and stability of aquaporin-1 in hypertonic stress. Proc Natl Acad Sci USA 2001;98:2894–8. 11226337 10.1073/pnas.041616498 PMC30236

[CIT0175] Li C , Han T, Guo R et al. An integrative synthetic biology approach to interrogating cellular ubiquitin and ufm signaling. Int J Mol Sci2020a;21:4231. 32545848 10.3390/ijms21124231 PMC7352202

[CIT0176] Li C , Lu W, Yang L et al. MKRN3 regulates the epigenetic switch of mammalian puberty via ubiquitination of MBD3. Natl Sci Rev2020b;7:671–85. 34692086 10.1093/nsr/nwaa023 PMC8288866

[CIT0177] Li J , Cao F, Yin H-l et al. Ferroptosis: past, present and future. Cell Death Dis2020c;11:1–13. 32015325 10.1038/s41419-020-2298-2 PMC6997353

[CIT0178] Li C , Han T, Li Q et al. MKRN3-mediated ubiquitination of Poly (A)-binding proteins modulates the stability and translation of GNRH1 mRNA in mammalian puberty. Nucleic Acids Res 2021;49:3796–813. 33744966 10.1093/nar/gkab155 PMC8053111

[CIT0179] Liao Y , Sumara I, Pangou E. Non-proteolytic ubiquitylation in cellular signaling and human disease. Commun Biol 2022;5:1–15. 35136173 10.1038/s42003-022-03060-1 PMC8826416

[CIT0180] Linares LK , Hengstermann A, Ciechanover A et al. HdmX stimulates Hdm2-mediated ubiquitination and degradation of p53. Proc Natl Acad Sci USA 2003;100:12009–14. 14507994 10.1073/pnas.2030930100 PMC218704

[CIT0181] Liu Z , Chen P, Gao H et al. Ubiquitylation of autophagy receptor Optineurin by HACE1 activates selective autophagy for tumor suppression. Cancer Cell 2014;26:106–20. 25026213 10.1016/j.ccr.2014.05.015 PMC4166492

[CIT0182] Liu P , Gan W, Guo C et al. Akt-mediated phosphorylation of XLF impairs non-homologous end-joining DNA repair. Mol Cell 2015;57:648–61. 25661488 10.1016/j.molcel.2015.01.005 PMC4336609

[CIT0183] Liu J , Zhang C, Zhao Y et al. Parkin targets HIF-1α for ubiquitination and degradation to inhibit breast tumor progression. Nat Commun 2017;8:1–16. 29180628 10.1038/s41467-017-01947-w PMC5703960

[CIT0184] Liu L , Damerell DR, Koukouflis L et al. UbiHub: a data hub for the explorers of ubiquitination pathways. Bioinformatics2019a;35:2882–4. 30601939 10.1093/bioinformatics/bty1067 PMC6691330

[CIT0185] Liu T , Jiang L, Tavana O et al. The deubiquitylase OTUB1 mediates ferroptosis via stabilization of SLC7A11. Cancer Res2019b;79:1913–24. 30709928 10.1158/0008-5472.CAN-18-3037 PMC6467774

[CIT0186] Liu B , Ruan J, Chen M et al. Deubiquitinating enzymes (DUBs): decipher underlying basis of neurodegenerative diseases. Mol Psychiatry2022a;27:259–68. 34285347 10.1038/s41380-021-01233-8

[CIT0187] Liu J , Jin J, Liang T et al. To Ub or not to Ub: a regulatory question in TGF-β signaling. Trends Biochem Sci2022b;47:1059–72. 35810076 10.1016/j.tibs.2022.06.001

[CIT0188] Liu S , Jiang T, Bu F et al. Structural basis for BIRC6 to balance apoptosis and autophagy. bioRxiv2022c. doi:10.1101/2022.12.10.519866

[CIT0189] Lopata A , Kniss A, Löhr F et al. Ubiquitination in the ERAD process. Int J Mol Sci 2020;21:5369. 32731622 10.3390/ijms21155369 PMC7432864

[CIT0190] Lord CJ , Ashworth A. The DNA damage response and cancer therapy. Nature 2012;481:287–94. 22258607 10.1038/nature10760

[CIT0191] Lou Z , Minter-Dykhouse K, Franco S et al. MDC1 maintains genomic stability by participating in the amplification of ATM-dependent DNA damage signals. Mol Cell 2006;21:187–200. 16427009 10.1016/j.molcel.2005.11.025

[CIT0192] Lu X , Xu M, Zhu Q et al. RNF8-ubiquitinated KMT5A is required for RNF168-induced H2A ubiquitination in response to DNA damage. FASEB J 2021;35:e21326. 33710666 10.1096/fj.202002234R

[CIT0193] Luo K , Zhang H, Wang L et al. Sumoylation of MDC1 is important for proper DNA damage response. EMBO J 2012;31:3008–19. 22635276 10.1038/emboj.2012.158 PMC3395099

[CIT0194] Ma B , Chen Y, Chen L et al. Hypoxia regulates Hippo signalling through the SIAH2 ubiquitin E3 ligase. Nat Cell Biol 2015;17:95–103. 25438054 10.1038/ncb3073

[CIT0195] Mabbitt PD , Loreto A, Déry M-A et al. Structural basis for RING-Cys-Relay E3 ligase activity and its role in axon integrity. Nat Chem Biol 2020;16:1227–36. 32747811 10.1038/s41589-020-0598-6 PMC7610530

[CIT0196] MacGregor DR , Penfield S. Exploring the pleiotropy of hos1. J Exp Bot 2015;66:1661–71. 25697795 10.1093/jxb/erv022

[CIT0197] Maeoka Y , Okamoto T, Wu Y et al. Renal medullary tonicity regulates RNF183 expression in the collecting ducts via NFAT5. Biochem Biophys Res Commun2019a;514:436–42. 31053298 10.1016/j.bbrc.2019.04.168

[CIT0198] Maeoka Y , Wu Y, Okamoto T et al. NFAT5 up-regulates expression of the kidney-specific ubiquitin ligase gene Rnf183 under hypertonic conditions in inner-medullary collecting duct cells. J Biol Chem2019b;294:101–15. 30413537 10.1074/jbc.RA118.002896 PMC6322883

[CIT0199] Mailand N , Bekker-Jensen S, Bartek J et al. Destruction of Claspin by SCFβTrCP restrains Chk1 activation and facilitates recovery from genotoxic stress. Mol Cell 2006;23:307–18. 16885021 10.1016/j.molcel.2006.06.016

[CIT0200] Mailand N , Bekker-Jensen S, Faustrup H et al. RNF8 ubiquitylates histones at DNA double-strand breaks and promotes assembly of repair proteins. Cell 2007;131:887–900. 18001824 10.1016/j.cell.2007.09.040

[CIT0201] Mallette FA , Mattiroli F, Cui G et al. RNF8-and RNF168-dependent degradation of KDM4A/JMJD2A triggers 53BP1 recruitment to DNA damage sites. EMBO J 2012;31:1865–78. 22373579 10.1038/emboj.2012.47 PMC3343333

[CIT0202] Mandemaker I , van Cuijk L, Janssens RC et al. DNA damage-induced histone H1 ubiquitylation is mediated by HUWE1 and stimulates the RNF8-RNF168 pathway. Sci Rep 2017;7:1–11. 29127375 10.1038/s41598-017-15194-y PMC5681673

[CIT0203] Mansour MA. Ubiquitination: friend and foe in cancer. Int J Biochem Cell Biol 2018;101:80–93. 29864543 10.1016/j.biocel.2018.06.001

[CIT0204] Maréchal A , Li J-M, Ji XY et al. PRP19 transforms into a sensor of RPA-ssDNA after DNA damage and drives ATR activation via a ubiquitin-mediated circuitry. Mol Cell 2014;53:235–46. 24332808 10.1016/j.molcel.2013.11.002 PMC3946837

[CIT0205] Marei H , Tsai W-TK, Kee Y-S et al. Antibody targeting of E3 ubiquitin ligases for receptor degradation. Nature 2022;610:182–9. 36131013 10.1038/s41586-022-05235-6 PMC9534761

[CIT0206] Martinez A , Lectez B, Ramirez J et al. Quantitative proteomic analysis of Parkin substrates in Drosophila neurons. Mol Neurodegener 2017;12:1–19. 28399880 10.1186/s13024-017-0170-3 PMC5387213

[CIT0207] Matchkov VV , Krivoi II. Specialized functional diversity and interactions of the Na, K-ATPase. Front Physiol 2016;7:179. 27252653 10.3389/fphys.2016.00179 PMC4879863

[CIT0208] Mathur A , Pandey VK, Kakkar P. Activation of GSK3β/β-TrCP axis via PHLPP1 exacerbates Nrf2 degradation leading to impairment in cell survival pathway during diabetic nephropathy. Free Radic Biol Med 2018;120:414–24. 29655866 10.1016/j.freeradbiomed.2018.04.550

[CIT0209] Mattiroli F , Penengo L. Histone ubiquitination: an integrative signaling platform in genome stability. Trends Genet 2021;37:566–81. 33485674 10.1016/j.tig.2020.12.005

[CIT0210] Mattiroli F , Vissers JH, van Dijk WJ et al. RNF168 ubiquitinates K13-15 on H2A/H2AX to drive DNA damage signaling. Cell 2012;150:1182–95. 22980979 10.1016/j.cell.2012.08.005

[CIT0211] Maxwell PH , Wiesener MS, Chang G-W et al. The tumour suppressor protein VHL targets hypoxia-inducible factors for oxygen-dependent proteolysis. Nature 1999;399:271–5. 10353251 10.1038/20459

[CIT0212] Maxwell BA , Gwon Y, Mishra A et al. Ubiquitination is essential for recovery of cellular activities after heat shock. Science 2021;372:eabc3593. 34739326 10.1126/science.abc3593 PMC8574219

[CIT0213] McCabe JT , Burrell AS. Alterations of AP-1 and CREB protein DNA binding in rat supraoptic and paraventricular nuclei by acute and repeated hyperosmotic stress. Brain Res Bull 2001;55:347–58. 11489342 10.1016/s0361-9230(01)00520-2

[CIT0214] McClellan AJ , Laugesen SH, Ellgaard L. Cellular functions and molecular mechanisms of non-lysine ubiquitination. Open Biol 2019;9:190147. 31530095 10.1098/rsob.190147 PMC6769291

[CIT0215] McDonald FJ , Western AH, McNeil JD et al. Ubiquitin-protein ligase WWP2 binds to and downregulates the epithelial Na+ channel. Am J Physiol Renal Physiol 2002;283:F431–6. 12167593 10.1152/ajprenal.00080.2002

[CIT0216] Medicherla B , Goldberg AL. Heat shock and oxygen radicals stimulate ubiquitin-dependent degradation mainly of newly synthesized proteins. J Cell Biol 2008;182:663–73. 18725537 10.1083/jcb.200803022 PMC2518706

[CIT0217] Min J-h , Yang H, Ivan M et al. Structure of an HIF-1α-pVHL complex: hydroxyproline recognition in signaling. Science 2002;296:1886–9. 12004076 10.1126/science.1073440

[CIT0218] Mohr SM , Bagriantsev SN, Gracheva EO. Cellular, molecular, and physiological adaptations of hibernation: the solution to environmental challenges. Annu Rev Cell Dev Biol 2020;36:315–38. 32897760 10.1146/annurev-cellbio-012820-095945

[CIT0219] Møller P , Loft S, Lundby C et al. Acute hypoxia and hypoxic exercise induce DNA strand breaks and oxidative DNA damage in humans. FASEB J 2001;15:1181–6. 11344086 10.1096/fj.00-0703com

[CIT0220] Morett E , Bork P. A novel transactivation domain in parkin. Trends Biochem Sci 1999;24:229–31. 10366851 10.1016/s0968-0004(99)01381-x

[CIT0221] Morreale FE , Walden H. Types of ubiquitin ligases. Cell 2016;165:248–248.e1. 27015313 10.1016/j.cell.2016.03.003

[CIT0222] Moyal L , Lerenthal Y, Gana-Weisz M et al. Requirement of ATM-dependent monoubiquitylation of histone H2B for timely repair of DNA double-strand breaks. Mol Cell 2011;41:529–42. 21362549 10.1016/j.molcel.2011.02.015 PMC3397146

[CIT0223] Muz B , de la Puente P, Azab F et al. The role of hypoxia in cancer progression, angiogenesis, metastasis, and resistance to therapy. Hypoxia 2015;3:83. 27774485 10.2147/HP.S93413 PMC5045092

[CIT0224] Nakamura K , Kato A, Kobayashi J et al. Regulation of homologous recombination by RNF20-dependent H2B ubiquitination. Mol Cell 2011;41:515–28. 21362548 10.1016/j.molcel.2011.02.002

[CIT0225] Nakayama K , Frew IJ, Hagensen M et al. Siah2 regulates stability of prolyl-hydroxylases, controls HIF1α abundance, and modulates physiological responses to hypoxia. Cell 2004;117:941–52. 15210114 10.1016/j.cell.2004.06.001

[CIT0226] Nakayama K , Qi J, Ronai Z. The ubiquitin ligase Siah2 and the hypoxia response. Mol Cancer Res 2009;7:443–51. 19372575 10.1158/1541-7786.MCR-08-0458 PMC2860273

[CIT0227] Nasu J , Murakami K, Miyagawa S et al. E6AP ubiquitin ligase mediates ubiquitin-dependent degradation of peroxiredoxin 1. J Cell Biochem 2010;111:676–85. 20589759 10.1002/jcb.22752

[CIT0228] Neuhofer W. Role of NFAT5 in inflammatory disorders associated with osmotic stress. Curr Genomics 2010;11:584–90. 21629436 10.2174/138920210793360961 PMC3078683

[CIT0229] Nguyen AT , Prado MA, Schmidt PJ et al. UBE2O remodels the proteome during terminal erythroid differentiation. Science 2017;357:eaan0218. 28774900 10.1126/science.aan0218 PMC5812729

[CIT0230] Nguyen KT , Mun S-H, Yang J et al. The MARCHF6 E3 ubiquitin ligase acts as an NADPH sensor for the regulation of ferroptosis. Nat Cell Biol 2022;24:1239–51. 35941365 10.1038/s41556-022-00973-1

[CIT0231] Ning S , Luo L, Yu B et al. Structures, functions, and inhibitors of LUBAC and its related diseases. J Leukoc Biol 2022;112:799–811. 35266190 10.1002/JLB.3MR0222-508R

[CIT0232] Nishiyama H , Itoh K, Kaneko Y et al. A glycine-rich RNA-binding protein mediating cold-inducible suppression of mammalian cell growth. J Cell Biol 1997;137:899–908. 9151692 10.1083/jcb.137.4.899 PMC2139845

[CIT0233] Nowsheen S , Aziz K, Aziz A et al. L3MBTL2 orchestrates ubiquitin signalling by dictating the sequential recruitment of RNF8 and RNF168 after DNA damage. Nat Cell Biol 2018;20:455–64. 29581593 10.1038/s41556-018-0071-x PMC6083879

[CIT0234] Nucifora FC , Nucifora LG, Ng C-H et al. Ubiqutination via K27 and K29 chains signals aggregation and neuronal protection of LRRK2 by WSB1. Nat Commun 2016;7:1–11. 10.1038/ncomms11792 PMC4899630 27273569

[CIT0235] Oakes SA , Papa FR. The role of endoplasmic reticulum stress in human pathology. Ann Rev Pathol 2015;10:173–94. 25387057 10.1146/annurev-pathol-012513-104649 PMC5568783

[CIT0236] Ochi T , Blackford AN, Coates J et al. PAXX, a paralog of XRCC4 and XLF, interacts with Ku to promote DNA double-strand break repair. Science 2015;347:185–8. 25574025 10.1126/science.1261971 PMC4338599

[CIT0237] Ohh M , Park CW, Ivan M et al. Ubiquitination of hypoxia-inducible factor requires direct binding to the β-domain of the von Hippel–Lindau protein. Nat Cell Biol 2000;2:423–7. 10878807 10.1038/35017054

[CIT0238] Okamoto T , Wu Y, Matsuhisa K et al. Hypertonicity-responsive ubiquitin ligase RNF183 promotes Na, K-ATPase lysosomal degradation through ubiquitination of its β1 subunit. Biochem Biophys Res Commun 2020;521:1030–5. 31732153 10.1016/j.bbrc.2019.11.001

[CIT0239] Okatsu K , Oka T, Iguchi M et al. PINK1 autophosphorylation upon membrane potential dissipation is essential for Parkin recruitment to damaged mitochondria. Nat Commun 2012;3:1016. 22910362 10.1038/ncomms2016 PMC3432468

[CIT0240] Olsen SK , Lima CD. Structure of a ubiquitin E1-E2 complex: insights to E1-E2 thioester transfer. Mol Cell 2013;49:884–96. 23416107 10.1016/j.molcel.2013.01.013 PMC3625138

[CIT0241] Ong G , Logue SE. Unfolding the interactions between endoplasmic reticulum stress and oxidative stress. Antioxidants 2023;12:981. 37237847 10.3390/antiox12050981 PMC10215201

[CIT0242] Onishi M , Yamano K, Sato M et al. Molecular mechanisms and physiological functions of mitophagy. EMBO J 2021;40:e104705. 33438778 10.15252/embj.2020104705 PMC7849173

[CIT0243] Ordureau A , Sarraf SA, Duda DM et al. Quantitative proteomics reveal a feedforward mechanism for mitochondrial PARKIN translocation and ubiquitin chain synthesis. Mol Cell 2014;56:360–75. 25284222 10.1016/j.molcel.2014.09.007 PMC4254048

[CIT0244] Orthwein A , Noordermeer SM, Wilson MD et al. A mechanism for the suppression of homologous recombination in G1 cells. Nature 2015;528:422–6. 26649820 10.1038/nature16142 PMC4880051

[CIT0245] Otten EG , Werner E, Crespillo-Casado A et al. Ubiquitylation of lipopolysaccharide by RNF213 during bacterial infection. Nature 2021;594:111–6. 34012115 10.1038/s41586-021-03566-4 PMC7610904

[CIT0246] Paltoglou S , Roberts B. HIF-1α and EPAS ubiquitination mediated by the VHL tumour suppressor involves flexibility in the ubiquitination mechanism, similar to other RING E3 ligases. Oncogene 2007;26:604–9. 16862177 10.1038/sj.onc.1209818

[CIT0247] Pan F , Zarate J, Bradley TM. A homolog of the E3 ubiquitin ligase Rbx1 is induced during hyperosmotic stress of salmon. Am J Physiol Regul Integr Comp Physiol 2002;282:R1643–53. 12010746 10.1152/ajpregu.00571.2001

[CIT0248] Pao K-C , Wood NT, Knebel A et al. Activity-based E3 ligase profiling uncovers an E3 ligase with esterification activity. Nature 2018;556:381–5. 29643511 10.1038/s41586-018-0026-1

[CIT0249] Parag HA , Raboy B, Kulka RG. Effect of heat shock on protein degradation in mammalian cells: involvement of the ubiquitin system. EMBO J 1987;6:55–61. 3034579 10.1002/j.1460-2075.1987.tb04718.x PMC553356

[CIT0250] Park C-W , Ryu K-Y. Cellular ubiquitin pool dynamics and homeostasis. BMB Rep 2014;47:475–82. 24924398 10.5483/BMBRep.2014.47.9.128 PMC4206721

[CIT0251] Paull TT , Gellert M. The 3ʹ to 5ʹ exonuclease activity of Mre11 facilitates repair of DNA double-strand breaks. Mol Cell 1998;1:969–79. 9651580 10.1016/s1097-2765(00)80097-0

[CIT0252] Peng H , Yang J, Li G et al. Ubiquitylation of p62/sequestosome1 activates its autophagy receptor function and controls selective autophagy upon ubiquitin stress. Cell Res 2017;27:657–74. 28322253 10.1038/cr.2017.40 PMC5520855

[CIT0253] Peng H , Yang F, Hu Q et al. The ubiquitin-specific protease USP8 directly deubiquitinates SQSTM1/p62 to suppress its autophagic activity. Autophagy 2020;16:698–708. 31241013 10.1080/15548627.2019.1635381 PMC7138243

[CIT0254] Peschiaroli A , Dorrello NV, Guardavaccaro D et al. SCFβTrCP-mediated degradation of Claspin regulates recovery from the DNA replication checkpoint response. Mol Cell 2006;23:319–29. 16885022 10.1016/j.molcel.2006.06.013

[CIT0255] Petrone P , Asensio JA, Marini CP. Management of accidental hypothermia and cold injury. Curr Probl Surg 2014;51:417–31. 25242454 10.1067/j.cpsurg.2014.07.004

[CIT0256] Petroski MD , Deshaies RJ. Function and regulation of cullin–RING ubiquitin ligases. Nat Rev Mol Cell Biol 2005;6:9–20. 15688063 10.1038/nrm1547

[CIT0257] Petrucelli L , Dickson D, Kehoe K et al. CHIP and Hsp70 regulate tau ubiquitination, degradation and aggregation. Hum Mol Genet 2004;13:703–14. 14962978 10.1093/hmg/ddh083

[CIT0258] Pickart CM. Mechanisms underlying ubiquitination. Annu Rev Biochem 2001;70:503–33. 11395416 10.1146/annurev.biochem.70.1.503

[CIT0259] Pickrell AM , Youle RJ. The roles of PINK1, parkin, and mitochondrial fidelity in Parkinson’s disease. Neuron 2015;85:257–73. 25611507 10.1016/j.neuron.2014.12.007 PMC4764997

[CIT0260] Pietrucha B , Heropolitańska-Pliszka E, Geffers R et al. Clinical and biological manifestation of RNF168 deficiency in two polish siblings. Front Immunol 2017;8:1683. 29255463 10.3389/fimmu.2017.01683 PMC5722808

[CIT0261] Podhorecka M , Skladanowski A, Bozko P. H2AX phosphorylation: its role in DNA damage response and cancer therapy. J Nucleic Acids 2010;2010:920161. 20811597 10.4061/2010/920161 PMC2929501

[CIT0262] Pohl C , Dikic I. Cellular quality control by the ubiquitin-proteasome system and autophagy. Science 2019;366:818–22. 31727826 10.1126/science.aax3769

[CIT0263] Popovic D , Vucic D, Dikic I. Ubiquitination in disease pathogenesis and treatment. Nat Med 2014;20:1242–53. 25375928 10.1038/nm.3739

[CIT0264] Postow L , Funabiki H. An SCF complex containing Fbxl12 mediates DNA damage-induced Ku80 ubiquitylation. Cell Cycle 2013;12:587–95. 23324393 10.4161/cc.23408 PMC3594259

[CIT0265] Prajapati P , Gohel D, Shinde A et al. TRIM32 regulates mitochondrial mediated ROS levels and sensitizes the oxidative stress induced cell death. Cell Signal 2020;76:109777. 32918979 10.1016/j.cellsig.2020.109777

[CIT0266] Qian S-B , McDonough H, Boellmann F et al. CHIP-mediated stress recovery by sequential ubiquitination of substrates and Hsp70. Nature 2006;440:551–5. 16554822 10.1038/nature04600 PMC4112096

[CIT0267] Qiu J , Sheedlo MJ, Yu K et al. Ubiquitination independent of E1 and E2 enzymes by bacterial effectors. Nature 2016;533:120–4. 27049943 10.1038/nature17657 PMC4905768

[CIT0268] Qiu J , Yu K, Fei X et al. A unique deubiquitinase that deconjugates phosphoribosyl-linked protein ubiquitination. Cell Res 2017;27:865–81. 28497808 10.1038/cr.2017.66 PMC5518988

[CIT0269] Rachakonda G , Xiong Y, Sekhar KR et al. Covalent modification at Cys151 dissociates the electrophile sensor Keap1 from the ubiquitin ligase CUL3. Chem Res Toxicol 2008;21:705–10. 18251510 10.1021/tx700302s

[CIT0270] Ranjan K , Hedl M, Sinha S et al. Ubiquitination of ATF6 by disease-associated RNF186 promotes the innate receptor-induced unfolded protein response. J Clin Invest 2021;131:e145472. 34623328 10.1172/JCI145472 PMC8409591

[CIT0271] Rape M. Ubiquitylation at the crossroads of development and disease. Nat Rev Mol Cell Biol 2018;19:59–70. 28928488 10.1038/nrm.2017.83

[CIT0272] Reiter KH , Klevit RE. Characterization of RING-between-RING E3 ubiquitin transfer mechanisms. In: Mayor T, Kleiger G (eds.) The Ubiquitin Proteasome System. New York: Springer, 2018, 3–17. 10.1007/978-1-4939-8706-1_1 30242699

[CIT0273] Rennie ML , Chaugule VK, Walden H. Modes of allosteric regulation of the ubiquitination machinery. Curr Opin Struct Biol 2020;62:189–96. 32305021 10.1016/j.sbi.2020.02.003

[CIT0274] Richter K , Haslbeck M, Buchner J. The heat shock response: life on the verge of death. Mol Cell 2010;40:253–66. 20965420 10.1016/j.molcel.2010.10.006

[CIT0275] Rotin D , Kumar S. Physiological functions of the HECT family of ubiquitin ligases. Nat Rev Mol Cell Biol 2009;10:398–409. 19436320 10.1038/nrm2690

[CIT0276] Sadowska A , Kameda T, Krupkova O et al. Osmosensing, osmosignalling and inflammation: how intervertebral disc cells respond to altered osmolarity. Eur Cells Mater 2018;36:231–50. 10.22203/eCM.v036a17 30452080

[CIT0277] Sakamaki J-i , Ode KL, Kurikawa Y et al. Ubiquitination of phosphatidylethanolamine in organellar membranes. Mol Cell 2022;82:3677–3692.e11. 36044902 10.1016/j.molcel.2022.08.008

[CIT0278] Sakamoto KM , Kim KB, Kumagai A et al. Protacs: chimeric molecules that target proteins to the Skp1–Cullin–F box complex for ubiquitination and degradation. Proc Natl Acad Sci USA 2001;98:8554–9. 11438690 10.1073/pnas.141230798 PMC37474

[CIT0279] Sánchez-Tena S , Cubillos-Rojas M, Schneider T et al. Functional and pathological relevance of HERC family proteins: a decade later. Cell Mol Life Sci 2016;73:1955–68. 26801221 10.1007/s00018-016-2139-8 PMC11108380

[CIT0280] Sano R , Reed JC. ER stress-induced cell death mechanisms. Biochim Biophys Acta, Mol Cell Res 2013;1833:3460–70. 10.1016/j.bbamcr.2013.06.028 PMC3834229 23850759

[CIT0281] Schmidt CK , Galanty Y, Sczaniecka-Clift M et al. Systematic E2 screening reveals a UBE2D–RNF138–CtIP axis promoting DNA repair. Nat Cell Biol 2015;17:1458–70. 26502057 10.1038/ncb3260 PMC4894550

[CIT0282] Schneider M , Radoux CJ, Hercules A et al. The PROTACtable genome. Nat Rev Drug Discovery 2021;20:789–97. 34285415 10.1038/s41573-021-00245-x

[CIT0283] Schödel J , Ratcliffe PJ. Mechanisms of hypoxia signalling: new implications for nephrology. Nat Rev Nephrol 2019;15:641–59. 31488900 10.1038/s41581-019-0182-z

[CIT0284] Schofield JH , Schafer ZT. Mitochondrial reactive oxygen species and mitophagy: a complex and nuanced relationship. Antioxid Redox Signal 2021;34:517–30. 32079408 10.1089/ars.2020.8058

[CIT0285] Schulman BA , Wade Harper J. Ubiquitin-like protein activation by E1 enzymes: the apex for downstream signalling pathways. Nat Rev Mol Cell Biol 2009;10:319–31. 19352404 10.1038/nrm2673 PMC2712597

[CIT0286] Schwake M , Friedrich T, Jentsch TJ. An internalization signal in ClC-5, an endosomal Cl^−^ channel mutated in Dent’s disease. J Biol Chem 2001;276:12049–54. 11116157 10.1074/jbc.M010642200

[CIT0287] Sheen MR , Kim J-A, Lim SW et al. Interstitial tonicity controls TonEBP expression in the renal medulla. Kidney Int 2009;75:518–25. 19052532 10.1038/ki.2008.601

[CIT0288] Shen Y , Liu W, Zhang X et al. TRAF3 promotes ROS production and pyroptosis by targeting ULK1 ubiquitination in macrophages. FASEB J 2020;34:7144–159. 32275117 10.1096/fj.201903073R

[CIT0289] Sheng X , You Q, Zhu H et al. Bacterial effector NleL promotes enterohemorrhagic *E. coli*-induced attaching and effacing lesions by ubiquitylating and inactivating JNK. PLoS Pathog 2017;13:e1006534. 28753655 10.1371/journal.ppat.1006534 PMC5549993

[CIT0290] Sheng X , You Q, Zhu H et al. Enterohemorrhagic *E. coli* effector NleL disrupts host NF-κB signaling by targeting multiple host proteins. J Mol Cell Biol 2020;12:318–21. 32065237 10.1093/jmcb/mjaa003 PMC7232126

[CIT0291] Shi W , Ma Z, Willers H et al. Disassembly of MDC1 foci is controlled by ubiquitin-proteasome-dependent degradation. J Biol Chem 2008;283:31608–16. 18757370 10.1074/jbc.M801082200

[CIT0292] Shin D , Mukherjee R, Liu Y et al. Regulation of phosphoribosyl-linked serine ubiquitination by deubiquitinases DupA and DupB. Mol Cell 2020;77:164–179.e6. 31732457 10.1016/j.molcel.2019.10.019 PMC6941232

[CIT0293] Shirasaki R , Matthews GM, Gandolfi S et al. Functional genomics identify distinct and overlapping genes mediating resistance to different classes of heterobifunctional degraders of oncoproteins. Cell Rep 2021;34:108532. 33406420 10.1016/j.celrep.2020.108532 PMC8485877

[CIT0294] Siednienko J , Jackson R, Mellett M et al. Pellino3 targets the IRF7 pathway and facilitates autoregulation of TLR3-and viral-induced expression of type I interferons. Nat Immunol 2012;13:1055–62. 23042151 10.1038/ni.2429

[CIT0295] Sies H , Berndt C, Jones DP. Oxidative stress. Annu Rev Biochem 2017;86:715–48. 28441057 10.1146/annurev-biochem-061516-045037

[CIT0296] Singh S , Ng J, Sivaraman J. Exploring the “Other” subfamily of HECT E3-ligases for therapeutic intervention. Pharmacol Ther 2021;224:107809. 33607149 10.1016/j.pharmthera.2021.107809

[CIT0297] So CC , Ramachandran S, Martin A. E3 ubiquitin ligases RNF20 and RNF40 are required for double-stranded break (DSB) repair: evidence for monoubiquitination of histone H2B lysine 120 as a novel axis of DSB signaling and repair. Mol Cell Biol 2019;39:e00488–18. 30692271 10.1128/MCB.00488-18 PMC6447412

[CIT0298] Sobhian B , Shao G, Lilli DR et al. RAP80 targets BRCA1 to specific ubiquitin structures at DNA damage sites. Science 2007;316:1198–202. 17525341 10.1126/science.1139516 PMC2706583

[CIT0299] Sonna LA , Fujita J, Gaffin SL et al. Invited review: effects of heat and cold stress on mammalian gene expression. J Appl Physiol 2002;92:1725–42. 11896043 10.1152/japplphysiol.01143.2001

[CIT0300] Soss SE , Rose KL, Hill S et al. Biochemical and proteomic analysis of ubiquitination of Hsc70 and Hsp70 by the E3 ligase CHIP. PLoS One 2015;10:e0128240. 26010904 10.1371/journal.pone.0128240 PMC4444009

[CIT0301] Spit M , Rieser E, Walczak H. Linear ubiquitination at a glance. J Cell Sci 2019;132:jcs208512. 30659056 10.1242/jcs.208512

[CIT0302] Spriggs KA , Bushell M, Willis AE. Translational regulation of gene expression during conditions of cell stress. Mol Cell 2010;40:228–37. 20965418 10.1016/j.molcel.2010.09.028

[CIT0303] Squair DR , Virdee S. A new dawn beyond lysine ubiquitination. Nat Chem Biol 2022;18:802–11. 35896829 10.1038/s41589-022-01088-2

[CIT0304] Staub O , Dho S, Henry P et al. WW domains of Nedd4 bind to the proline-rich PY motifs in the epithelial Na+ channel deleted in Liddle’s syndrome. EMBO J 1996;15:2371–80. 8665844 PMC450167

[CIT0305] Staub O , Gautschi I, Ishikawa T et al. Regulation of stability and function of the epithelial Na+ channel (ENaC) by ubiquitination. EMBO J 1997;16:6325–36. 9351815 10.1093/emboj/16.21.6325 PMC1170239

[CIT0306] Stewart GS , Panier S, Townsend K et al. The RIDDLE syndrome protein mediates a ubiquitin-dependent signaling cascade at sites of DNA damage. Cell 2009;136:420–34. 19203578 10.1016/j.cell.2008.12.042

[CIT0307] Stewart MD , Ritterhoff T, Klevit RE et al. E2 enzymes: more than just middle men. Cell Res 2016;26:423–40. 27002219 10.1038/cr.2016.35 PMC4822130

[CIT0308] Stucki M , Clapperton JA, Mohammad D et al. MDC1 directly binds phosphorylated histone H2AX to regulate cellular responses to DNA double-strand breaks. Cell 2005;123:1213–26. 16377563 10.1016/j.cell.2005.09.038

[CIT0309] Sun H , Li X-B, Meng Y et al. TRAF6 upregulates expression of HIF-1α and promotes tumor angiogenesis. Cancer Res 2013;73:4950–9. 23722539 10.1158/0008-5472.CAN-13-0370

[CIT0310] Sun S , Shi G, Sha H et al. IRE1α is an endogenous substrate of endoplasmic-reticulum-associated degradation. Nat Cell Biol 2015;17:1546–55. 26551274 10.1038/ncb3266 PMC4670240

[CIT0311] Sun H , Wei G, Liu H et al. Inhibition of XBP1s ubiquitination enhances its protein stability and improves glucose homeostasis. Metabolism 2020;105:154046. 31837300 10.1016/j.metabol.2019.154046

[CIT0312] Sun X , Huang N, Li P et al. TRIM21 ubiquitylates GPX4 and promotes ferroptosis to aggravate ischemia/reperfusion-induced acute kidney injury. Life Sci 2023;321:121608. 36958437 10.1016/j.lfs.2023.121608 PMC11483487

[CIT0313] Swatek KN , Komander D. Ubiquitin modifications. Cell Res 2016;26:399–422. 27012465 10.1038/cr.2016.39 PMC4822133

[CIT0314] Sy SM , Huen MS, Chen J. PALB2 is an integral component of the BRCA complex required for homologous recombination repair. Proc Natl Acad Sci USA 2009;106:7155–60. 19369211 10.1073/pnas.0811159106 PMC2678481

[CIT0315] Takeda K , Nagashima S, Shiiba I et al. MITOL prevents ER stress-induced apoptosis by IRE 1α ubiquitylation at ER–mitochondria contact sites. EMBO J 2019;38:e100999. 31368599 10.15252/embj.2018100999 PMC6669929

[CIT0316] Tang H , Jiang X, Hua Y et al. NEDD4L facilitates granulosa cell ferroptosis by promoting GPX4 ubiquitination and degradation. Endocr Connect 2023;12:e220459. 36662677 10.1530/EC-22-0459 PMC10083675

[CIT0317] Tank EM , True HL. Disease-associated mutant ubiquitin causes proteasomal impairment and enhances the toxicity of protein aggregates. PLoS Genet 2009;5:e1000382. 19214209 10.1371/journal.pgen.1000382 PMC2633047

[CIT0318] Thorslund T , Ripplinger A, Hoffmann S et al. Histone H1 couples initiation and amplification of ubiquitin signalling after DNA damage. Nature 2015;527:389–93. 26503038 10.1038/nature15401

[CIT0319] Toure M , Crews CM. Small-molecule PROTACS: new approaches to protein degradation. Angew Chem Int Ed 2016;55:1966–73. 10.1002/anie.201507978 26756721

[CIT0320] Toyofuku T , Okamoto Y, Ishikawa T et al. LRRK 2 regulates endoplasmic reticulum–mitochondrial tethering through the PERK-mediated ubiquitination pathway. EMBO J 2020;39:e100875. 31821596 10.15252/embj.2018100875 PMC6960452

[CIT0321] Trempe J-F , Sauvé V, Grenier K et al. Structure of Parkin reveals mechanisms for ubiquitin ligase activation. Science 2013;340:1451–5. 23661642 10.1126/science.1237908

[CIT0322] Trulsson F , Akimov V, Robu M et al. Deubiquitinating enzymes and the proteasome regulate preferential sets of ubiquitin substrates. Nat Commun 2022;13:1–17. 35585066 10.1038/s41467-022-30376-7 PMC9117253

[CIT0323] Uckelmann M , Sixma TK. Histone ubiquitination in the DNA damage response. DNA Repair (Amst) 2017;56:92–101. 28624371 10.1016/j.dnarep.2017.06.011

[CIT0324] Ui A , Nagaura Y, Yasui A. Transcriptional elongation factor ENL phosphorylated by ATM recruits polycomb and switches off transcription for DSB repair. Mol Cell 2015;58:468–82. 25921070 10.1016/j.molcel.2015.03.023

[CIT0325] Van Der Reijden BA , Erpelinck-Verschueren CA, Löwenberg B et al. TRIADs: a new class of proteins with a novel cysteine-rich signature. Protein Sci 1999;8:1557–61. 10422847 10.1110/ps.8.7.1557 PMC2144383

[CIT0326] Van Leeuwen FW , de Kleijn DP, van den Hurk HH et al. Frameshift mutants of β amyloid precursor protein and ubiquitin-B in Alzheimer’s and Down patients. Science 1998;279:242–7. 9422699 10.1126/science.279.5348.242

[CIT0327] Varshavsky A. The N-end rule pathway and regulation by proteolysis. Protein Sci 2011;20:1298–345. 21633985 10.1002/pro.666 PMC3189519

[CIT0328] Vijay-Kumar S , Bugg CE, Cook WJ. Structure of ubiquitin refined at 1.8 Å resolution. J Mol Biol 1987;194:531–44. 3041007 10.1016/0022-2836(87)90679-6

[CIT0329] Walser F , Mulder MP, Bragantini B et al. Ubiquitin phosphorylation at Thr12 modulates the DNA damage response. Mol Cell 2020;80:423–436.e9. 33022275 10.1016/j.molcel.2020.09.017 PMC7655664

[CIT0330] Walters B , Campbell S, Chen P et al. Differential effects of Usp14 and Uch-L1 on the ubiquitin proteasome system and synaptic activity. Mol Cell Neurosci 2008;39:539–48. 18771733 10.1016/j.mcn.2008.07.028 PMC2734958

[CIT0331] Wan M , Sulpizio AG, Akturk A et al. Deubiquitination of phosphoribosyl-ubiquitin conjugates by phosphodiesterase-domain–containing Legionella effectors. Proc Natl Acad Sci USA 2019;116:23518–26. 31690664 10.1073/pnas.1916287116 PMC6876201

[CIT0332] Wang B , Elledge SJ. Ubc13/Rnf8 ubiquitin ligases control foci formation of the Rap80/Abraxas/Brca1/Brcc36 complex in response to DNA damage. Proc Natl Acad Sci USA 2007;104:20759–63. 18077395 10.1073/pnas.0710061104 PMC2410075

[CIT0333] Wang GL , Jiang B-H, Rue EA et al. Hypoxia-inducible factor 1 is a basic-helix-loop-helix-PAS heterodimer regulated by cellular O2 tension. Proc Natl Acad Sci USA 1995;92:5510–4. 7539918 10.1073/pnas.92.12.5510 PMC41725

[CIT0334] Wang H , Zhai L, Xu J et al. Histone H3 and H4 ubiquitylation by the CUL4-DDB-ROC1 ubiquitin ligase facilitates cellular response to DNA damage. Mol Cell 2006;22:383–94. 16678110 10.1016/j.molcel.2006.03.035

[CIT0335] Wang B , Matsuoka S, Ballif BA et al. Abraxas and RAP80 form a BRCA1 protein complex required for the DNA damage response. Science 2007;316:1194–8. 17525340 10.1126/science.1139476 PMC3573690

[CIT0336] Wang Y , Yang C, Nahla Abdalla Hassan E et al. HO-1 reduces heat stress-induced apoptosis in bovine granulosa cells by suppressing oxidative stress. Aging (Albany NY) 2019;11:5535. 31404912 10.18632/aging.102136 PMC6710052

[CIT0337] Wang P , Dai X, Jiang W et al. RBR E3 ubiquitin ligases in tumorigenesis. Semin Cancer Biol2020a;67:131–44. 32442483 10.1016/j.semcancer.2020.05.002

[CIT0338] Wang Y , Argiles-Castillo D, Kane EI et al. HECT E3 ubiquitin ligases–emerging insights into their biological roles and disease relevance. J Cell Sci2020b;133:jcs228072. 32265230 10.1242/jcs.228072 PMC7157599

[CIT0339] Wang H-P , Chen W-J, Shen J-M et al. Attenuating glucose metabolism by Fbxw7 promotes Taxol sensitivity of colon cancer cells through downregulating NADPH oxidase 1 (Nox1). Ann Trans Med 2021;9:886. 10.21037/atm-21-2076 PMC8184419 34164520

[CIT0340] Wang C-C , Peng H, Wang Z et al. TRIM72-mediated degradation of the short form of p62/SQSTM1 rheostatically controls selective autophagy in human cells. Mil Med Res2022a;9:1–4. 35733226 10.1186/s40779-022-00392-1 PMC9215040

[CIT0341] Wang Y-T , Liu T-Y, Shen C-H et al. K48/K63-linked polyubiquitination of ATG9A by TRAF6 E3 ligase regulates oxidative stress-induced autophagy. Cell Rep2022b;38:110354. 35196483 10.1016/j.celrep.2022.110354

[CIT0342] Wang Y , Wang S, Zhang W. HRD1 functions as a tumor suppressor in ovarian cancer by facilitating ubiquitination-dependent SLC7A11 degradation. Cell Cycle 2023;22:1116–26. 36809917 10.1080/15384101.2023.2178102 PMC10081055

[CIT0343] Weber J , Polo S, Maspero E. HECT E3 ligases: a tale with multiple facets. Front Physiol 2019;10:370. 31001145 10.3389/fphys.2019.00370 PMC6457168

[CIT0344] Wei P , Pan D, Mao C et al. RNF34 is a cold-regulated E3 ubiquitin ligase for PGC-1α and modulates brown fat cell metabolism. Mol Cell Biol 2012;32:266–75. 22064484 10.1128/MCB.05674-11 PMC3255768

[CIT0345] Weissman AM. Themes and variations on ubiquitylation. Nat Rev Mol Cell Biol 2001;2:169–78. 11265246 10.1038/35056563

[CIT0346] Wilson MD , Benlekbir S, Fradet-Turcotte A et al. The structural basis of modified nucleosome recognition by 53BP1. Nature 2016;536:100–3. 27462807 10.1038/nature18951

[CIT0347] Wolyniec K , Levav-Cohen Y, Jiang Y et al. The E6AP E3 ubiquitin ligase regulates the cellular response to oxidative stress. Oncogene 2013;32:3510–9. 22986523 10.1038/onc.2012.365

[CIT0348] Wu X , Rapoport TA. Mechanistic insights into ER-associated protein degradation. Curr Opin Cell Biol 2018;53:22–8. 29719269 10.1016/j.ceb.2018.04.004 PMC6131047

[CIT0349] Wu D , Rastinejad F. Structural characterization of mammalian bHLH-PAS transcription factors. Curr Opin Struct Biol 2017;43:1–9. 27721191 10.1016/j.sbi.2016.09.011 PMC5382129

[CIT0350] Wu W , Sato K, Koike A et al. HERC2 is an E3 ligase that targets BRCA1 for degradation. Cancer Res 2010;70:6384–92. 20631078 10.1158/0008-5472.CAN-10-1304

[CIT0351] Wu J , Zhang X, Zhang L et al. Skp2 E3 ligase integrates ATM activation and homologous recombination repair by ubiquitinating NBS1. Mol Cell 2012;46:351–61. 22464731 10.1016/j.molcel.2012.02.018 PMC3518281

[CIT0352] Wu Y , Jiao H, Yue Y et al. Ubiquitin ligase E3 HUWE1/MULE targets transferrin receptor for degradation and suppresses ferroptosis in acute liver injury. Cell Death Differ 2022;29:1705–18. 35260822 10.1038/s41418-022-00957-6 PMC9433446

[CIT0353] Wu-Baer F , Lagrazon K, Yuan W et al. The BRCA1/BARD1 heterodimer assembles polyubiquitin chains through an unconventional linkage involving lysine residue K6 of ubiquitin. J Biol Chem 2003;278:34743–6. 12890688 10.1074/jbc.C300249200

[CIT0354] Xia Y , Wang J, Xu S et al. MEKK1 mediates the ubiquitination and degradation of c-Jun in response to osmotic stress. Mol Cell Biol 2007;27:510–7. 17101801 10.1128/MCB.01355-06 PMC1800814

[CIT0355] Xie Y , Hou W, Song X et al. Ferroptosis: process and function. Cell Death Differ 2016;23:369–79. 26794443 10.1038/cdd.2015.158 PMC5072448

[CIT0356] Xu P , Duong DM, Seyfried NT et al. Quantitative proteomics reveals the function of unconventional ubiquitin chains in proteasomal degradation. Cell 2009;137:133–45. 19345192 10.1016/j.cell.2009.01.041 PMC2668214

[CIT0357] Yan Q , Dutt S, Xu R et al. BBAP monoubiquitylates histone H4 at lysine 91 and selectively modulates the DNA damage response. Mol Cell 2009;36:110–20. 19818714 10.1016/j.molcel.2009.08.019 PMC2913878

[CIT0358] Yanagitani K , Juszkiewicz S, Hegde RS. UBE2O is a quality control factor for orphans of multiprotein complexes. Science 2017;357:472–5. 28774922 10.1126/science.aan0178 PMC5549844

[CIT0359] Yang S , Wang B, Humphries F et al. The E3 ubiquitin ligase Pellino3 protects against obesity-induced inflammation and insulin resistance. Immunity 2014;41:973–87. 25526310 10.1016/j.immuni.2014.11.013

[CIT0360] Yang Y , Luo M, Zhang K et al. Nedd4 ubiquitylates VDAC2/3 to suppress erastin-induced ferroptosis in melanoma. Nat Commun 2020;11:433. 31974380 10.1038/s41467-020-14324-x PMC6978386

[CIT0361] Yang E , Huang S, Jami-Alahmadi Y et al. Elucidation of TRIM25 ubiquitination targets involved in diverse cellular and antiviral processes. PLoS Pathog 2022;18:e1010743. 36067236 10.1371/journal.ppat.1010743 PMC9481182

[CIT0362] Yasuda S , Tsuchiya H, Kaiho A et al. Stress- and ubiquitylation-dependent phase separation of the proteasome. Nature 2020;578:296–300. 32025036 10.1038/s41586-020-1982-9

[CIT0363] Ye Y , Rape M. Building ubiquitin chains: E2 enzymes at work. Nat Rev Mol Cell Biol 2009;10:755–64. 19851334 10.1038/nrm2780 PMC3107738

[CIT0364] Yin Y , Seifert A, Chua JS et al. SUMO-targeted ubiquitin E3 ligase RNF4 is required for the response of human cells to DNA damage. Genes Dev 2012;26:1196–208. 22661230 10.1101/gad.189274.112 PMC3371408

[CIT0365] Yonashiro R , Sugiura A, Miyachi M et al. Mitochondrial ubiquitin ligase MITOL ubiquitinates mutant SOD1 and attenuates mutant SOD1-induced reactive oxygen species generation. Mol Biol Cell 2009;20:4524–30. 19741096 10.1091/mbc.E09-02-0112 PMC2770940

[CIT0366] Youle RJ , Narendra DP. Mechanisms of mitophagy. Nat Rev Mol Cell Biol 2011;12:9–14. 21179058 10.1038/nrm3028 PMC4780047

[CIT0367] Yu X , Fu S, Lai M et al. BRCA1 ubiquitinates its phosphorylation-dependent binding partner CtIP. Genes Dev 2006;20:1721–6. 16818604 10.1101/gad.1431006 PMC1522068

[CIT0368] Yuan J , Lv T, Yang J et al. HDLBP-stabilized lncFAL inhibits ferroptosis vulnerability by diminishing Trim69-dependent FSP1 degradation in hepatocellular carcinoma. Redox Biol 2022;58:102546. 36423520 10.1016/j.redox.2022.102546 PMC9692041

[CIT0369] Zeng M , Xiong Y, Safaee N et al. Exploring targeted degradation strategy for oncogenic KRASG12C. Cell Chem Biol 2020;27:19–31.e6. 31883964 10.1016/j.chembiol.2019.12.006

[CIT0370] Zhang F , Ma J, Wu J et al. PALB2 links BRCA1 and BRCA2 in the DNA-damage response. Curr Biol 2009;19:524–9. 19268590 10.1016/j.cub.2009.02.018 PMC2750839

[CIT0371] Zhang H , Amick J, Chakravarti R et al. A bipartite interaction between Hsp70 and CHIP regulates ubiquitination of chaperoned client proteins. Structure 2015;23:472–82. 25684577 10.1016/j.str.2015.01.003 PMC4351142

[CIT0372] Zhang Q , Karnak D, Tan M et al. FBXW7 facilitates nonhomologous end-joining via K63-linked polyubiquitylation of XRCC4. Mol Cell 2016;61:419–33. 26774286 10.1016/j.molcel.2015.12.010 PMC4744117

[CIT0373] Zhang W , Jiang B, Liu Y et al. Bufotalin induces ferroptosis in non-small cell lung cancer cells by facilitating the ubiquitination and degradation of GPX4. Free Radic Biol Med 2022;180:75–84. 35038550 10.1016/j.freeradbiomed.2022.01.009

[CIT0374] Zhang J , Xie H, Yao J et al. TRIM59 promotes steatosis and ferroptosis in non-alcoholic fatty liver disease via enhancing GPX4 ubiquitination. Hum Cell 2023;36:209–22. 36417114 10.1007/s13577-022-00820-3 PMC9813033

[CIT0375] Zhao S , El-Deiry WS. Identification of Smurf2 as a HIF-1α degrading E3 ubiquitin ligase. Oncotarget 2021;12:1970–9. 34611473 10.18632/oncotarget.28081 PMC8487721

[CIT0376] Zhao W , Steinfeld JB, Liang F et al. BRCA1–BARD1 promotes RAD51-mediated homologous DNA pairing. Nature 2017;550:360–5. 28976962 10.1038/nature24060 PMC5800781

[CIT0377] Zheng J , Chang L, Bao X et al. TRIM21 drives intervertebral disc degeneration induced by oxidative stress via mediating HIF-1α degradation. Biochem Biophys Res Commun 2021;555:46–53. 33813275 10.1016/j.bbrc.2021.03.088

[CIT0378] Zhou Q , Zhang J. K27-linked noncanonic ubiquitination in immune regulation. J Leukoc Biol 2022;111:223–35. 33857334 10.1002/JLB.4RU0620-397RR

[CIT0379] Zhou W , Xu J, Tan M et al. UBE2M is a stress-inducible dual E2 for neddylation and ubiquitylation that promotes targeted degradation of UBE2F. Mol Cell 2018;70:1008–1024.e6. 29932898 10.1016/j.molcel.2018.06.002 PMC6021141

[CIT0380] Zhu X , Zhang J, Sun H et al. Ubiquitination of inositol-requiring enzyme 1 (IRE1) by the E3 ligase CHIP mediates the IRE1/TRAF2/JNK pathway. J Biol Chem 2014;289:30567–77. 25225294 10.1074/jbc.M114.562868 PMC4215236

[CIT0381] Zhu T , Liu B, Wu D et al. Autophagy regulates VDAC3 ubiquitination by FBXW7 to promote erastin-induced ferroptosis in acute lymphoblastic leukemia. Front Cell Dev Biol2021a;9:740884. 34869326 10.3389/fcell.2021.740884 PMC8634639

[CIT0382] Zhu Y , Zhang C, Huang M et al. TRIM26 induces ferroptosis to inhibit hepatic stellate cell activation and mitigate liver fibrosis through mediating SLC7A11 ubiquitination. Front Cell Dev Biol2021b;9:644901. 33869196 10.3389/fcell.2021.644901 PMC8044755

[CIT0383] Zhu X , Xue J, Jiang X et al. TRIM21 suppresses CHK1 activation by preferentially targeting CLASPIN for K63-linked ubiquitination. Nucleic Acids Res 2022;50:1517–30. 35048968 10.1093/nar/gkac011 PMC8860585

